# A chemokine network of T cell exhaustion and metabolic reprogramming in renal cell carcinoma

**DOI:** 10.3389/fimmu.2023.1095195

**Published:** 2023-03-16

**Authors:** Renate Pichler, Peter J. Siska, Piotr Tymoszuk, Agnieszka Martowicz, Gerold Untergasser, Roman Mayr, Florian Weber, Andreas Seeber, Florian Kocher, Dominik A. Barth, Martin Pichler, Martin Thurnher

**Affiliations:** ^1^ Department of Urology, Comprehensive Cancer Center Innsbruck, Medical University of Innsbruck, Innsbruck, Austria; ^2^ Department of Internal Medicine III, University Hospital Regensburg, Regensburg, Germany; ^3^ Data Analytics As a Service Tirol, Innsbruck, Austria; ^4^ Department of Internal Medicine V (Hematology and Oncology), Comprehensive Cancer Center Innsbruck, Medical University of Innsbruck, Innsbruck, Austria; ^5^ Tyrolean Cancer Research Institute (TKFI), Medical University of Innsbruck, Innsbruck, Austria; ^6^ Department of Urology, Caritas St. Josef Medical Centre, University of Regensburg, Regensburg, Germany; ^7^ Department of Pathology, University of Regensburg, Regensburg, Germany; ^8^ Division of Oncology, Department of Internal Medicine, Medical University of Graz, Graz, Austria; ^9^ Research Unit for Non-Coding RNAs and Genome Editing, Medical University of Graz, Graz, Austria; ^10^ Immunotherapy Unit, Department of Urology, Medical University of Innsbruck, Innsbruck, Austria

**Keywords:** RCC, chemokines, immunotherapy, metabolism, OXPHOS, T cells, IDO, biomarker

## Abstract

Renal cell carcinoma (RCC) is frequently infiltrated by immune cells, a process which is governed by chemokines. CD8^+^ T cells in the RCC tumor microenvironment (TME) may be exhausted which most likely influence therapy response and survival. The aim of this study was to evaluate chemokine-driven T cell recruitment, T cell exhaustion in the RCC TME, as well as metabolic processes leading to their functional anergy in RCC. Eight publicly available bulk RCC transcriptome collectives (n=1819) and a single cell RNAseq dataset (n=12) were analyzed. Immunodeconvolution, semi-supervised clustering, gene set variation analysis and Monte Carlo-based modeling of metabolic reaction activity were employed. Among 28 chemokine genes available, *CXCL9/10/11/CXCR3, CXCL13/CXCR5 and XCL1/XCR1* mRNA expression were significantly increased in RCC compared to normal kidney tissue and also strongly associated with tumor-infiltrating effector memory and central memory CD8^+^ T cells in all investigated collectives. M1 TAMs, T cells, NK cells as well as tumor cells were identified as the major sources of these chemokines, whereas T cells, B cells and dendritic cells were found to predominantly express the cognate receptors. The cluster of RCCs characterized by high chemokine expression and high CD8^+^ T cell infiltration displayed a strong activation of IFN/JAK/STAT signaling with elevated expression of multiple T cell exhaustion-associated transcripts. Chemokine^high^ RCCs were characterized by metabolic reprogramming, in particular by downregulated OXPHOS and increased IDO1-mediated tryptophan degradation. None of the investigated chemokine genes was significantly associated with survival or response to immunotherapy. We propose a chemokine network that mediates CD8^+^ T cell recruitment and identify T cell exhaustion, altered energy metabolism and high IDO1 activity as key mechanisms of their suppression. Concomitant targeting of exhaustion pathways and metabolism may pose an effective approach to RCC therapy.

## 1 Introduction

Renal cell carcinoma (RCC) differs from other cancer entities by its high immunogenicity, which includes the efficient recruitment of tumor-infiltrating immune cells. As a result, the RCC tumor microenvironment (TME) harbors heterogenous mixtures of leukocyte subsets (1). Tumor-infiltrating lymphocytes (TILs) have previously attracted clinical research interest. In a form of adoptive T cell therapy, TILs were expanded ex vivo with high-dose IL-2 and subsequently re-infused into the patient (2). However, clinical efficacy of TIL therapy remained low. More recent studies demonstrated that CD8^+^ T cells in the RCC TME may be exhausted, express heterogenous phenotypes and express immune-evasive molecules (PD1, PD-L1, PD-L2, and CTLA4) (3, 4). In addition, the elevated CD8^+^ T cell presence corresponded to a higher frequency of BAP1 mutations (3), which is a key regulator of cancer-associated pathways (5). Exhausted T cells in the TME, which express multiple immune checkpoints, have been proposed to mediate resistance to immunotherapy with low therapeutic response rates (6–8). In RCC patients treated within the CheckMate-010 trial, a high percentage of CD8^+^ T cells expressing PD-1 but not TIM-3 and LAG-3 were positively associated with longer progression-free survival on the anti-PD-1 antibody nivolumab, suggesting that the higher predictive value of these cells might be related to their less exhausted phenotype and their ability to be more efficiently reactivated during PD-1 blockade (9). Additional immune cells such as CD4^+^CD25^+^FoxP3^+^ regulatory T cells (Tregs), myeloid-derived suppressor cells and tumor-associated macrophages (TAMs) (1) as well as dendritic cells (DCs) (10) have been detected in the RCC TME and may also affect anti-tumor immunity in different ways. Thus, the composition of TME-infiltrating immune cells and the functional phenotype of the individual infiltrating immune cell types are critical determinant factors in cancer prognosis and outcome.

The recruitment of immune cells to the TME is governed by chemokines (11), a large family of chemotactic cytokines, which are grouped into four subfamilies: CXC, CC, (X)C, and CX3C (12). Two members of the CXC family, CXCL9 (also known as MIG) and CXCL10 (also known as IP-10) are associated with Th1-type immune response by recruiting natural killer (NK) cells, CD4^+^ Th1 and CD8^+^ cytotoxic lymphocytes, which all contribute to anti-tumoral responses (13).

The migratory capacity of immune and non-immune cells is based on chemokine receptors that allow cells to migrate along chemokine gradients. In addition to being agonists of their cognate receptors, chemokines can also act as antagonists at other chemokine receptors. *CXCL9, CXCL10 and CXCL11*, for example are natural antagonists for *CCR3* (14).

In addition to their direct effects on anti-tumor immune responses, chemokines affect angiogenesis, cancer cell proliferation, stemness and invasiveness and can thus be key determinants of disease progression (15–17). All these observations make the chemokine/chemokine receptor network an attractive target for cancer immunotherapy. Moreover, chemokines and their receptors could be useful biomarkers of response and/or survival stratification.

Herein, published transcriptome data from eight publicly available bulk RCC collectives including 1819 cancer samples and a single cell RNAseq dataset were examined. We investigated the association between chemokine expression and T cell infiltration, metabolic changes and T cell in the RCC TME.

## 2 Methods

Detailed description of analysis procedures is provided in **Supplementary Material**.

### 2.1 Software

The analysis was done with R version 4.2.0. Basic data transformation tasks were accomplished with the tidyverse package bundle (18) and the development package trafo (https://github.com/PiotrTymoszuk/trafo). Exploratory data analysis and hypothesis testing was done with the rstatix (19), ExDA (https://github.com/PiotrTymoszuk/ExDA) and microViz (https://github.com/PiotrTymoszuk/microViz) packages. Network analysis was accomplished with igraph (20). Results were visualized with the packages ggplot2 (bubble plots) (21), ExDA (box plots), microViz (Forest plots, Volcano plots, bar plots of regulation estimates and p values), survminer (Kaplan-Meier plots) (22) and ggnetwork (network plots) (23).

### 2.2 Data import and transformation

The following publicly available RCC data sets were re-analyzed: TCGA Clear Cell Carcinoma (KIRC) project (24), whole-genome subsets of the CheckMate 010 (CM 010) and CheckMate 025 (CM 025) studies (25), GSE73731 (26), GSE167093 (27), RECA-EU (28) and E-MTAB 1980 (29). Author-provided expression data and clinical information for the TCGA cohort were extracted from the GDC Data Portal with the TCGA-Assembler script (https://github.com/compgenome365/TCGA-Assembler-2/blob/master/TCGA-Assembler/). Author-provided expression and clinical data for GSE73731 and GSE167093 were fetched with the *GEOquery* package (30). E-MTAB 1980 and RECA-EU data sets were imported from the ArrayExpress and ICGC Data Portal repositories, respectively, with in-house developed R scripts. To investigate possible effects of therapy with checkpoint inhibitors, the CheckMate 025 everolimus (CM 025 EVER) and nivolumab (CM 025 NIVO) treatment arms were analyzed separately.

For the microarray expression studies (GSE73731, GSE167093, E-MTAB 1980), integration of multiple probes targeting the same gene was accomplished by geometric mean. Expression values were transformed with *log*_2_(*transcriptcount* + 1) (RNA sequencing: TCGA, CM 010, CM 025, RECA-EU) of *log*_2_. Immune infiltration estimates were calculated using the QuanTIseq and xCell algorithms (*immunedeconv* package) (31–33).

Gene signatures corresponding to Reactome pathways were extracted from the MSig database, version 7.5.1, and the signatures’ single sample gene set enrichment analysis scores (ssGSEA) were calculated with the *GSVA* algorithm (34). Genes associated with T cell exhaustion were retrieved from four recent papers (35–38).

### 2.3 Genes of interest

The chemokine genes of interest were identified within the set of 28 chemokine genes available for all investigated datasets (CCL2, CCL7, CCL8, CCL11, CCL13, CCL17, CCL20, CCL21, CCL22, CCL24, CCL25, CCL26, CCL28, CXCL1, CXCL2, CXCL3, CXCL5, CXCL6, CXCL9, CXCL10, CXCL11, CXCL12, CXCL13, CXCL14, CXCL16, CXCL17, CX3CL1, XCL1). The chemokine genes were screened in the TCGA KIRC cohort for differences in expression levels between the RCC and normal kidney tissue (paired normal/tumor samples, two-tailed paired T test, function test_two_groups(), microViz package), and for correlation with CD8^+^ T cell content in RCC predicted with the QuanTIseq algorithm (31) (Spearman’s correlation, correlate_variables(), package ExDA).

Among 28 chemokine genes measured in all investigated collectives, expression of *CXCL9*, *CXCL10*, *CXCL11*, *CXCL13* and *XCL1* was found to be highly enriched in the malignant tissue (paired two-tailed T test) and strongly significantly associated with CD8^+^ T cell levels (Spearman’s correlation) in the TCGA KIRC cohort (**Supplementary Figure S1**). These chemokine genes and their cognate receptors *CXCR3* (CXCL9/10/11), *CXCR5* (CXCL13) and *XCR1* (XCL1) were investigated further in the current report.

### 2.4 Comparison of gene expression between the normal kidney and tumor samples, correlation with non-malignant cell infiltration

log_2_-transformed expression was compared between the donor-matched normal kidney and cancer samples by two-tailed paired T test and Cohen’s d effect size statistic. Correlation of log_2_-transformed gene expression with QuantIseq- and xCell-predicted non-malignant cell content (31, 33) was investigated with Spearman’s test. The expression comparison and correlation results were corrected for multiple testing with the false discovery rate (FDR) method (39). The scaled Spearman’s correlation matrices for the genes of interest and cell types associated with them with moderate-to-large strength (correlation coefficient *ρ* > 0.4) were visualized as force-directed network plots (packages *igraph* and *ggnetwork*) (20, 23).

### 2.5 Survival modeling

Correlation of log_2_-transformed gene expression with overall (OS) or relapse-free survival (RFS) by uni-variable Cox proportional hazard modeling (40) including the linear and spline term for the gene expression variable. Significance for model terms was corrected with FDR for multiple testing. Linear term model estimates are presented as hazard ratios (HR) with 95% confidence intervals.

Multi-parameter modeling of OS was done by Ridge regularized Cox regression (package *glmnet*) (41). The explanatory factors included age, squared age, sex, tumor grade, tumor stage, metastasis stage) variables and log_2_-transformed expression values of the genes of interest. Two models were trained in the TCGA KIRC cohort: a model with demographic/grade/stage variables only and a model with the complete set of explanatory factors and both models were subsequently validated in the E-MTAB 1980 and RECA-EU collectives. To assess the add-on effect of gene expression on the survival prediction accuracy as compared with the demographic/grade/stage information, concordance indexes of the full models and the demographic/grade/stage-only models were compared.

### 2.6 Semi-supervised clustering

By PAM (partition around medoids) - cosine distance clustering (42, 43) in respect to to normalized log_2_-transformed expression of the genes of interest, two clusters of RCC samples, the chemokine^high^ and chemokine^low^ cluster were defined in the training TCGA KIRC cohort. The clustering algorithm was chosen based on its excellent reproducibility in 10-fold cross validation (44) and good explanatory performance as compared with hierarchical and KMEANS clustering algorithms (**Supplementary Figure S2A**). Choice of the cluster number (k = 2) was motivated by the bend of the within-cluster sum of squares curve, the peak mean silhouette statistic and visual inspection of the heat map of distances between cancer samples (**Supplementary Figures S2B, C**).

Prediction of the chemokine cluster assignment in the test collectives (E-MTAB 1980, GSE73731, GSE167093, RECA-EU and CheckMate cohorts) was done with a 15-nearest neighbor (15-NN) classifier with inverse distance weighting. The prediction yielded clustering structures with comparable fractions of explained variance and comparable cluster sizes in the training and the test cohorts indicative of high reproducibility (**Supplementary Figure S3A**).

xCell estimates of non-malignant cell infiltration (43) were compared between the chemokine clusters by FDR-corrected Mann-Whitney U test. Differences in distribution of tumor stages, MSKCC risk strata and frequencies of best overall therapy response (complete/partial response vs. stable/progressive disease) between the clusters were compared by FDR-corrected *χ* ^2^ test. Differences in OS and RFS between the two clusters were assessed by Peto-Peto test (22).

### 2.7 Differences in Reactome pathways, gene expression and signaling modulation between the clusters

Differences in ssGSEA scores (34) of Reactome pathway gene signatures between the chemokine clusters were investigated by FDR-adjusted two-tailed T test (**Supplementary Table S1**). Genes differentially expressed between the chemokine clusters were identified by FDR-corrected two-tailed T test and > 1.25 fold-regulation in the chemokine high versus chemokine low cluster (**Supplementary Tables S2, S3**). Modulation of KEGG-listed signaling pathways in chemokine^high^ versus chemokine^low^ cluster cancers based on the differential gene expression was investigated with *SPIA* (45) (**Supplementary Table S4**).

### 2.8 Biochemical reaction modulation in the CXCL9 expression strata

Rules of assignment of genes to biochemical reactions were retrieved from the Recon2 human metabolism model available *via* the BiGG database (46). Regulation of biochemical reactions between the chemokine^high^ and chemokine^low^ clusters based on whole-genome estimates of differential gene expression and their standard errors was done by evaluation of the gene assignment rules with the ‘gene - protein - reaction’ (GPR) principle (47). Standard deviation, 95% confidence intervals and p values for the predicted reaction regulation estimates were obtained by a Monte Carlo simulation (n = 1000 draws) (**Supplementary Table S5**). The analysis was done with the package *BiGGR* (47) and the development package *biggrExtra* (https://github.com/PiotrTymoszuk/biggrExtra).

### 2.9 In-house flow cytometry

Peripheral blood mononuclear cells, adjacent normal kidney tissues, tissues from the center and periphery of ccRCC tumors (**Supplementary Table S6**; n=4) were obtained freshly at the day of surgery. Patients were all treated at the University Hospital Regensburg (Urology) between 2019 and 2020. Tissues were processed as described previously (48). Peripheral blood was enriched using leukocyte reduction system cones and processed using Ficoll density gradient centrifugation. After processing, single-cell suspensions were cryopreserved until the day of analysis. For flow cytometric analyses, cells were permeabilized using the Cytofix/Cytoperm kit (BD) and stained with antibodies against: CD3, CD14, CD56, CD19, CD25, CD4 (all BD); CD8 (BioLegend), CXCL9, (Biotechne); and with Fixable Viability Dye eFluor 708 (eBioscience). Flow cytometry was performed using LSRFortessa (BD).

### 2.10 Single-cell RNAseq analysis

The respective dataset consisting of samples obtained from 12 patients with RCC was downloaded as AnnData object (h5ad) from previously published studies of Li R. et al. (49) [Dataset (50)] and imported in Scanpy version 1.9.1. The dataset was controlled for the quality with scanpy by thresholding the number of detected genes (200), counts (2000) and the fraction of mitochondrial reads (<30%).

Cell transcriptomes were embedded into a batch-corrected low-dimensional latent space using scVI (51, 52) treating each sample as a separate batch. The scVI model was trained on the 2000 most ‘highly variable genes’ as determined with scanpy’s “*pp*.*highly_variable_genes*’ with parameters ‘*flavor=“seurat’,* and *batch_key=‘orig.ident’*. A neighborhood graph and UMAP embedding was computed based on the scVI latent space. All cell-type annotations were used from the original study (49). Annotated cell types were confirmed by a set of cell type–specific markers such as, CD3E, CD68, CD8A, CD4, CD79A, KIT, CDH5, ACTA2, EPCAM. For more detailed analysis ‘tumor’ and ‘normal-kidney’ samples were extracted from the dataset and merged into a sparate AnnData object.

Data analysis and graphical visualization was performed with scanpy v.1.9.1, anndata v.0.8.0, umap v.0.5.3, numpyv.1.21.5, scipy v.1.7.3, pandas v.1.4.2, scikit-learn v.1.02.2, statsmodels v.0.13.2, pynndescent v.0.5.7, and python-igraph v.0.10.2.  A method and samples overview of scRNA dataset is shown in **Supplementary Figure S4**.

#### 2.10.1 Multiplex immunofluorescence

In-House RCC samples (*n* = 4) after surgery were fixed in 4% paraformaldehyde, embedded in paraffin and five-micrometer sections were used for the immunofluorescence staining. Multiplex IHC was performed using Opal 6-plex Detection Kit (cat: NEL821001KT, Akoya Biosciences, Menlo Park, USA). A multiplex panel of immune markers was developed with antibodies against CXCR3 (clone EPR25373-32, cat: ab288437, dilution 1:200, Abcam, Cambridge, MA, USA), CD4 (clone EP204, cat: 104R-26, dilution 1:50, Cell Marque), CD8 (clone C8/144B, cat: M710301-2, dilution 1:200, Dako/Agilent, Santa Clara, CA, USA), CD68 (clone PG-M1, cat: M087601-2, dilution 1:250, Dako/Agilent), cytokeratin (clone AE1/AE3, cat: MA5-13156, dilution 1:500, Thermo-Fisher). The staining procedure was performed using an automated staining system (BOND-RX; Leica Biosystems, Vienna, Austria). To visualize cell nuclei, the tissue was stained with 4’,6-diamidino-2-phenylindole (spectral DAPI, Akoya Biosciences). Slides were scanned at 20x magnification using Mantra 2 Quantitative Pathology Workstation (Akoya Biosciences) and representative images from each tissue were acquired with the Mantra Snap software version 1.0.4. Image spectral deconvolution, multispectral image analysis and cell phenotyping was carried out using the InForm Tissue Analysis Software version 2.4.10 (Akoya Biosciences).

## 3 Results

### 3.1 Characteristic of the study cohorts

Altogether, we analyzed clinical and transcriptome data from 8 publicly available RCC cohorts including a total of 1819 RCC samples. Males constituted between 57 and 76% of investigated patients and the gender distribution was comparable between the cohorts. The median age ranged between 60 and 64 years and was similar in the study collectives. The RECA-EU, GSE167093 and E-MTAB 1980 cohort individuals tended towards lower tumor grades (G2) as compared with the remaining collectives with the grade data available. The majority of tumors was stage T1. In the TCGA, RECA-EU and E-MTAB 1980, information on initial metastasis status was available; only a small fraction of participants had lymph node or distant metastases at RCC diagnosis. The median observation time was clearly shorter in the CM 010 and CM 025 collectives. In these cohorts, the rate of relapses and mortality within the observation time window was substantially higher. In the samples from the CM studies, between 5.1 (CM 025 everolimus) and 25% patients (CM 010 and CM 025, nivolumab) displayed an overall therapy response defined as CR or PR (**Table 1**).

**Table 1:** Characteristic of the study cohorts. Numeric variables are presented as medians with interquartile ranges and ranges.

Variable	TCGA	EMTAB1980	GSE73731	GSE167093	RECA	CM010	CM025eve	CM025niv
Sex	Female: 35% (n = 188) Male: 65% (n = 345) complete: n = 533	Female: 24% (n = 24) Male: 76% (n = 77) complete: n = 101	Female: 39% (n = 102) Male: 61% (n = 160) complete: n = 262	Female: 41% (n = 247) Male: 59% (n = 357) complete: n = 604	Female: 43% (n = 39) Male: 57% (n = 52) complete: n = 91	Female: 33% (n = 15) Male: 67% (n = 30) complete: n = 45	Female: 27% (n = 25) Male: 73% (n = 67) complete: n = 92	Female: 24% (n = 21) Male: 76% (n = 67) complete: n = 88
Age, years	61 [IQR: 52 - 70] range: 26 - 90 complete: n = 533	64 [IQR: 56 - 72] range: 35 - 91 complete: n = 101		62 [IQR: 55 - 68] range: 23 - 85 complete: n = 602	60 [IQR: 54 - 67] range: 35 - 83 complete: n = 91	61 [IQR: 55 - 67] range: 46 - 81 complete: n = 45	62 [IQR: 56 - 68] range: 31 - 86 complete: n = 92	62 [IQR: 53 - 68] range: 30 - 88 complete: n = 88
Tumor grade	G1: 2.6% (n = 14) G2: 43% (n = 229) G3: 39% (n = 206) G4: 14% (n = 76) GX: 0.94% (n = 5) complete: n = 530	G1: 13% (n = 13) G2: 60% (n = 59) G3: 22% (n = 22) G4: 5.1% (n = 5) complete: n = 99	G1: 8.6% (n = 22) G2: 35% (n = 90) G3: 37% (n = 95) G4: 19% (n = 49) complete: n = 256	G1: 19% (n = 100) G2: 57% (n = 304) G3: 20% (n = 105) G4: 4.5% (n = 24) complete: n = 533	G1: 14% (n = 13) G2: 53% (n = 48) G3: 17% (n = 15) G4: 16% (n = 14) complete: n = 90			
Tumor stage	T1: 51% (n = 273) T2: 13% (n = 69) T3: 34% (n = 180) T4: 2.1% (n = 11) complete: n = 533	T1: 67% (n = 68) T2: 11% (n = 11) T3: 21% (n = 21) T4: 0.99% (n = 1) complete: n = 101	T1: 33% (n = 41) T2: 9.6% (n = 12) T3: 22% (n = 28) T4: 35% (n = 44) complete: n = 125	T1: 51% (n = 306) T2: 16% (n = 98) T3: 23% (n = 138) T4: 10% (n = 62) complete: n = 604	T1: 59% (n = 54) T2: 14% (n = 13) T3: 24% (n = 22) T4: 2.2% (n = 2) complete: n = 91			
Metastasis stage	M0: 79% (n = 422) M1: 15% (n = 79) MX: 5.6% (n = 30) complete: n = 531	M0: 88% (n = 89) M1: 12% (n = 12) complete: n = 101			M0: 89% (n = 81) M1: 9.9% (n = 9) MX: 1.1% (n = 1) complete: n = 91			
Node stage, pn	N0: 45% (n = 240) N1: 3% (n = 16) NX: 52% (n = 277) complete: n = 533	N0: 93% (n = 94) N1: 3% (n = 3) N2: 4% (n = 4) complete: n = 101			N0: 87% (n = 79) N1: 2.2% (n = 2) NX: 11% (n = 10) complete: n = 91			
Relapse	26% (n = 115) complete: n = 435	32% (n = 32) complete: n = 101			6.6% (n = 6) complete: n = 91	91% (n = 41) complete: n = 45	89% (n = 82) complete: n = 92	88% (n = 77) complete: n = 88
Death	30% (n = 160) complete: n = 530	23% (n = 23) complete: n = 101			33% (n = 30) complete: n = 91	78% (n = 35) complete: n = 45	83% (n = 76) complete: n = 92	70% (n = 62) complete: n = 88
Observation time, days	1000 [IQR: 330 - 1700] range: 0 - 3800 complete: n = 529	1600 [IQR: 1000 - 2500] range: 30 - 4400 complete: n = 101			1800 [IQR: 1100 - 2000] range: 2 - 2300 complete: n = 91	770 [IQR: 240 - 1500] range: 36 - 2200 complete: n = 45	600 [IQR: 250 - 1100] range: 21 - 1900 complete: n = 92	680 [IQR: 350 - 1500] range: 25 - 2000 complete: n = 88
MSKCC risk group						favorable: 36% (n = 16) intermediate: 38% (n = 17) poor: 27% (n = 12) complete: n = 45	favorable: 36% (n = 33) intermediate: 47% (n = 43) poor: 17% (n = 16) complete: n = 92	favorable: 27% (n = 24) intermediate: 50% (n = 44) poor: 23% (n = 20) complete: n = 88
Therapy response						CRPR: 0% (n = 0) complete: n = 45	CRPR: 5.1% (n = 4) complete: n = 79	CRPR: 25% (n = 21) complete: n = 83

Categorical variables are presented as percentage and total number within the complete observation set.

### 3.2 *CXCL9/10/11*, *CXCL13* and *XCL1* govern CD8^+^ T cell recruitment in RCC

First, we sought to identify RCC-specific chemokines which may mediate CD8^+^ T cells recruitment. Expression of 28 chemokine genes was compared between RCC and normal kidney, and correlated with CD8^+^ T cell infiltration predicted by the QuanTIseq algorithm (46) in the TCGA KIRC cohort. *CXCL9/10/11*, *CXCL13* and *XCL1* were found to be at least four-fold upregulated in the cancer tissue and correlated strongly with CD8^+^ T cell infiltration with *ρ* > 0.6 in Spearman test (**Supplementary Figure S1**). These chemokine genes along with genes coding for the cognate receptors *CXCR3* (CXCL9/10/11), *CXCR5* (CXCL13) and *XCR1* (XCL1) were analyzed further.

**Figure 1:**
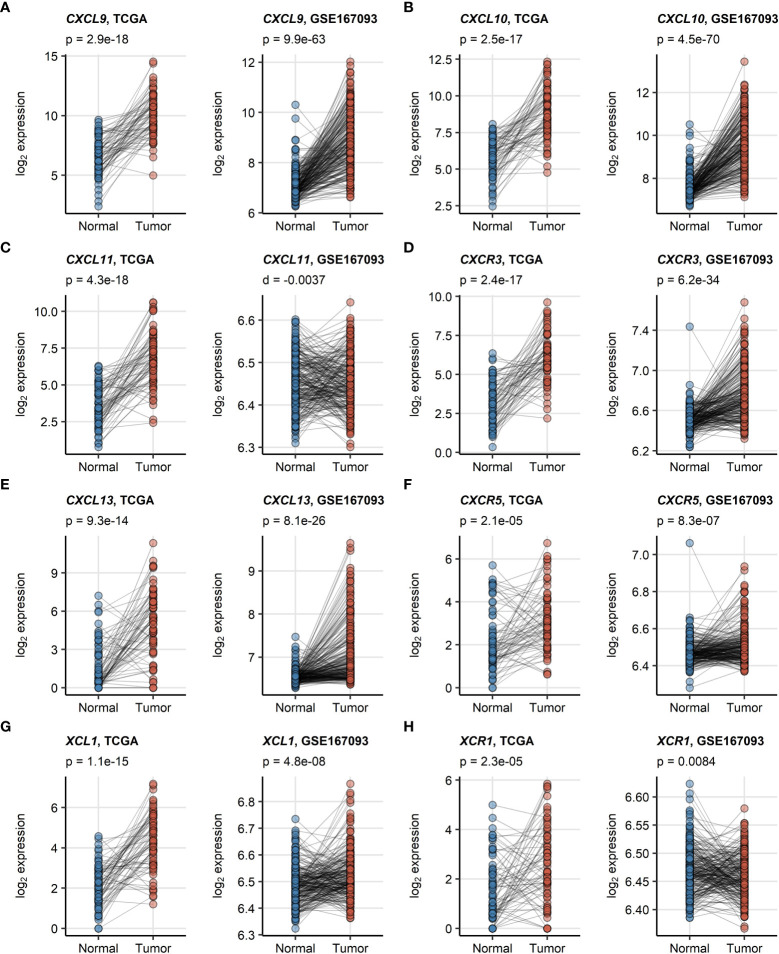
**(A–H)** Differences in expression of *CXCL9/10/11*, *CXCL13*, *XCL1* and their cognate receptors between RCC and normal kidney tissue. Differences in expression of CXCL9/10/11, CXCR3, CXCL13, CXCR5, XCL1 and XCR1 between the RCC and normal kidney tissue were investigated in donor matched samples from the TCGA KIRC and GSE167093 cohorts by two-tailed paired T test. The results were corrected for multiple testing with false discovery rate (FDR) method. Points represent single samples, samples from the same tissue donors are connected with lines. P values are displayed in the plot captions. Numbers of tissue donors are indicated under the plots.

*CXCL9/10/13* and *XCL1* chemokine transcripts and the *CXCR3*, *CXCR5* receptor transcripts were significantly enriched in RCC as compared with the non-malignant tissue in an analysis of donor-matched samples in the TCGA KIRC and GSE167093. This upregulation was particularly strong for *CXCL9*, *CXCL10* and *CXCL13*. In turn, significantly elevated expression of *CXCL11* and *XCR1* in RCC as compared with normal kidney tissue could be observed only in the TCGA KIRC cohort but not in the GSE167093 collective (**Figure 1**).

**Figure 2:**
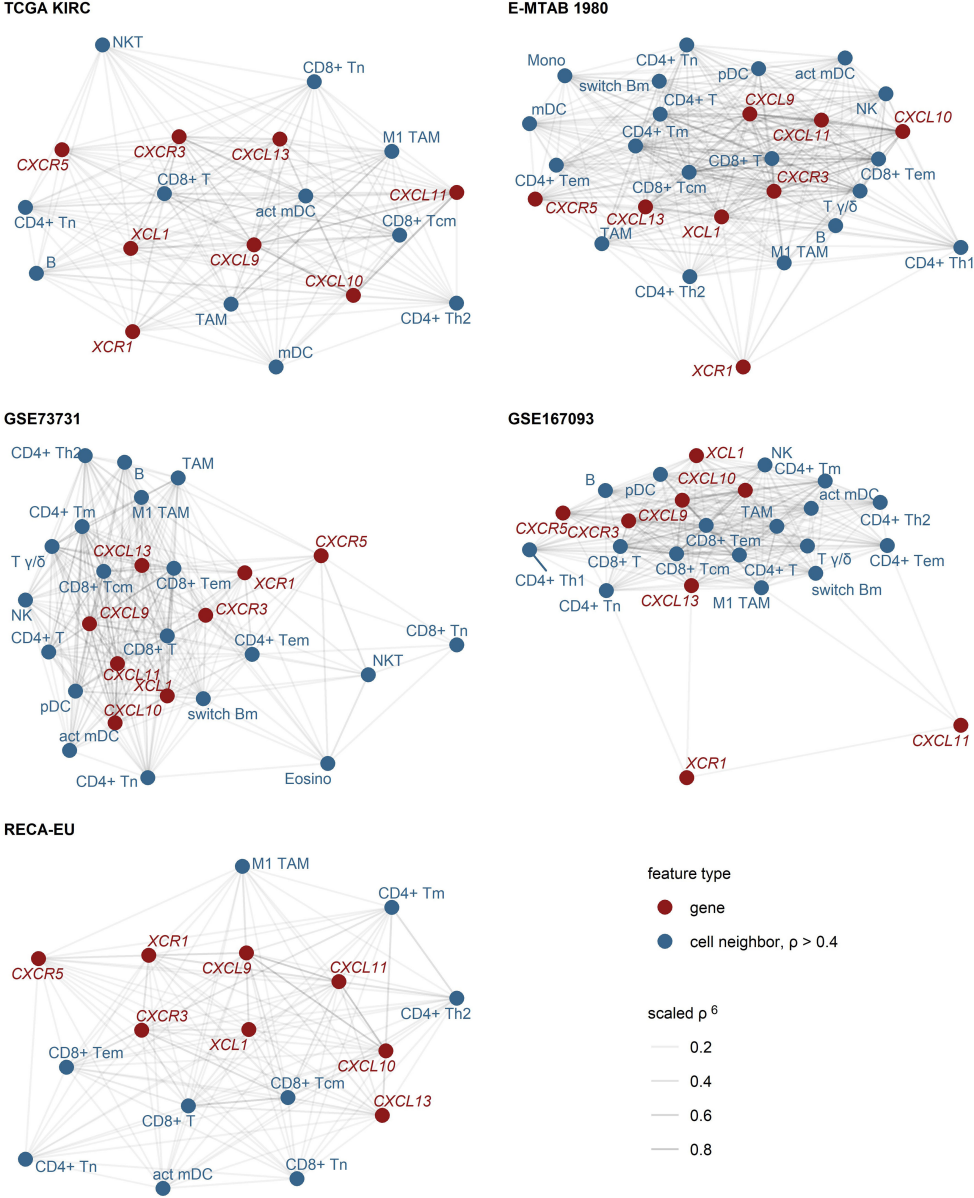
Expression of *CXCL9/10/11*, *CXCL13*, *XCL1* and their cognate receptors in RCC samples enriched in CD8^+^ and CD4^+^ T cells, activated myeloid DC, TAM and B cells. Pairwise association of the gene expression and xCell non-immune cell infiltration estimates was assessed by scaled Spearman’s correlation. The genes of interest and the cell types associated with the genes of interest with moderate-to-large strength (correlation coefficient ρ > 0.4) were visualized as network force-directed plots with edge weighting by the scaled Spearman’s correlation coefficient. CD8^+^ T, CD8^+^ T cells, CD4^+^ T, CD4^+^ T cells, n, naive; m, memory; em, effector memory; cm, central memory; Th1, T helper cells 1; Th1, T helper cells 1; mDC, myeloid dendritic cells, act mDC, activated mDC; pDC, plasmacytoid dendritic cells; TAM, tumor-associated macrophages; B, B cells, switch Bm, class-switched memory B cells; NK, natural killer cells; NKT, natural killer T cells; T γδ, γδ T cells; Mono, monocytes; Eosino, eosinophils.

As revealed by network analysis of Spearman’s correlations between gene expression and non-malignant cell content predicted by the xCell algorithm, expression of *CXCL9/10/11*, *CXCR3*, *CXCL13*, *XCL1* and *XCR1* was tightly associated with increased infiltration by panCD8^+^, central memory and effector memory CD8^+^ T cells, activated myeloid and plasmacytoid DC (mDC and pDC), TAMs, CD4^+^ T cells and B cells (**Figure 2**; **Supplementary Figure S5**). The association of CD8^+^ T cells with *CXCL9*/10/13 expression was the strongest. Substantially weaker correlations were detected for CD8^+^ T cells and *CXCR5* or *XCR1* (**Supplementary Figures S6, S7**). Likewise, highly reproducible correlation could be observed between RCC expression of the genes of interest and CD8^+^ T cell infiltration levels as predicted by the QuanTIseq algorithm (31) (**Supplementary Figures S8, S9**). Interestingly, levels of chemokines and the cognate receptors were not associated with Treg infiltration.

### 3.3 Chemokine expression is enriched in immune and tumor cells, whereas the cognate receptors are exclusively produced by immune cells

**Figure 3:**
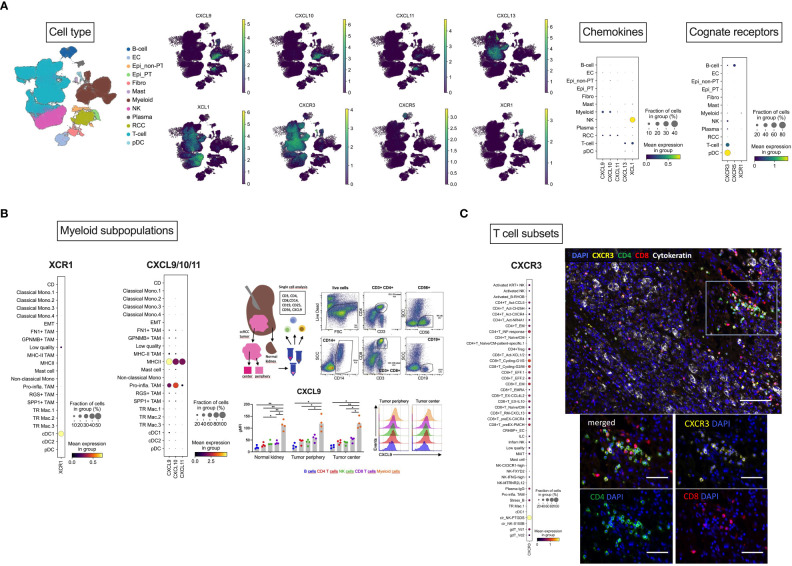
Cellular localization of *CXCL9/10/11, CXCL13, XCL1* and their cognate receptors by scRNAseq analysis. **(A)** Overview of cellular localization of chemokines (CXCL9/10/11, CXCL13 and XCL1) and the cognate receptors (CXCR3, CXCR5 and XCR1). **(B)** Dotplot expression analysis of annotated myeloid subpopulations in the dataset (49) with targets CXCL9/10 and XCR1 demonstrating dendritic cells as the major source of XCR1 and M1 TAMs for CXCL9/10. As a representative member of the chemokine cluster, CXCL9 was further analyzed by flow cytometry. RCC tumors from tumor center and tumor periphery and matched normal adjacent kidney tissues were resected and freshly processed to obtain a single-cell suspension. Cells were analyzed using flow-cytometry. Dotplots of a representative gating of live cells are shown: CD3^+^CD4^+^ and CD3^+^CD8^+^ T cells, CD56^+^ NK cells, CD19^+^ B cells and CD14^+^ myeloid cells. Levels of *CXCL9* were assessed in the specific immune cell subpopulations. FACS identified CD14^+^ tumor-infiltrating myeloid cells as the population with the highest levels of CXCL9 protein. **(C)** Dotplot expression analysis of annotated T cell subsets in the dataset with the target CXCR3 showing CD4^+^ and CD8^+^ T cells as the major source of CXCR3. This finding was also confirmed by multiplex immunofluorescence analysis. Multiplex immunofluorescence image of RCC showing coexpression of CXCR3 with CD8 and CD4 T cells. Scale bar = 100 μm. **(B)** Magnification of TILs region. Scale bar = 50 μm. Stastististical tests used: **(A)** two-way ANOVA with Geisser-Greenhouse correction with Tukey’s multiple comparisons test; *p<0.05, **p<0.01.

To further delineate expression of the chemokines of interest and the cognate receptors at the single-cell level, we reanalyzed a publicly available PDAC dataset (49), comprising single-cell RNAseq data from 12 RCC samples. Following cell type annotation, re-analysis revealed CXCL9 as well as CXCL10 expression mainly in myeloid cells, whereas CXCL11 was exclusively expressed in tumor cells. T cells were the major source of CXCL13, while XCL1 was predominantly expressed in NK cells. Focusing on the cognate receptors, CXCR3 was mainly expressed in T cells, CXCR5 in B cells and finally, XCR1 in myeloid cells (**Figure 3A**). In-depth analysis of CXCL9/10 and XCR1 expression in different myeloid subclusters showed that myeloid/conventional DC (cDC) were the majour source of CXCR1. On the contrary, CXCL9 and CXCL10 were predominantly expressed in M1 TAMs. In line with this finding, our FACS of tissue samples from periphery and center of RCC tumors (**Appendix Table S6**) also identified CD14^+^ tumor-infiltrating myeloid cells as the population with the highest levels of CXCL9 protein (**Figure 3B**). In-depth analysis of CXCR3 expression in T cell subsets revealed CXCR3 expression in CD4^+^ and CD8^+^ T cells. Immunofluorescence experiments of in-house RCC samples again corroborated that CXCR3 is mainly co-expressed on CD4^+^ and CD8^+^ T cells (**Figure 3C**).

### 3.4 Clustering according to chemokine expression

**Figure 4:**
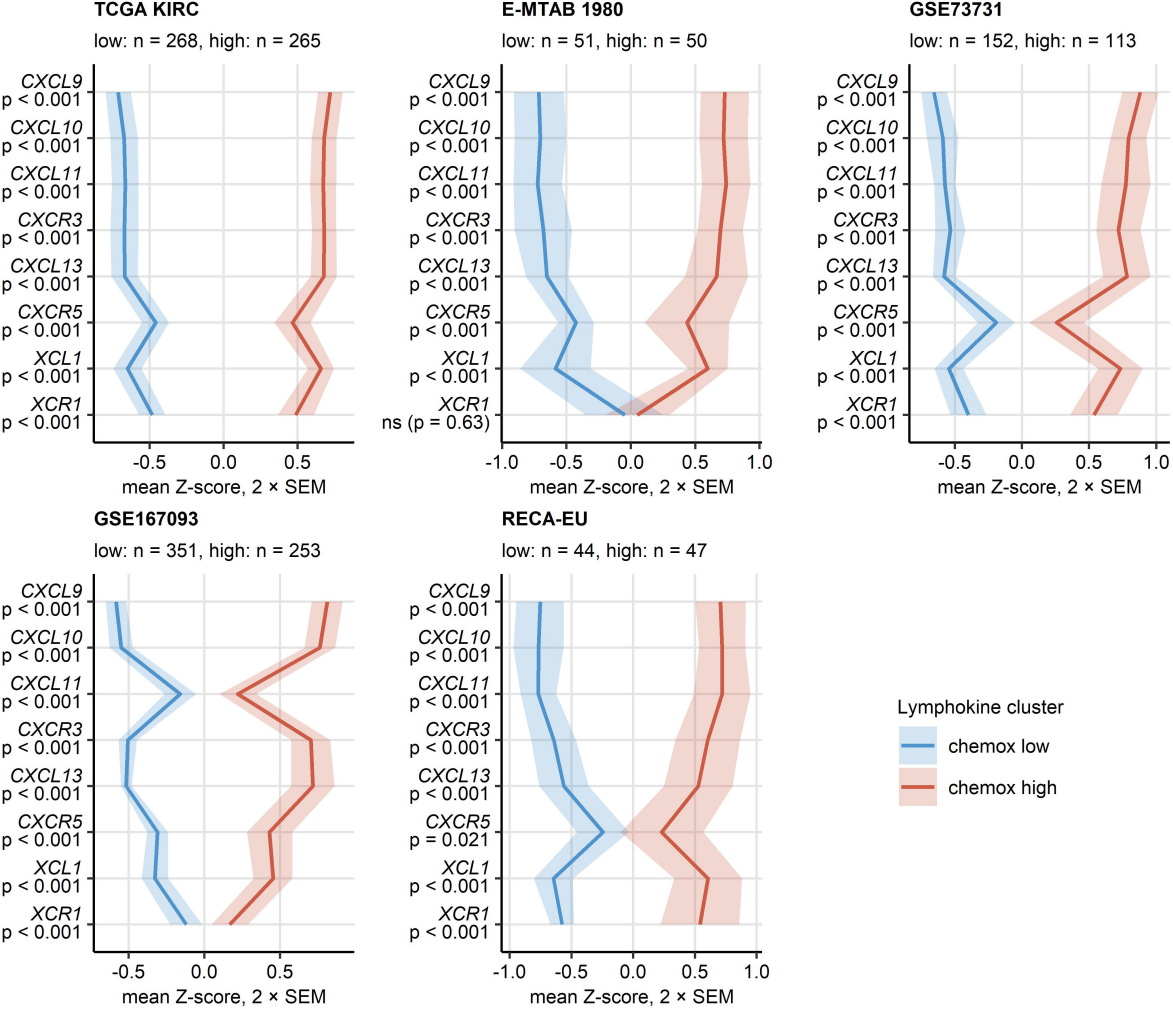
Expression of CXCL9/10/11, CXCL13, XCL1 and their cognate receptors in the two chemokine clusters. Chemokine clusters of RCC samples were defined in the TCGA KIRC training cohort in respect to normalized log_2_-transformed expression of CXCL9/10/11, CXCR3, CXCL13, XCL1 and XCR1 by the PAM/cosine distance algorithm. Prediction of cluster assignment in the E-MTAB 1980, GSE73731, GSE167093, RECA-EU, CM 010, CM 025 everolimus and CM 025 nivolumab (CM 025 NIVO) was done with the inverse distance-weighted 15-nearest neighbor classifier. log_2_ transformed expression of the clustering genes was compared between the chemokine (chemox) high and low cluster by false discovery rate (FDR) corrected two-tailed T test in the TCGA KIRC, E-MTAB 1980, GSE73731, GSE167093, RECA-EU collectives. Mean normalized expression values are visualized as thick lines, tinted regions represent two standard errors of the mean (SEM). P values are shown in the Y axis. Numbers of cancer samples assigned to the clusters are displayed in the plot captions.

**Figure 5:**
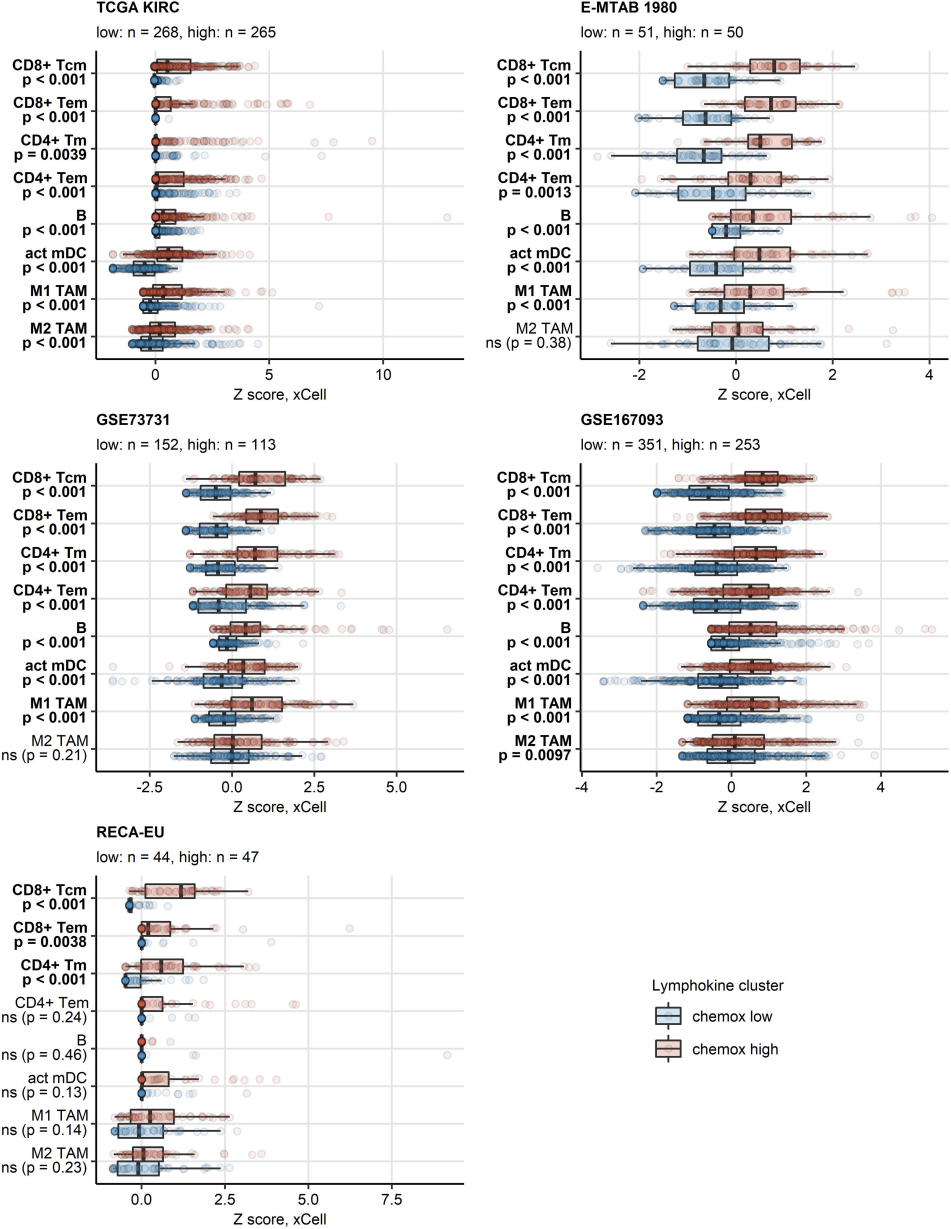
Predicted infiltration of T and B cells, activated mDC and TAM in the two chemokine clusters. Differences in levels of effector memory CD8^+^ T (CD8^+^ Tem), central memory CD8^+^ T (CD8^+^ Tcm), memory CD4^+^ T (CD4^+^ Tm), effector memory CD4^+^ T cells (CD4^+^ Tem), B cells **(B)**, activated myeloid dendritic cells (act mDC), M1 and M2 tumor-associated macrophages (TAM) between the chemokine (chemox) high an chemokine low cluster were assessed by false discovery rate (FDR) corrected Mann-Whitney test in the TCGA KIRC, E-MTAB 1980, GSE73731, GSE167093, RECA-EU collectives. Median normalized infiltration levels with interquartile ranges (IQR) are presented as boxes, whiskers span over the 150% IQR. Points represent single cancer samples. P values are indicated in the Y axes, significant effects are highlighted in bold. Numbers of cancer samples assigned to the chemokine high and low clusters are displayed in the plot captions.

PAM clustering of the TCGA KIRC samples in respect to normalized expression levels of *CXCL9/10/11*, *CXCR3*, *CXCL13*, *XCL1* and *XCR1* yielded two distinct subsets of RCC termed the chemokine^high^ and chemokine^low^ cluster (**Supplementary Figure S2**). This clustering structures were highly conserved in the remaining investigated collectives as evident from comparable fractions of explained clustering variance and similar distribution of chemokine^high^ and chemokine^low^ sample frequencies within the cohorts (**Supplementary Figure S3A**). The strongest differences in expression of the clustering genes between the chemokine^high^ and chemokine^low^ cluster were detected for *CXCL9/10/11*, *CXCR3* and *CXCL13* (**Figure 4**; **Supplementary Figure S3B**). In line with the correlation results, xCell-predicted infiltration of central memory CD8^+^ T cells was significantly higher in the chemokine^high^ than in the chemokine^low^ cluster. Levels of xCell-predicted TAM, activated mDC, RCC and B cell infiltration were significantly upregulated in chemokine^high^ cancers in the majority of analyzed cohorts (**Figure 5**; **Supplementary Figure S10**).

### 3.5 Chemokine^high^ expression shows no influence on survival and therapy response

**Figure 6:**
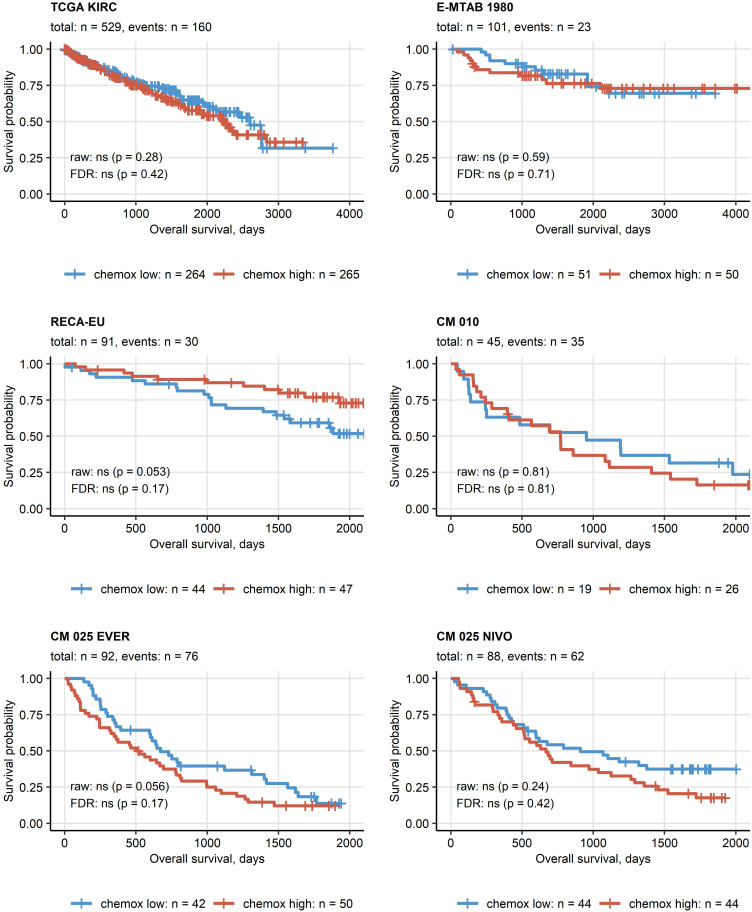
Overall survival in the two chemokine clusters. Differences in overall survival between the chemokine (chemox)^high^ and chemox^low^ RCCs were investigated by false discovery rate (FDR) corrected Peto-Peto test. Fractions of surviving patients are visualized in Kaplan-Meier plots. Uncorrected and FDR-corrected p values are displayed in the plots. Number of complete observations and deaths (events) are displayed in the plot captions, numbers of patients in the clusters are indicated under the plots. CM 010: CheckMate 010; CM 025 EVER: CheckMate 025 everolimus; CM 025 NIVO: CheckMate 025 nivolumab.

Except for the TCGA KIRC cohort displaying a significant enrichment of late stage tumors in the chemokine^high^ cluster, no significant differences in distribution of tumor stages and MSKCC risk groups could be observed neither between the two clusters (**Supplementary Figure S11**) nor at the level of single chemokine genes (data not shown). No differences in best therapy response between the chemokine clusters could be detected neither for everolimus nor for nivolumab in the CM collectives (**Supplementary Figure S12**). Similarly, OS and RFS times in the two clusters did not differ significantly (**Figure 6**; **Supplementary Figure S13**).

Finally, in multi-parameter Ridge Cox modeling of OS as a function of age, sex, grade, tumor and metastasis stage together with gene expression, the influence of chemokine levels was found to be marginal as compared with age, grade, tumor and metastasis stage (**Supplementary Figure S14A**). As evident from a comparison of concordance indexes of the multi-parameter model consisting of solely clinical and demographic variables and the model including gene expression parameters, there was no add-on value of the investigated gene expression levels to the prediction accuracy of the OS in the TCGA KIRC, E-MTAB 1980 or RECA-EU collectives (**Supplementary Figure S14B**).

### 3.6 Chemokine^high^ cluster is associated with IFN signaling and T cell exhaustion

**Figure 7:**
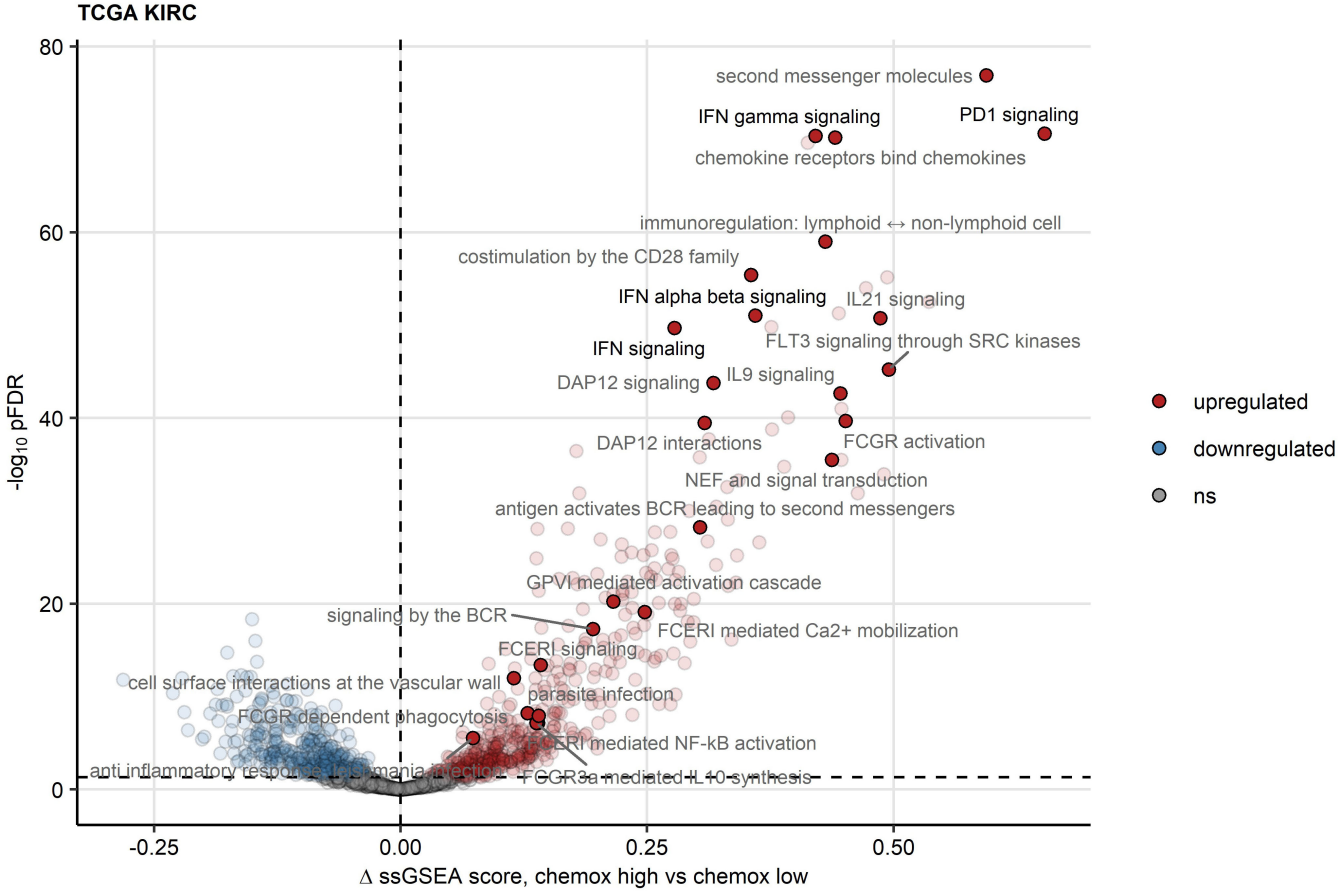
Reactome pathway gene signatures significantly regulated between the two chemokine clusters. Single sample gene set enrichment analysis (ssGSEA) scores for Reactome pathway gene signatures (n = 1615) were compared between chemokine (chemox)^high^ and chemokine^low^ RCCs by false discovery rate (FDR) corrected two-tailed T test. Significance and estimated differences in ssGSEA scores for the TCGA KIRC cohort are presented in a volcano plot. Points represent single genes, point color codes for the regulation sign. The significance cutoff is visualized as a dashed line. Reactome pathway signatures found to be significantly regulated in all investigated cohorts are highlighted and labeled with their names.

To elucidate such mechanisms of activation and suppression of CD8^+^ T cell response in RCC, we delved into differences in transcriptomes between chemokine^high^ and chemokine^low^ clusters. In gene set variance analysis (34), ssGSEA scores of gene signatures of 26 Reactome pathways were found significantly upregulated in the chemokine^high^ cluster as compared with the chemokine^low^ cluster. These included PD-1 signaling involved in T cell exhaustion and signatures of pathways employing JAK/STAT signaling: IFN (IFN)α/β, IFNγ, interleukin 9 (IL9) and IL21 signaling (**Figure 7**; **Supplementary Table S1**). Of note, the upregulation of JAK/STAT along with an activated chemokine and cytokine signaling, and increased cytotoxicity in the chemokine^high^ cluster were identified by the SPIA algorithm modelling activity of signaling pathways based on differential gene expression (45) (**Supplementary Figure S15**).

To investigate the JAK/STAT signaling and T cell exhaustion process in more details, we resorted to identification of genes differentially expressed in the chemokine^high^ cluster as compared with chemokine^low^ RCCs (**Supplementary Tables S2, S3**). Out of 140 transcripts associated with the IFNα/β and IFNγ Reactome pathways, between 21 and 71 genes were upregulated in chemokine^high^ RCCs of the TCGA KIRC, E-MTAB 1980, GSE73731, GSE167093, RECA-EU and CM 025 collectives. Only two IFN signaling/related genes were differentially regulated in the smallest CM 010 cohort. The top regulated IFN signaling-related genes encompassed IFNγ (*IFNG*), genes of signaling mediators (*JAK2*, *STAT1*, *ISG15*), canonical transcriptional targets of IFN signaling (*IRF* and *GPB* family), anti-viral defense genes (*OAS1*, *OASL*, *SAMHB*), antibody receptors (*FCGR1B*, *TRIM21*) and genes of immunoproteasome and antigen-presenting complex (*PSMB8*, *B2M*, *HLA* family). In particular, high levels of *IFNG* and direct JAK2/STAT1 transcriptional targets suggest high activity and functionality of IFNγ-mediated signaling in chemokine^high^ RCCs (**Supplementary Figures S16, S17**). Of note, *CXCL9/10/11* belong to classical IFNγ-stimulated genes and as such may perpetuate CD8^+^ T cell recruitment to the IFN-rich milieu of chemokine^high^ RCCs.

**Figure 8:**
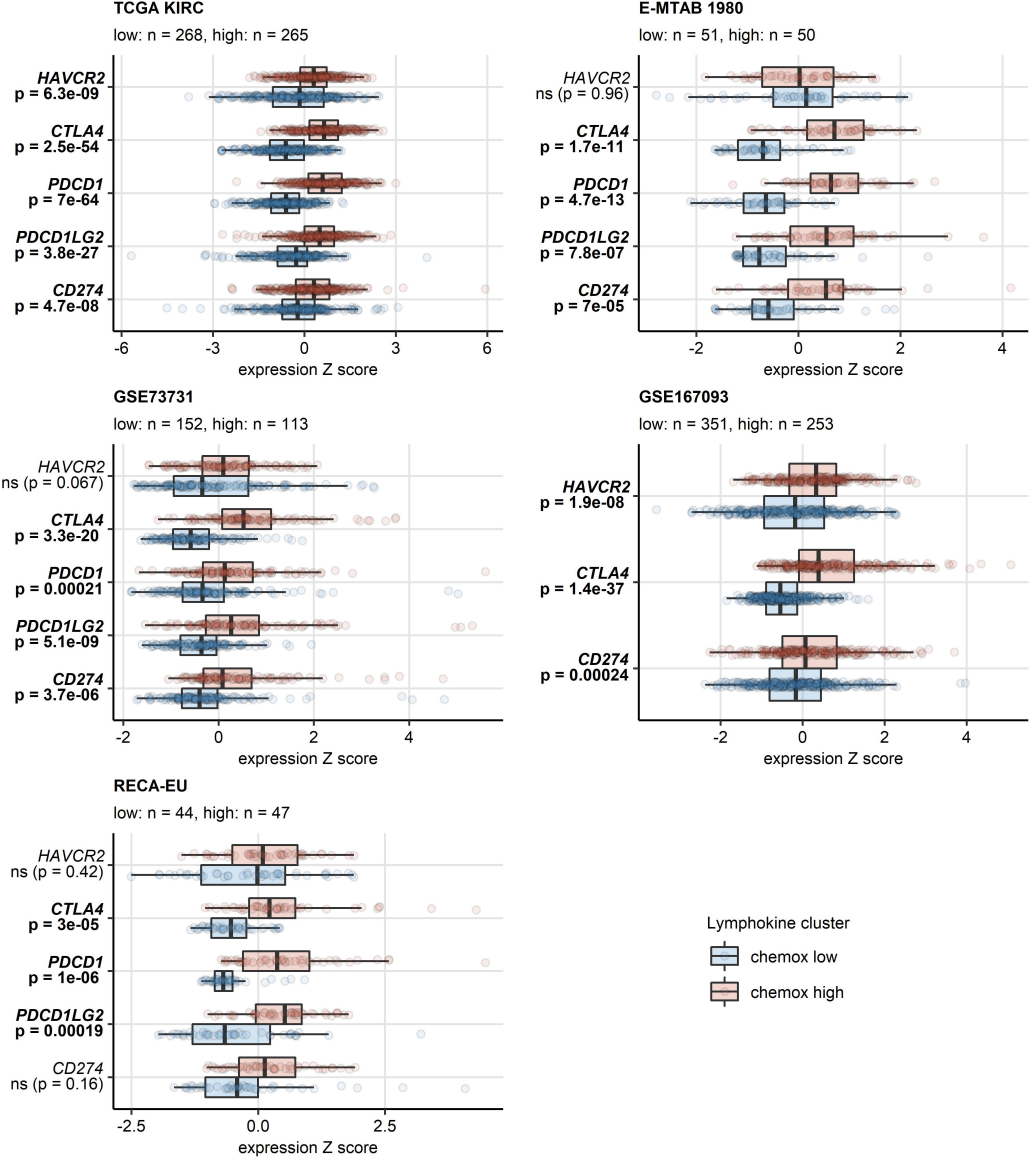
Differential expression of genes coding for clinically relevant immune checkpoint proteins in the two chemokine clusters. Genes differentially expressed in chemokine (chemox)^high^ versus chemokine^low^ RCCs were identified by false discovery rate (FDR) corrected two-tailed T test in the TCGA KIRC, E-MTAB 1980, GSE73731, GSE167093, RECA-EU collectives. Results for clinically relevant immune checkpoint genes are presented. Median normalized log_2_ expression levels with interquartile ranges (IQR) are presented as boxes, whiskers span over the 150% IQR. Points represent single cancer samples. P values are indicated in the Y axes, significant effects are highlighted in bold. Numbers of cancer samples assigned to the chemokine^high^ and chemokine^low^ clusters are displayed in the plot captions.

A sizable fraction of genes related to T cell exhaustion (18–21) was upregulated in the chemokine^high^ cluster as compared with chemokine^low^ cluster of the TCGA KIRC, E-MTAB 1980, GSE73731, GSE167093, RECA-EU and CM 025 collectives (total genes: 87, upregulated genes: 17 - 64). These upregulated genes included transcripts of surface co-stimulation/inhibition molecules (*TIGIT*, *CD27*, *ICOS*, *LAG3*, *TNFSRF9*), cytotoxic proteins (*GZMB*) and transcription factors driving T cell differentiation, persistence and exhaustion (*TOX*, *EOMES*, *BATF*) (**Supplementary Figures S18, S19**). Genes coding for immune checkpoint proteins, *PDCD1*, *CD274* (PD-L1), *PDCD1LG2* (PD-L2), *CTLA4* and *HAVCR2* (TIM3), were found to be expressed at significantly higher levels in the chemokine^high^ than chemokine^low^ cluster (**Figure 8**, **Supplementary Figure S20**). In sum, the abundant CD8^+^ T cell infiltration and high activity of potentially anti-tumorigenic IFN signaling in the chemokine^high^ cluster is strongly counteracted by multiple redundant immune checkpoint and T cell exhaustion processes.

### 3.7 Altered energy metabolism and immunosuppressive IDO1-mediated tryptophan degradation in Chemokine^high^ RCC

**Figure 9:**
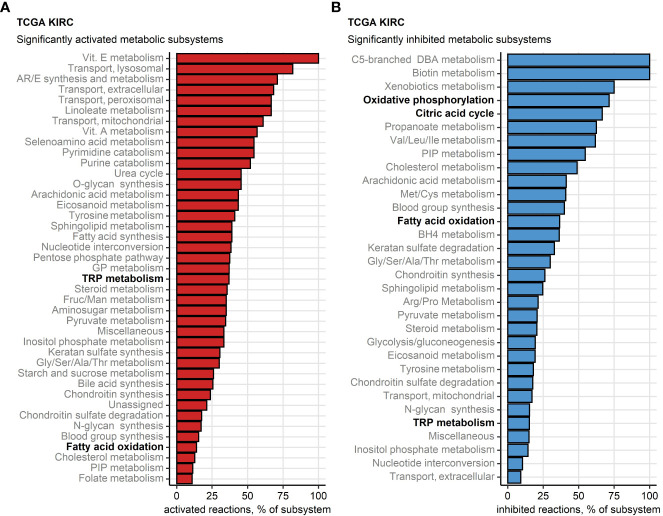
Differential regulation of metabolic reactions between the two chemokine clusters. Regulation of the Recon human metabolism model reactions in chemokine (chemox) high versus chemokine low cluster cancers was predicted based on the differential gene expression with the BiGGR and biggrExtra package tools. Significance of the metabolic reaction regulation was assessed by Monte Carlo simulation and corrected for multiple testing with the false discovery rate (FDR) method. Fractions of significantly activated **(A)** and significantly inhibited **(B)** reactions within the Recon model subsystems are presented for the TCGA KIRC cohort. Fatty acid oxidation, citric acid cycle, oxidative phosphorylation and tryptophan (TRP) metabolism subsystems investigated in more detail are highlighted in bold.

Finally, we sought to investigate potential alterations of cellular metabolism in the chemokine^high^ cluster as compared with chemokine^low^ cluster based on whole-genome differences in gene expression (46, 47) (**Supplementary Table S5**). Substantial fractions of reactions involved in oxidative energy metabolism such as fatty acid oxidation, citric acid cycle (also known as TCA or Krebs cycle) and oxidative phosphorylation (OXPHOS) were found to be inhibited in chemokine^high^ RCCs. In turn, one-third of enzymatic reactions of tryptophan metabolism (TRP) implicated in T cell immunosuppression were significantly upregulated in chemokine^high^ RCCs of the TCGA KIRC collective (**Figure 9**). In more detail, widespread significant inhibition of multiple fatty acid oxidation reactions was observed in chemokine^high^ cancers of the TCGA KIRC, E-MTAB 1980, GSE73731, GSE167093 and CM 025 nivolumab cohorts (**Supplementary Figure S21**). An overall reduced activity of the citric acid cycle was particularly evident in the chemokine^high^ cluster of the four largest collectives (TCGA KIRC, E-MTAB 1980, GSE73731 and GSE167093) and the 2-oxoglutarate - malate section of the cycle (**Supplementary Figure S22**). In OXPHOS, sustained inhibition of the complexes I and IV could be detected (**Supplementary Figure S23**).

**Figure 10:**
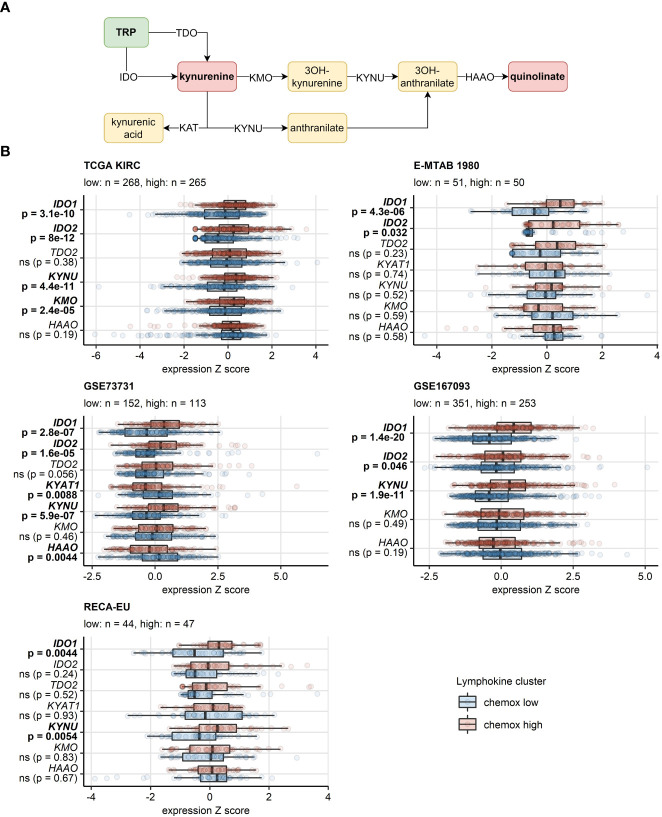
Differential expression of genes involved in the tryptophan/kynurenine/quinolinate metabolism between the two chemokine clusters. Genes differentially expressed in chemokine (chemox)^high^ versus chemokine^low^ RCCs were identified by false discovery rate (FDR) corrected two-tailed T test. Results for genes involved in the tryptophan - kynurenin - quinolinate metabolic pathway are presented. **(A)** Pathway scheme. TRP, tryptophan; TDO, tryptophan 2,3-dioxygenase; KMO, kynurenine 3-monooxygenase; KYNU, kynureninase; KAT, kynurenin aminotransferase; HAAO, 3-hydroxyanthranilate 3,4-dioxygenase. **(B)** Gene expression in the clusters. Median normalized log_2_ expression levels with interquartile ranges (IQR) are presented as boxes, whiskers span over the 150% IQR. Points represent single cancer samples. P values are indicated in the Y axes, significant effects are highlighted in bold. Numbers of cancer samples assigned to the chemokine high and low clusters are displayed in the plot captions. IDO, indoleamine 2,3-dioxygenase; KYAT1, kynurenine—oxoglutarate transaminase 1.

In the chemokine^high^ cluster of the TCGA KIRC, E-MTAB 1980, GSE73731, GSE167093 and RECA-EU cancers, significant increase (>50%) in activity of IDO was detected, a reaction responsible for TRP depletion and the first step of synthesis of immunosuppressive kynurenine and quinolinic acid. In the CM collectives, an increased predicted IDO activity was evident, yet missed statistical significance following multiple testing correction. Furthermore, we could observe an elevated activity of kynureninase (KYNU), a key enzyme in quinolinate biosynthesis, in chemokine^high^ RCCs as compared with the chemokine^low^ cluster (**Supplementary Figure S24**). These findings were corroborated at the gene expression level in the TCGA KIRC, E-MTAB 1980, GSE73731, GSE167093, where both isoforms of IDO, *IDO1* and *IDO2*, were significantly upregulated in the chemokine^high^ cluster. Significantly higher levels of *IDO1* could be detected in the RECA-EU and CM 025 everolimus chemokine^high^ cancers as well. *KYNU* gene was also expressed at significantly increased levels in chemokine^high^ RCCs in four out of eight investigated cohorts (**Figure 10**; **Supplementary Figure S25**).

## 4 Discussion

RCC is considered as an immunogenic tumor with frequent infiltration of immune cells. However, infiltrating anti-tumor immune cells may become dysfunctional in the TME. In addition, regulatory immune cells such as Tregs and MDS cells can infiltrate into the TME resulting in the impairment of tumor immunity (53).

Chemokines play an essential role within the TME by differentially regulating the infiltration of various immune cell subsets (16), guiding the trafficking behavior of T cells (54) and thus, influencing therapeutic outcomes in cancer patients (16). Consequently, targeting chemokine signaling pathways might be an innovative concept to improve efficacy of current cancer therapies including immunotherapy (16). We previously investigated a possible role of different chemokine receptors in RCC showing that CCR3 was predominantly expressed in RCC cells and correlated with a higher tumor grading (55). Moreover, its ligand eotaxin-1 (CCL11), possibly up-regulated as a result of tumor-associated inflammation, might be involved in the development and progression of RCC (55). In addition to CCR3, increased expression of CXCR3 and its ligands in RCC tissue compared to normal kidney tissue have already been reported (56–58). To more systematically examine the role of chemokines and their cognate receptors in RCC we performed a comprehensive analysis of published bulk RNAseq data from a total of 1819 RCC samples and identified, within the set of 28 chemokine genes available for all eight datasets, an overexpression of *CXCL9/10/11/CXCR3, CXCL13/CXCR5 and XCL1/XCR1* in RCC compared with normal renal tissue. Collectively, the results of correlation, scRNAseq and semi-supervised clustering put forward *CXCL9/10/11*, *CXCL13* and *XCL1* chemokine genes along with their receptors *CXCR3*, *CXCR5* and *XCR1* as the key molecules involved in recruitment of effector memory and central memory CD8^+^ T cells to the RCC TME. Focusing on the cellular localization of these chemokines of interest and cognate receptors, M1 TAMs, T cells, NK cells and tumor cells were the major sources of chemokines. In contrast, the cognate receptors CXCR3, CXCR5 and XCR1 were predominantly expressed on T cells (CXCR3), B cells (XCR5) and denritic cells (XCR1).

RCC shows a high frequency of metabolic reprogramming (59–61) and importantly, such local and systemic metabolic alterations can affect anti-tumor immune responses (62). We could previously demonstrate that tumor-infiltrating T cells in RCC are functionally impaired due to mitochondrial and glycolytic dysfunction (48). In accordance, in our current comprehensive analysis we found a significant correlation between the chemokine^high^ cluster and reduced OXPHOS. The entire process from fatty acid oxidation and TCA cycle to the electron transport chain (ETC) was significantly suppressed. Importantly, Complex I and Complex IV of the ETC were most strongly inhibited in the chemokine^high^ RCC cluster. This phenomenon of “nutrient competition exhaustion” by reduced OXPHOS might be explained by a recent study which has shown that CD4^+^ T cells preferentially differentiate towards Tregs when OXPHOS is inhibited (63).

**Figure 11:**
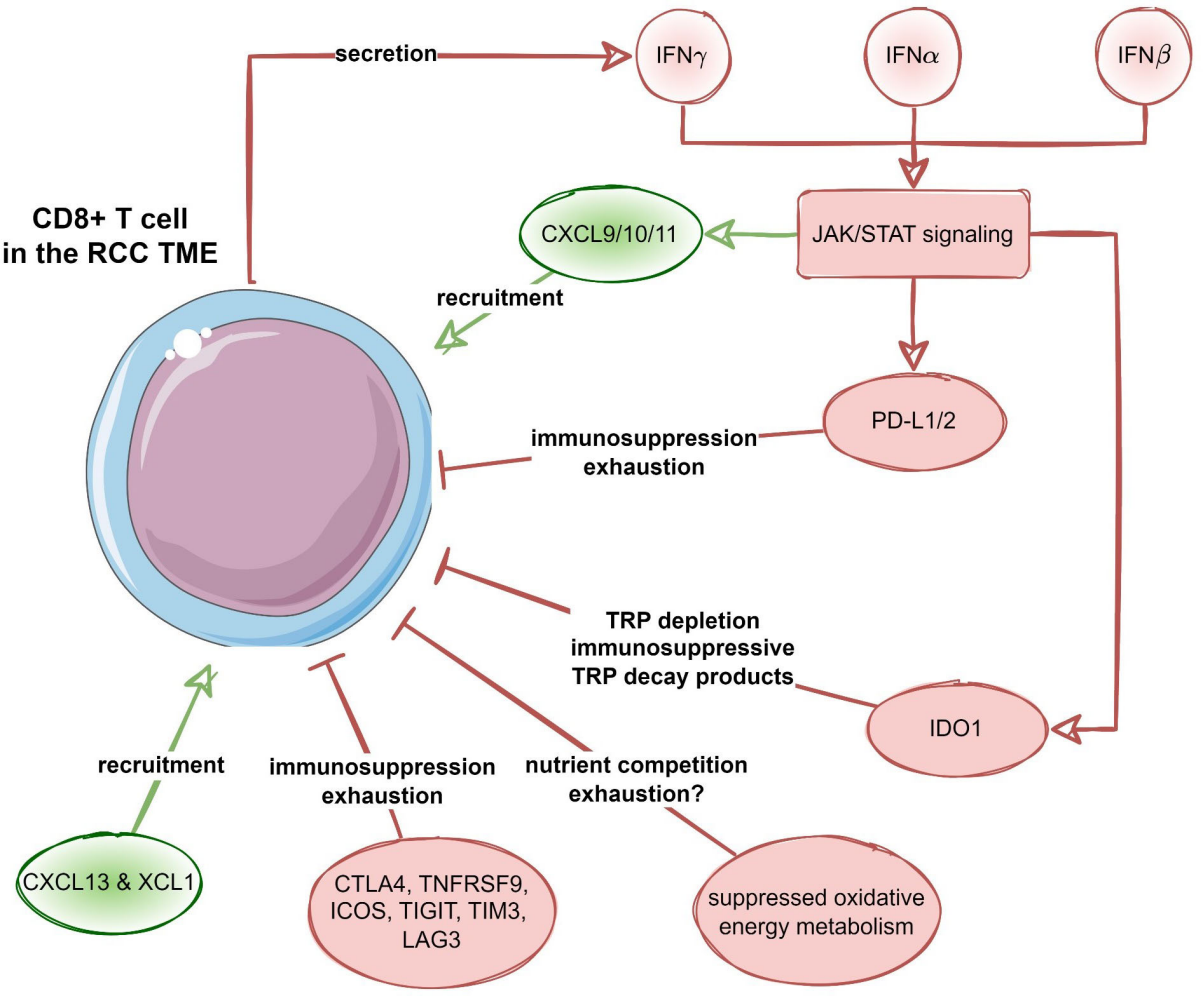
Recruitment and suppression of CD8^+^ T cells in the RCC microenvironment. Proposed negative feedback between the CXCL9/10/11-, CXCL13- and XCL1-mediated CD8^+^ T cell recruitment, interferon (IFN) - JAK/STAT signaling, as well as T cell suppression and exhaustion by immune checkpoint proteins, suppression of oxidative energy metabolism and tryptophan degradation to immunosuppressive metabolites by increased IDO1 activity.

Among transcripts differentially regulated in the chemokine^high^ cluster, multiple STAT-activated genes were found, suggestive of activation of IFN/JAK/STAT signaling. This is of interest as this specific signaling pathway drives for example the expression of CXCL9/10/11 and CXCR3 (64). Moreover, the same pathway promotes IDO-1-mediated degradation of tryptophan resulting in the accumulation of the immunosuppressive metabolite kynurenine (65). We recently described that expression of IDO-1, an enzyme that catalyzes TRP and induces the accumulation of kynurenine metabolites, was predominantly expressed in tumor endothelial cells and was mostly absent from RCC tumor cells (66). Of note, in this current analysis, the TRP degrading pathway was also upregulated in the chemokine^high^ RCC cluster. Moreover, a series of genes related to T cell exhaustion was upregulated in the chemokine^high^ cluster including transcripts of surface co-stimulation/inhibition molecules, cytotoxic proteins and transcription factors driving T cell differentiation, persistence and exhaustion as well as immune checkpoint proteins such as *PDCD1*, *CD274* (PD-L1), *PDCD1LG2* (PD-L2), *CTLA4* and *HAVCR2* (TIM3). Thus, results of metabolic reaction activity modeling suggest profound inhibition of OXPHOS in the chemokine^high^ RCC cluster along with an increased activity of the immunosuppressive IDO1-mediated degradation pathway. These processes may pose another hurdle to effective anti-tumor T cell response in RCCs hallmarked by high expression of the T cell-attracting chemokines *CXCL9/10/11*, *CXCL13* and *XCL1*. In sum, the T cell-inflamed TME in RCC is characterized by CD8^+^ T cell infiltration, which is mediated by these specific chemokines, and an IFNγ signature, indicating a strong interplay between tumor cells and immune cells. However, CD8^+^ T cell-inflamed RCCs may activate different immunosuppressive redundant immune checkpoint and T cell exhaustion pathways such as IDO1 and/or PD-L1/2, CTLA4, TIGIT, LAG3 and TIM3 (**Figure 11**). Thus, upregulation of immunosuppressive pathways within the TME is more intrinsically driven by immune cells itself rather than by tumor cells (67).

Focusing on the predictive and prognostic role of our identified chemokine signature, we could neither observe significant differences in survival nor in response to immunotherapy between the chemokine^high^ and chemokine^low^ RCC cluster. None of the chemokine genes used for cluster definition correlated consistently and significantly with OS or RFS. This fact may suggest, that potential beneficial effects of CD8^+^ T cells recruited *via CXCL9/10/11*, *CXCL13* and *XCL1* are likely outweighed by potent activated immunosuppressive properties of the RCC TME as described here. An additional explanation relates to the fact that chemokines may differentially regulate the biological function of T cells, resulting in distinct anti- and pro-tumoral effects in the TME (64, 68). While *CXCL9/10/11* may exhibit indirect anti-tumor effects through the recruitment of CXCR3-expressing T cells, these chemokines and their cognate receptor may also contribute to tumor development and metastasis *via* T cell-independent mechanisms (56). Activation of CXCR3 on different tumor cell types has been shown to prevent apoptosis and promote proliferation. Moreover, immunohistochemical detection of CXCR3 on localized RCC correlated with poor disease prognosis (69). In addition to promoting and sustaining tumor development, CXCR3 may facilitate tumor cell dissemination, for instance, to the lymph nodes (64, 68). Thus, when RCC acquires CXCR3 expression and possibly also CXCL9 expression, this fact can generate an autocrine CXCL9/CXCR3 axis that supports tumor progression and metastasis. CXCL13, originally identified as a B cell chemoattractant, and its receptor CXCR5 have also emerged as key players of carcinogenesis and cancer progression (70). They can also act *via* autocrine and paracrine signals between the TME and the tumor cells itself (70). In RCC, CXCL13 promotes tumor cell proliferation and migration by activation of PI3K/Akt/mTOR signaling. Thus, CXCL13 up-regulation has been shown to correlate with advanced disease stage and poor prognosis (71). In addition, XCL1 positively correlated with the expression of several inhibitory receptors and cytokines that are associated with T cell exhaustion and several immune checkpoints in ccRCC, thus resulting in a significantly poorer survival (72). However, as evident from a comparison of concordance indexes of the multi-parameter model consisting of solely clinical and demographic variables and the model including gene expression parameters, there was no add-on value of the investigated chemokine gene expression levels to the prediction accuracy of OS or response to immunotherapy. Despite the fact that chemokines included in our chemokine^high^ cluster seem to be good candidates to predict response to immunotherapy (73, 74), RCCs with chemokine^high^ expression did not show better survival or response to immunotherapy in the present study. One reason we show for this is an increased activation of immunosuppressive pathways such as IDO, which is known to lead to resistance to immunotherapy (75). In RCC, we also showed in a previous work that IDO1 expression correlates positively with increased CD8^+^ T cells reflecting a T cell-inflamed TME in RCC (66). IDO1 is responsible for immune escape mechanisms for tumor cells contributing to T cell exhaustion (76). As a consequence, accelerated breakdown of TRP with increased IDO-1 activity is associated with disease progression, decreased OS and poor prognosis in different cancer entities (77). Thus, blocking more than one immunosuppressive pathway combining PD-1/PD-L1 inhibitors with IDO1 inhibitors may improve the therapeutic response to immunotherapy especially in chemokine^high^ RCCs. Early data already show a promising response of the combination pembrolizumab and the IDO inhibitor epacadostat in metastatic RCC (78). The corresponding phase 3 study (KEYNOTE-679/ECHO-302) is currently ongoing.

There are some limitations of our research. The analysis of published bulk RNAseq data does not allow us to link *CXCL9/10/11, CXCL13 and XCL1* expression with T cell priming, differentiation and exhaustion. Analogically, it does not explain how *CXCL9/10/11, CXCL13 and XCL1* overexpression induces reprogramming of energy metabolism and increased IDO-mediated tryptophan degradation. To address that and validate our bioinformatic analysis results, ex vivo assays and protein level analyses including both chemokine^high^ and chemokine^low^ RCC are considered as a next research step.

## 5 Conclusion

*XCL9/10/11/CXCR3, CXCL13/CXCR5 and XCL1/XCR1* expression defines a chemokine^high^ subset of RCC characterized by abundant CD8+ T cell infiltration and active IFN/JAK/STAT signaling. These favorable prognostic features are overweighed by redundant immunosuppression processes involving T cell exhaustion, suppression of oxidative energy metabolism and OXPHOS, and IDO1-mediated tryptophan degradation. As a result, no survival or therapy response benefit could be observed for chemokine^high^ RCCs. Our results stress the importance of therapeutic combinations targeting multiple immunosuppressive pathways and metabolic reprogramming in RCC.

## Data availability statement

The datasets presented in this study can be found in online repositories. The names of the repository/repositories and accession number(s) can be found in the article/**Supplementary Material**.

## Ethics statement

No ethics committee approval was needed for publicly available anonymous expression and clinical information data sets. Donors of tissue for flow cytometry measurements provided written consent according to protocols approved by the ethical committee of the University Hospital Regensburg (Nr. 08/108) in accordance with the Declaration of Helsinki.

## Author contributions

RP was responsible for conceptualization, data curation, formal analysis, investigation, visualization, methodology, writing – original draft, project administration, writing – review and editing. PT performed data curation, software, formal analysis, methodology, project administration, writing – review and editing. PS was responsible for conceptualization, data curation, formal analysis, investigation, visualization, methodology, writing – original draft, project administration, writing – review and editing. AM and GU performed scRNAseq analyses, data curation, software, formal analysis, methodology. RM and FW were responsible for investigation, data curation, formal analysis and methodology. AS and FK performed formal analysis, writing – review and editing. DB and MP were responsible for investigation, formal analysis, writing – review and editing. MT was responsible for supervision, investigation, formal analysis, methodology, writing – original draft, writing – review and editing. Moreover, RP and PS conceived the project. PS designed the experiments. PS performed the experiments (FACS). FW and RM were responsible for material curation and preparation (pathological analysis and FACS, surgically resected RCCs). RP wrote the manuscript with the help of all authors (especially with MT). All authors contributed to the article and approved the submitted version.
