# Supplementary material for: A chemokine network of T cell exhaustion and metabolic reprogramming in renal cell carcinoma
Source: Front Immunol. 2023 Mar 16;14:1095195. doi: 10.3389/fimmu.2023.1095195 (PMC10060976; doi:10.3389/fimmu.2023.1095195)
Supplement: Supplementary file 1 [file DataSheet_1.pdf]

Supplementary Table S1: Reactome signatures differentially regulated between the chemokine clusters (chemokine^{high} vs. chemokine^{low}). The table is available as a supplementary Excel file.

Supplementary Table S2: Numbers of genes differentially regulated between the chemokine clusters (chemokine^{high} vs. chemokine^{low}). The table is available as a supplementary Excel file.

Cohort	N downregulated	N upregulated
TCGA KIRC	539	2,497
E-MTAB 1980	96	965
GSE73731	650	876
GSE167093	45	241
RECA-EU	67	1,788
CM 010	2	33
CM 025 EVER	45	502
CM 025 NIVO	42	359

Supplementary Table S3: Genes differentially regulated between the chemokine clusters (chemokine^{high} vs. chemokine^{low}). The table is available as a supplementary Excel file.

Supplementary Table S4: Genes differentially regulated between the chemokine clusters (chemokine^{high} vs. chemokine^{low}). The table is available as a supplementary Excel file.

Supplementary Table S5: Recon model metabolic reactions predicted to be significantly modulated between the chemokine clusters (chemokine^{high} vs. chemokine^{low}). The table is available as a supplementary Excel file.

Supplementary Table S6: Descriptive characteristics of tissue donors for flow cytometry analysis.

Variable	Patient 1	Patient 2	Patient 3	Patient 4
Age, years	61	55	81	77
Sex	male	male	male	male
Tumor grade	2	1	2	1
Tumor stage	T1	T3	T1	T1
Lymph node stage	N0	N0	N0	N0
Previous treatment	no	no	no	no
Tumor entity	ccRCC	ccRCC	ccRCC	ccRCC
Peripheral leukocytes, cells/nL	6.38	9.24	6.34	4.24

Supplementary Figure S1: **Screening for tumor-expressed chemokines involved in CD8⁺ T cell recruitment in the TCGA KIRC cohort.**

(A) Differences in log2-transformed expression of chemokine genes between the RCC and normal kidney tissue investigated in donor-matched tissue specimens by paired two-tailed T test. Expression regulation false discovery rate (FDR) corrected significance and foldregulation estimates in RCC as compared with the normal kidney are presented in a volcano plot. The strongest regulated genes are labeled. The chemokine genes of interest investigated in more detail in the current report are highlighted in bold. The number of RCC – normal kidney pairs are presented next to the plot.

(B) Correlation of log2-transformed chemokine gene expression with CD8+ T cell levels in the RCC tissue predicted by the QuanTIseq algorithm was analyzed by FDR-corrected Spearman test. ρ correlation coefficients with 95% confidence intervals (CI) are shown in a Forest plot. The chemokine genes of interest investigated in more detail in the current report are highlighted in bold. The observation number is indicated in the plot caption.

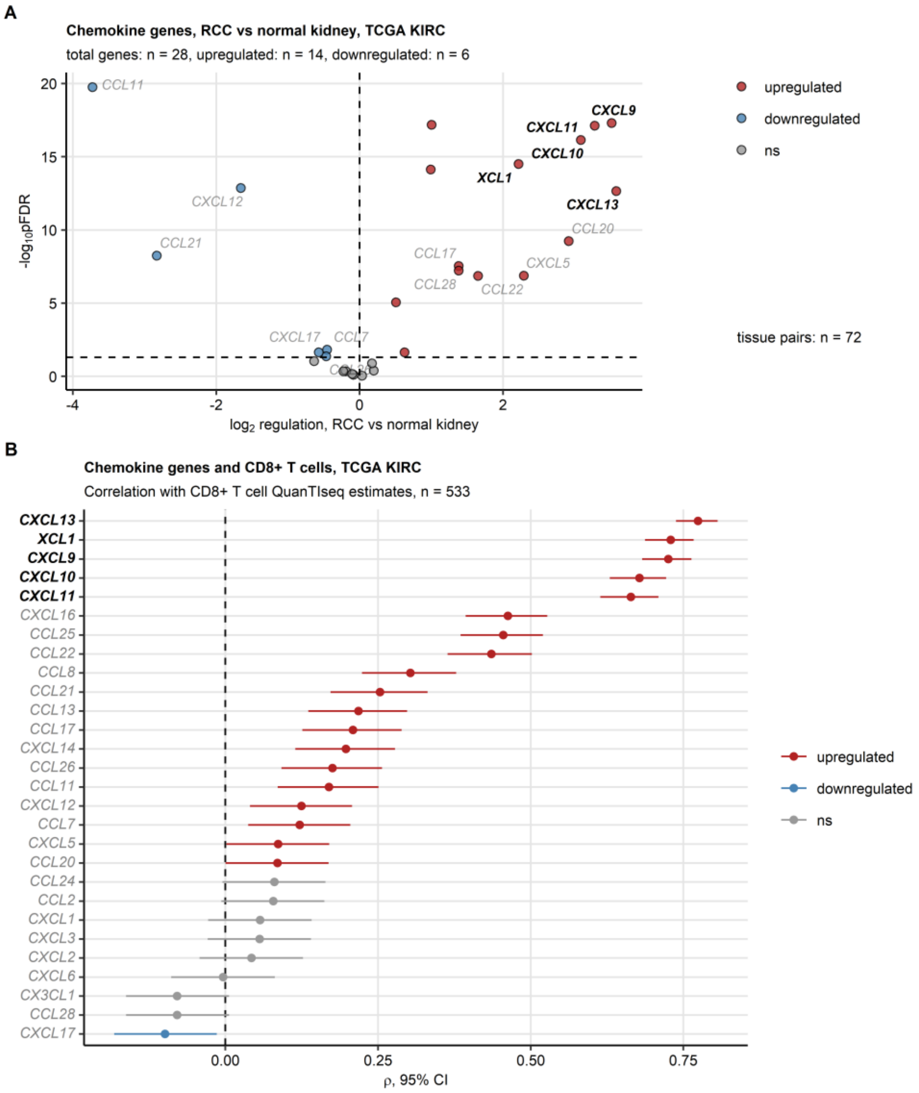

Supplementary Figure S2: Definition of the chemokine clusters in the TCGA KIRC cohort. Chemokine clusters of RCC samples were defined in the TCGA KIRC training cohort in respect to normalized log2-transformed expression of CXCL9/10/11, CXCR3, CXCL13, XCL1 and XCR1 using the PAM algorithm with cosine distance between the cancer samples. The total number of cancer samples is indicated in (A).

(A) Comparison of performance of various clustering algorithms (PAM: partition around medoids; HCL: hierarchical clustering; KMEANS, k = 2 clusters specified for each procedure) and distance metrics (Euclidean, Manhattan and cosine). Clustering structure reproducibility was measured by accuracy of cluster assignment in 10-fold cross-validation. Explanatory performance was measured by fraction of explained clustering variance (ratio of betweencluster sum of squares to total sum of squares). Note superior reproducibility and good explanatory performance of the PAM/cosine distance algorithm.

(B) Choice of the cluster number for the PAM/cosine distance algorithm based on the bend of the within-cluster sum of square curve (WSS curve, left) and the peak mean silhouette statistic (right).

(C) Separation between the chemokine clusters defined in the TCGA KIRC cohort by the PAM/cosine distance algorithm investigated by a heat map of distances between the cancer sample pairs. Numbers of observations assigned to the chemokine (chemox) high and chemokine low clusters are indicated next to the heat map.

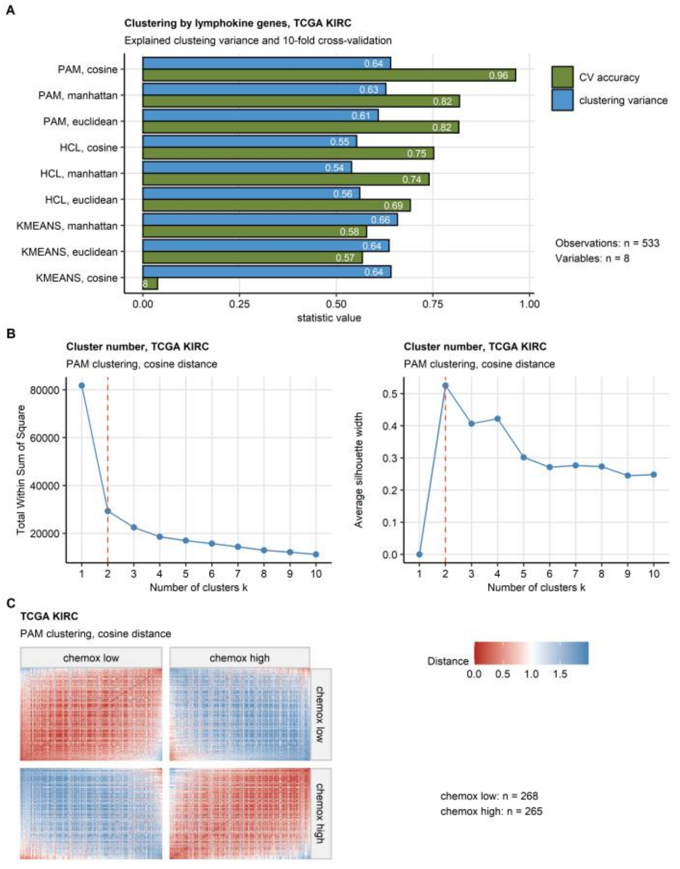

Supplementary Figure S3: Semi-supervised clustering of cancer samples by the chemokine gene expression. Expression of CXCL9/10/11, CXCL13, XCL1 and their cognate receptors in the chemokine clusters, CheckMate cohorts. Chemokine clusters of RCC samples were defined in the TCGA KIRC training cohort in respect to normalized log2-transformed expression of CXCL9/10/11, CXCR3, CXCL13, XCL1 and XCR1 using the PAM algorithm with cosine distance between the cancer samples. Prediction of cluster assignment in the E-MTAB 1980, GSE73731, GSE167093, RECA-EU, CheckMate 010 (CM 010), CheckMate 025 everolimus (CM 025 EVER) and CheckMate 025 nivolumab (CM 025 NIVO) was done with the inverse distance-weighted 15-nearest neighbor (15-NN) classifier.

(A) Explanatory performance of the clustering assignment expressed as fraction of explained clustering variance (ratio of between-cluster sum of squares to total sum of squares, left) and fraction of the cohort's cancer samples assigned to the chemokine (chemox) low and high clusters (right). Numbers of cancer samples are indicated in the Y axes.

(B) Normalized log2 transformed expression of the clustering genes in the chemokine clusters of the CheckMate cohorts. Differences in expression were assessed by false discovery rate (FDR) corrected two-tailed T test. Mean normalized expression values are visualized as thick lines, tinted regions represent two standard errors of the mean (SEM). P values are shown in the Y axis. Numbers of cancer samples assigned to the clusters are displayed in the plot captions.

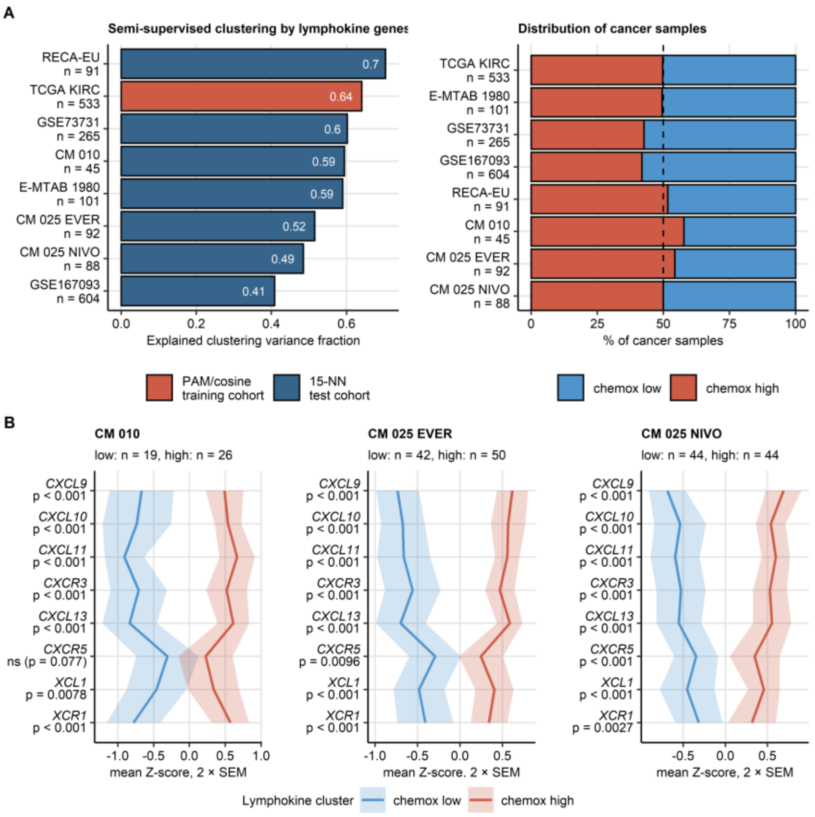

Supplementary Figure S4: Method and sample flowchart of scRNA dataset (48). For single-cell analysis, 'tumor' and 'normal kidney' were extracted from the dataset.

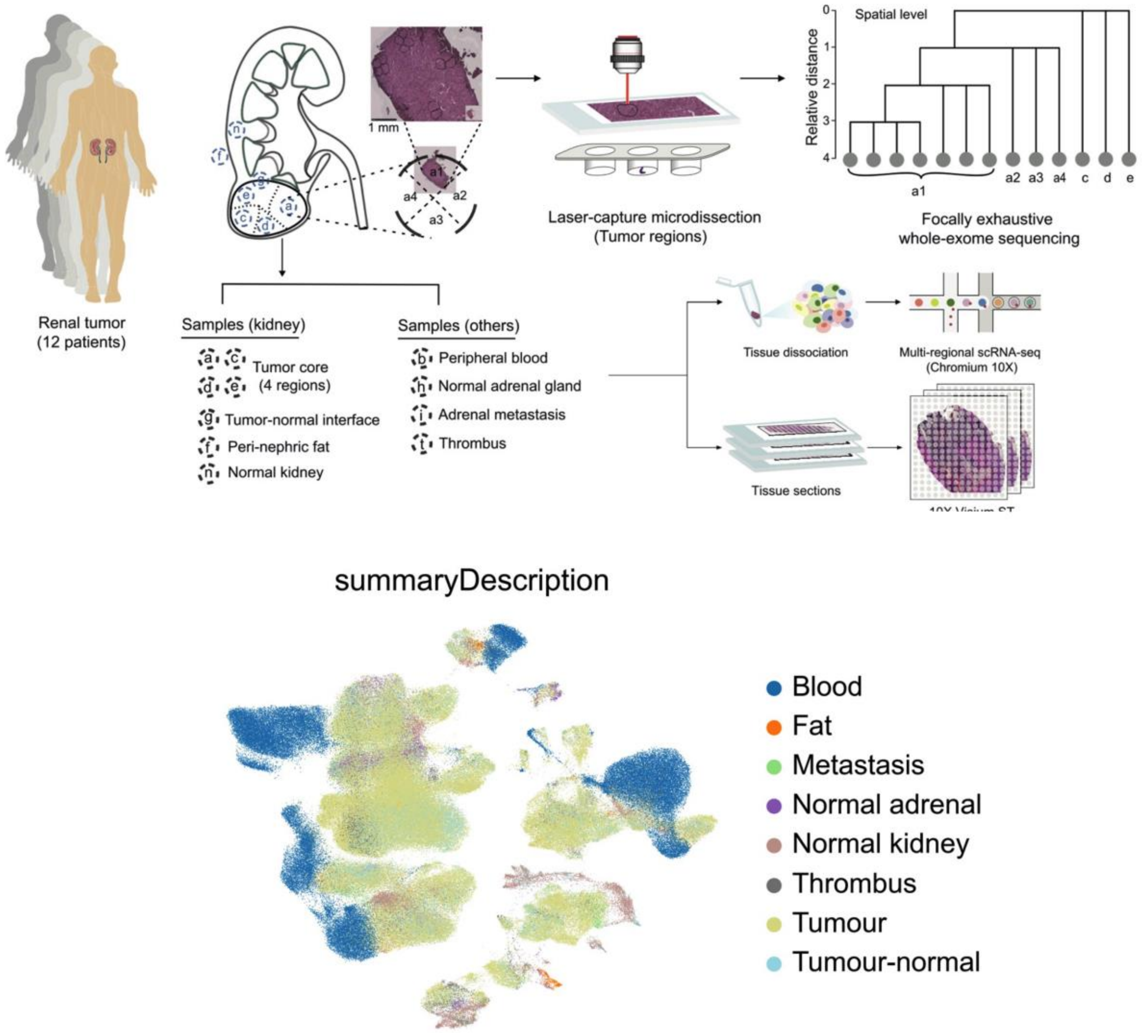

Supplementary Figure S5: Expression of CXCL9/10/11, CXCL13, XCL1 and their cognate receptors in RCC samples enriched in CD8+ and CD4+ T cells, activated myeloid DC, TAM and B cells, CheckMate cohorts. Pairwise association of the gene expression and xCell non-immune cell infiltration estimates was assessed by scaled Spearman's correlation. The genes of interest and the cell types associated with the genes of interest with moderate-to-large strength (correlation coefficient $\rho > 0.4$) were visualized as network force-directed plots with edge weighting by the scaled Spearman's correlation coefficient. CD8+ T: CD8+ T cells, CD4+ T: CD4+ T cells, n: naive; m: memory; em: effector memory; cm: central memory; Th1: T helper cells 1; Th1: T helper cells 1; mDC: myeloid dendritic cells, act mDC: activated mDC; pDC: plasmacytoid dendritic cells; TAM: tumor-associated macrophages; B: B cells, switch Bm: class-switched memory B cells; NK: natural killer cells; NKT: natural killer T cells; T γ δ: γ δ T cells; Mono: monocytes; Eosino: eosinophils.

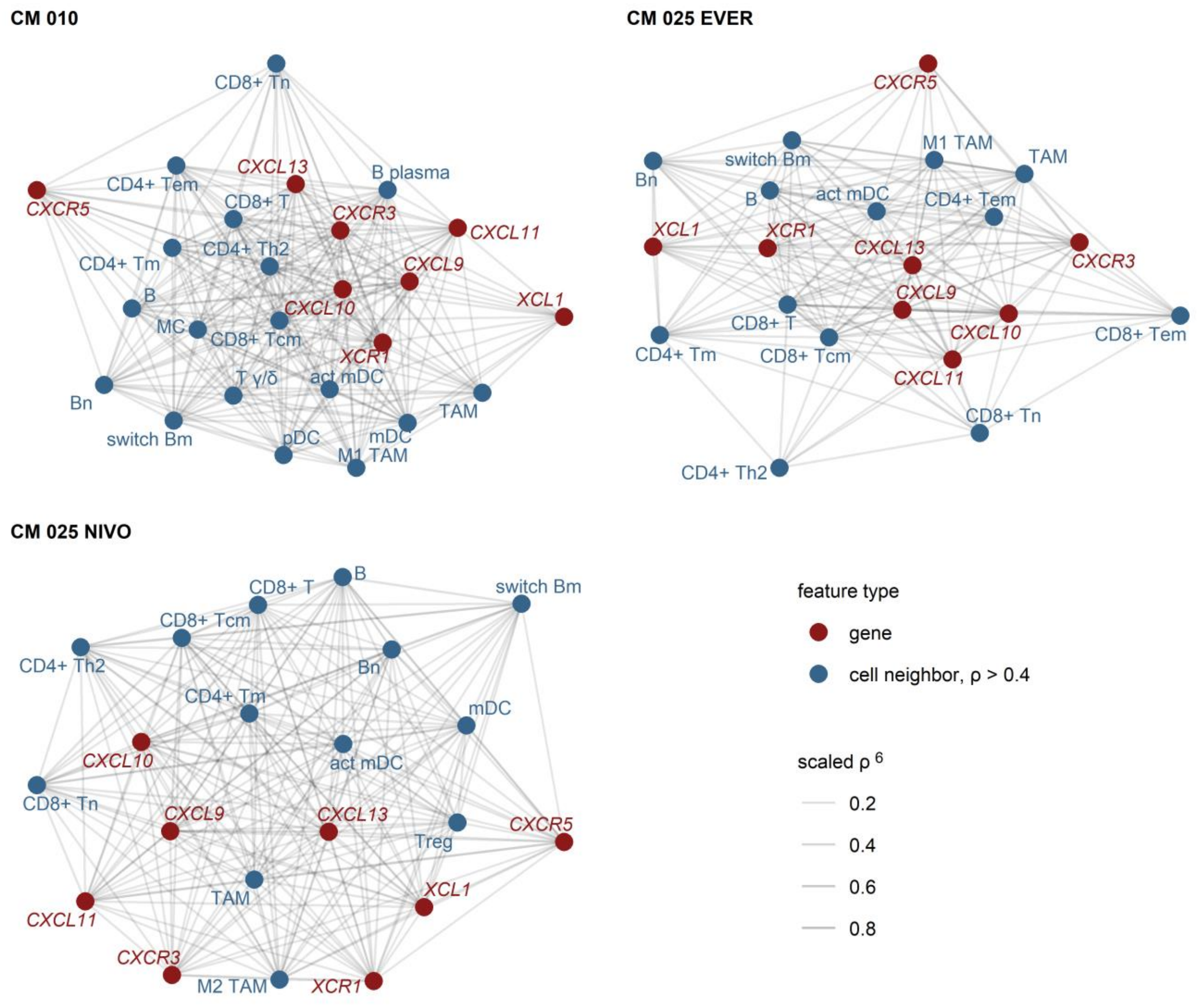

Supplementary Figure S6: Correlation of expression of CXCL9/10/11, CXCL13, XCL1 and their cognate receptors with non-malignant cell population content predicted by the xCell algorithm in the TCGA KIRC, E-MTAB 1980, GSE73731 and GSE167093 cohorts. Correlation of log2-transformed gene expression with non-malignant cell levels in the RCC tissue predicted by the xCell algorithm was analyzed by FDR-corrected Spearman test. ρ correlation coefficients for significant correlations are presented as points in bubble plots. Point size corresponds to the absolute value of ρ, point color cores for the ρ value. Numbers of complete observations are indicated in the plot captions. T: T cells; n: naive; m: memory; em: effector memory; cm: central memory; Th1: T helper 1; Th2: T helper 2; Treg: regulatory T cells; NKT: natural killer T cells; NK: natural killer cells; B: B cells; switch Bm: class-switched memory B cells; mDC: myeloid dendritic cells; act mDC: activated mDC; pDC: plasmacytoid dendritic cells; Neutro: neutrophils; Eosino: eosinophils; MC: mast cells; Mono: monocytes; TAM: tumor-associated macrophages; EC: endothelial cells; CAF: cancer-associated fibroblasts.

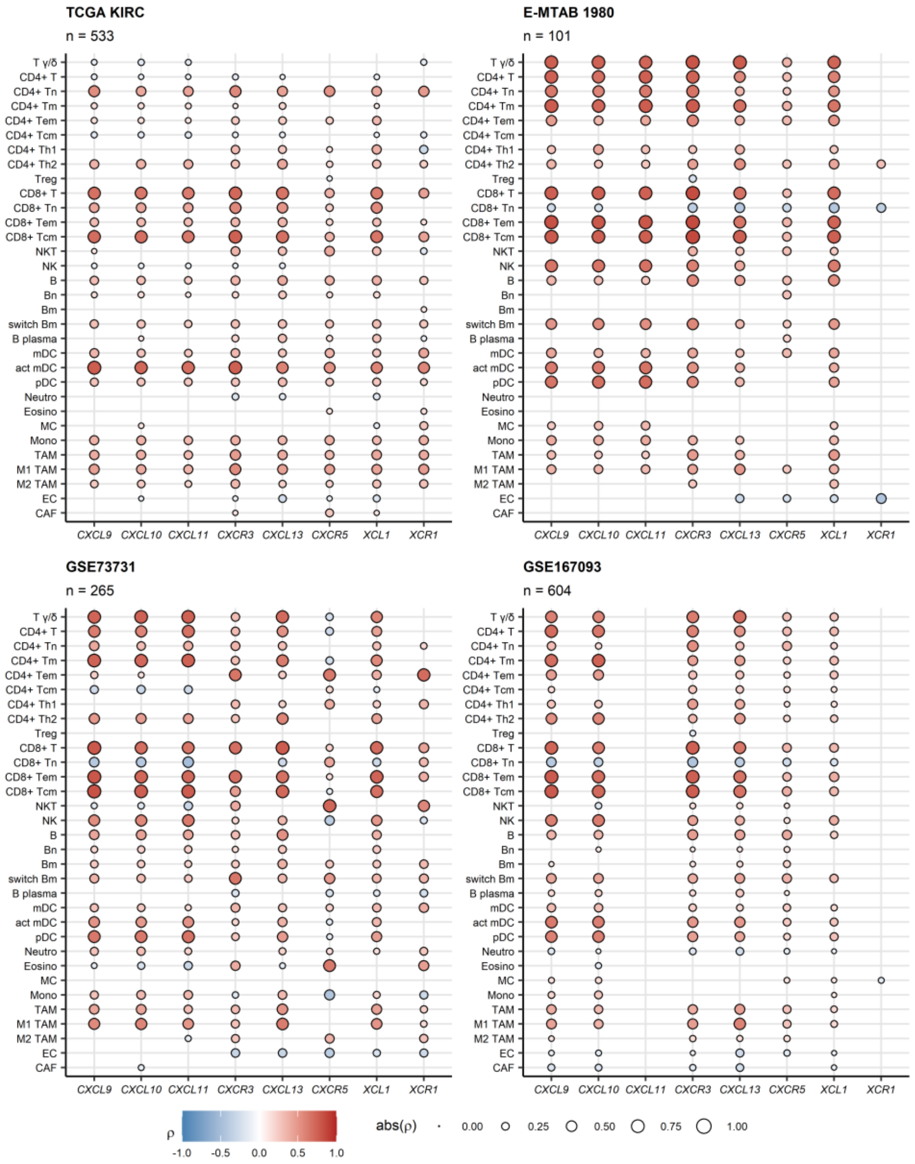

Supplementary Figure S7: Correlation of expression of CXCL9/10/11, CXCL13, XCL1 and their cognate receptors with non-malingant cell population content predicted by the xCell algorithm in the RECA-EU, CheckMate010 and CheckMate025 cohorts. Correlation of log2-transformed gene expression with non-malignant cell levels in the RCC tissue predicted by the xCell algorithm was analyzed by FDR-corrected Spearman test. ρ correlation coefficients for significant correlations are presented as points in bubble plots. Point size corresponds to the absolute value of ρ, point color cores for the ρ value. Numbers of complete observations are indicated in the plot captions. T: T cells; n: naive; m: memory; em: effector memory; cm: central memory; Th1: T helper 1; Th2: T helper 2; Treg: regulatory T cells; NKT: natural killer T cells; NK: natural killer cells; B: B cells; switch Bm: class-switched memory B cells; mDC: myeloid dendritic cells; act mDC: activated mDC; pDC: plasmacytoid dendritic cells; Neutro: neutrophils; Eosino: eosinophils; MC: mast cells; Mono: monocytes; TAM: tumor-associated macrophages; EC: endothelial cells; CAF: cancer-associated fibroblasts; CM 010: CheckMate 010; CM 025 EVER: CheckMate 025 everolimus; CM 025 NIVO: CheckMate 025 nivolumab.

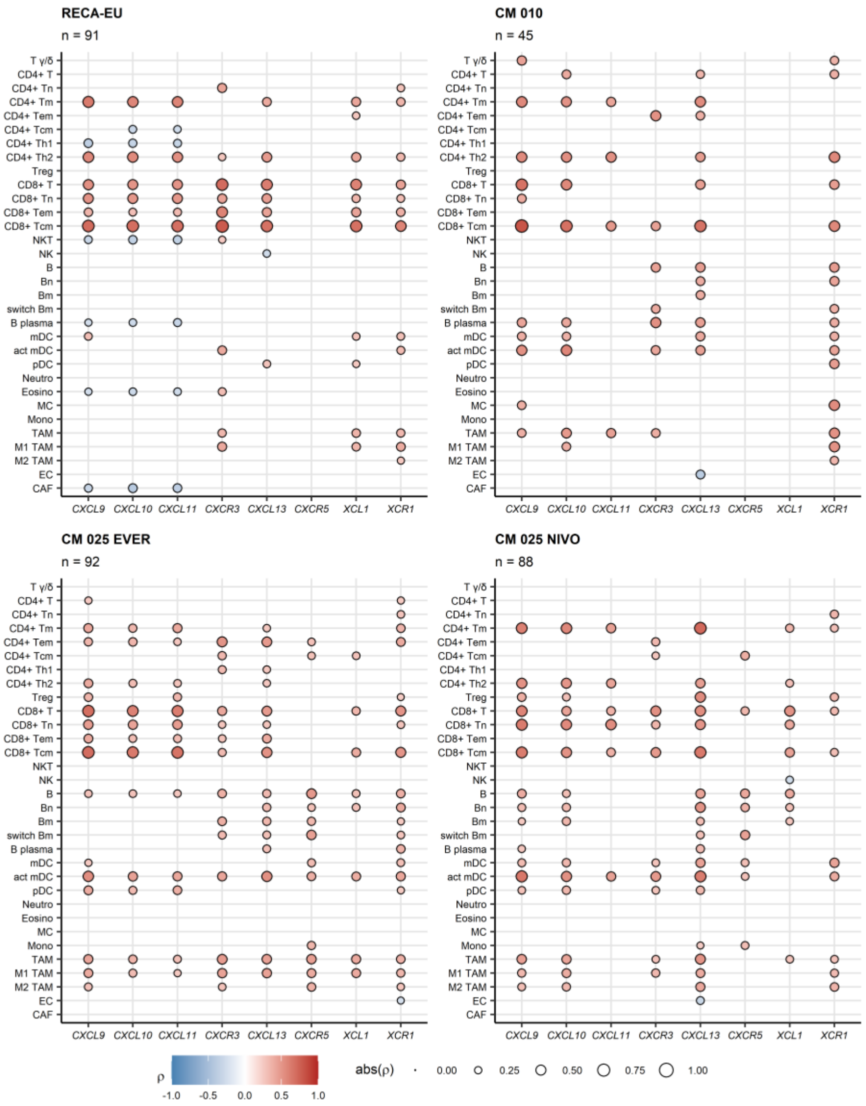

Supplementary Figure S8: Correlation of expression of CXCL9/10/11, CXCL13, XCL1 and their cognate receptors with immune cell population content predicted by the QuanTIseq algorithm in the TCGA KIRC, E-MTAB 1980, GSE73731 and GSE167093 cohorts. Correlation of log2-transformed gene expression with immune cell levels in the RCC tissue predicted by the QuanTIseq algorithm was analyzed by FDR-corrected Spearman test. ρ correlation coefficients for significant correlations are presented as points in bubble plots. Point size corresponds to the absolute value of ρ, point color cores for the ρ value. Numbers of complete observations are indicated in the plot captions. T: T cells; Treg: regulatory T cells; NK: natural killer cells; B: B cells; mDC: myeloid dendritic cells; Neutro: neutrophils; Mono: monocytes; TAM: tumor-associated macrophages; rest: remainder uncharacterized cells.

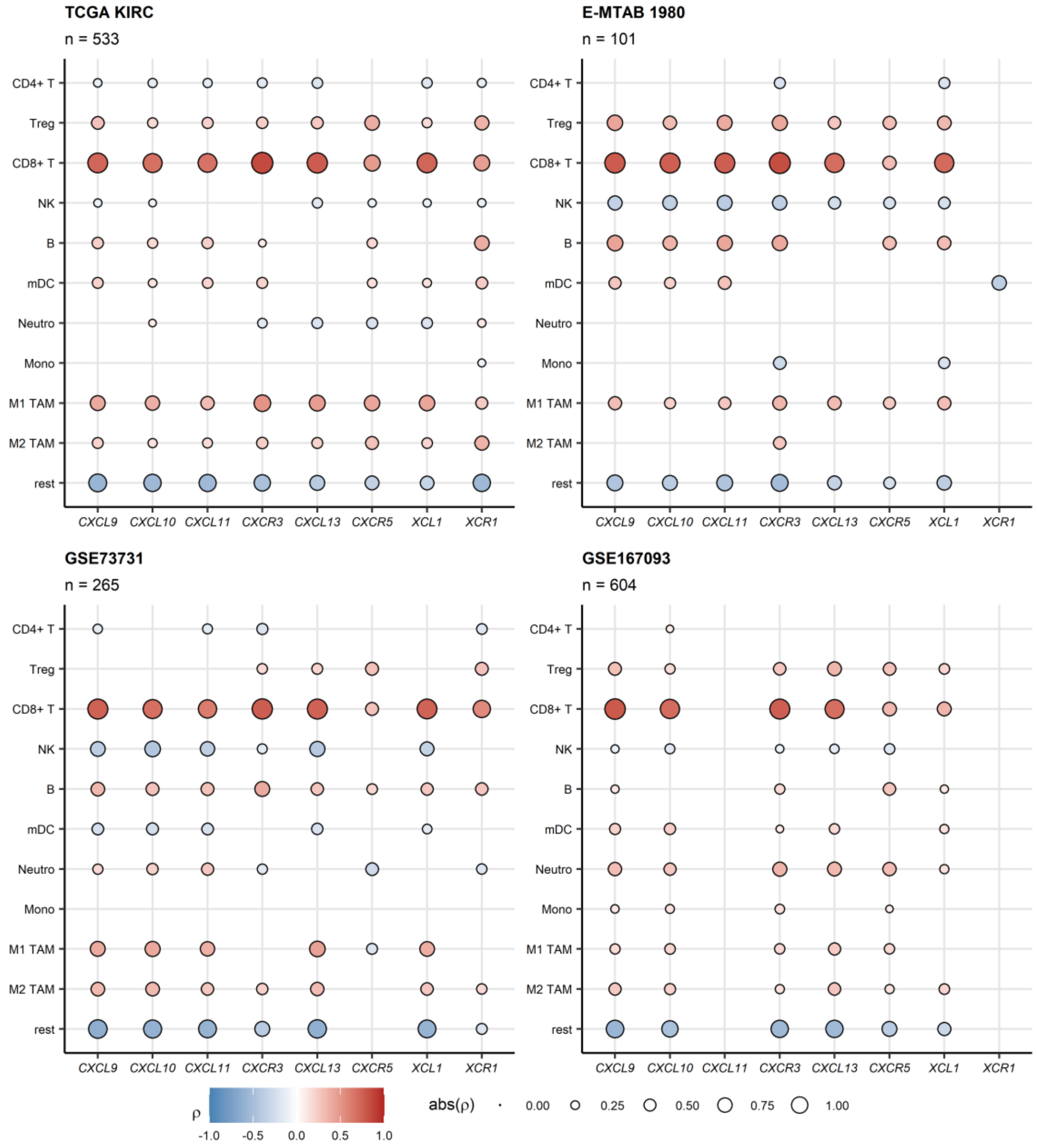

Supplementary Figure S9: Correlation of expression of CXCL9/10/11, CXCL13, XCL1 and their cognate receptors with immune cell population content predicted by the QuanTIseq algorithm in the RECA-EU, CheckMate010 and CheckMate025 cohorts. Correlation of log2-transformed gene expression with immune cell levels in the RCC tissue predicted by the QuanTIseq algorithm was analyzed by FDR-corrected Spearman test. ρ correlation coefficients for significant correlations are presented as points in bubble plots. Point size corresponds to the absolute value of ρ, point color cores for the ρ value. Numbers of complete observations are indicated in the plot captions. T: T cells; Treg: regulatory T cells; NK: natural killer cells; B: B cells; mDC: myeloid dendritic cells; Neutro: neutrophils; Mono:monocytes; TAM: tumor-associated macrophages; rest: remainder uncharacterized cells; CM 010: CheckMate 010; CM 025 EVER: CheckMate 025 everolimus; CM 025 NIVO: CheckMate 025 nivolumab.

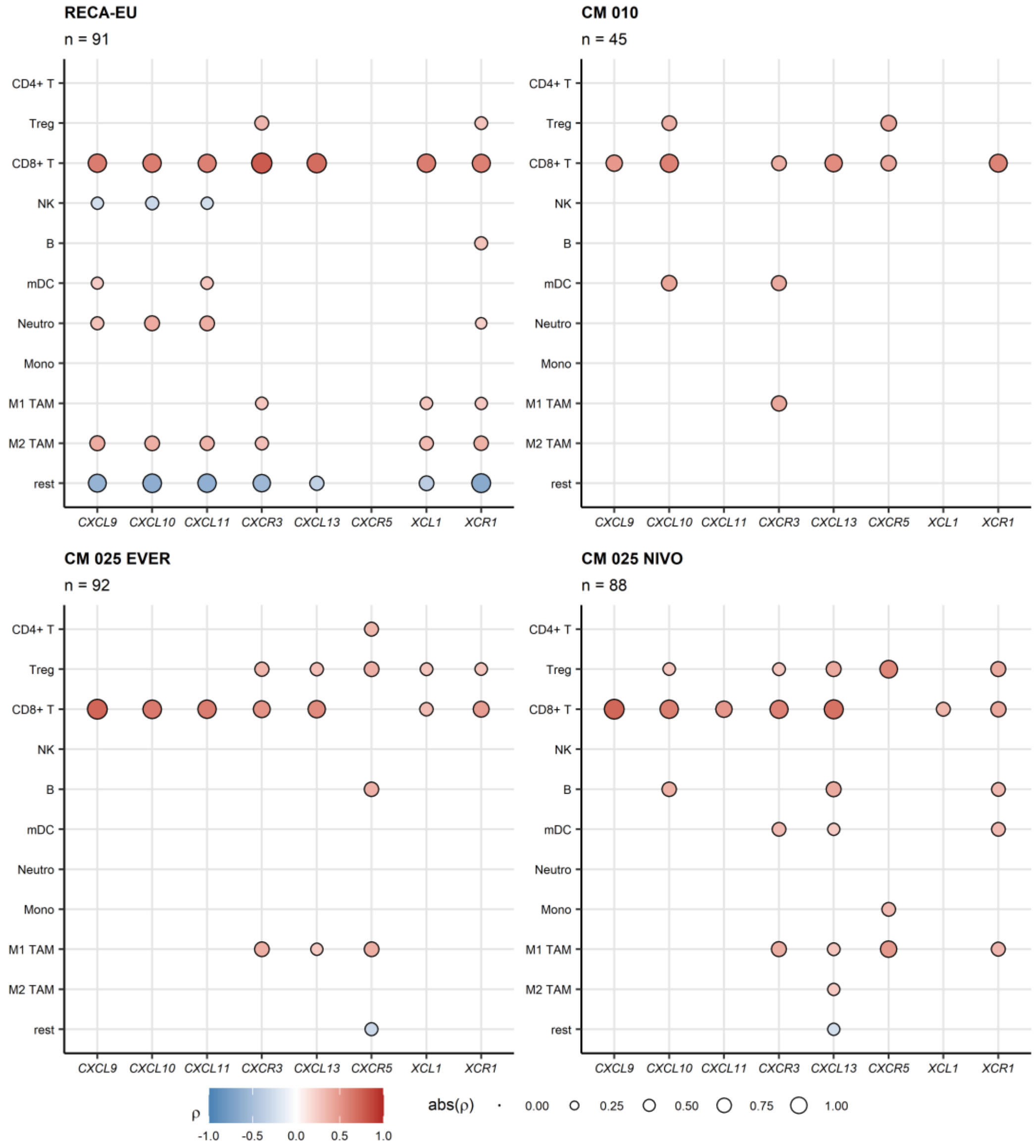

Supplementary Figure S10: Predicted infiltration of T and B cells, activated mDC and TAM in the chemokine clusters, CheckMate cohorts. Differences in levels of effector memory CD8+ T (CD8+ Tem), central memory CD8+ T (CD8+ Tcm), memory CD4+ T (CD4+ Tm), effector memory CD4+ T cells (CD4+ Tem), B cells (B), activated myeloid dendritic cells (act mDC), M1 and M2 tumor-associated macrophages (TAM) between the chemokine (chemox) high and chemokine low cluster were assessed by false discovery rate (FDR) corrected Mann-Whitney test. Median normalized infiltration levels with interquartile ranges (IQR) are presented as boxes, whiskers span over the 150% IQR. Points represent single cancer samples. P values are indicated in the Y axes, significant effects are highlighted in bold. Numbers of cancer samples assigned to the chemokine high and low clusters are displayed in the plot captions. CM 010: CheckMate 010; CM 025 EVER: CheckMate 025 everolimus; CM 025 NIVO: CheckMate 025 nivolumab.

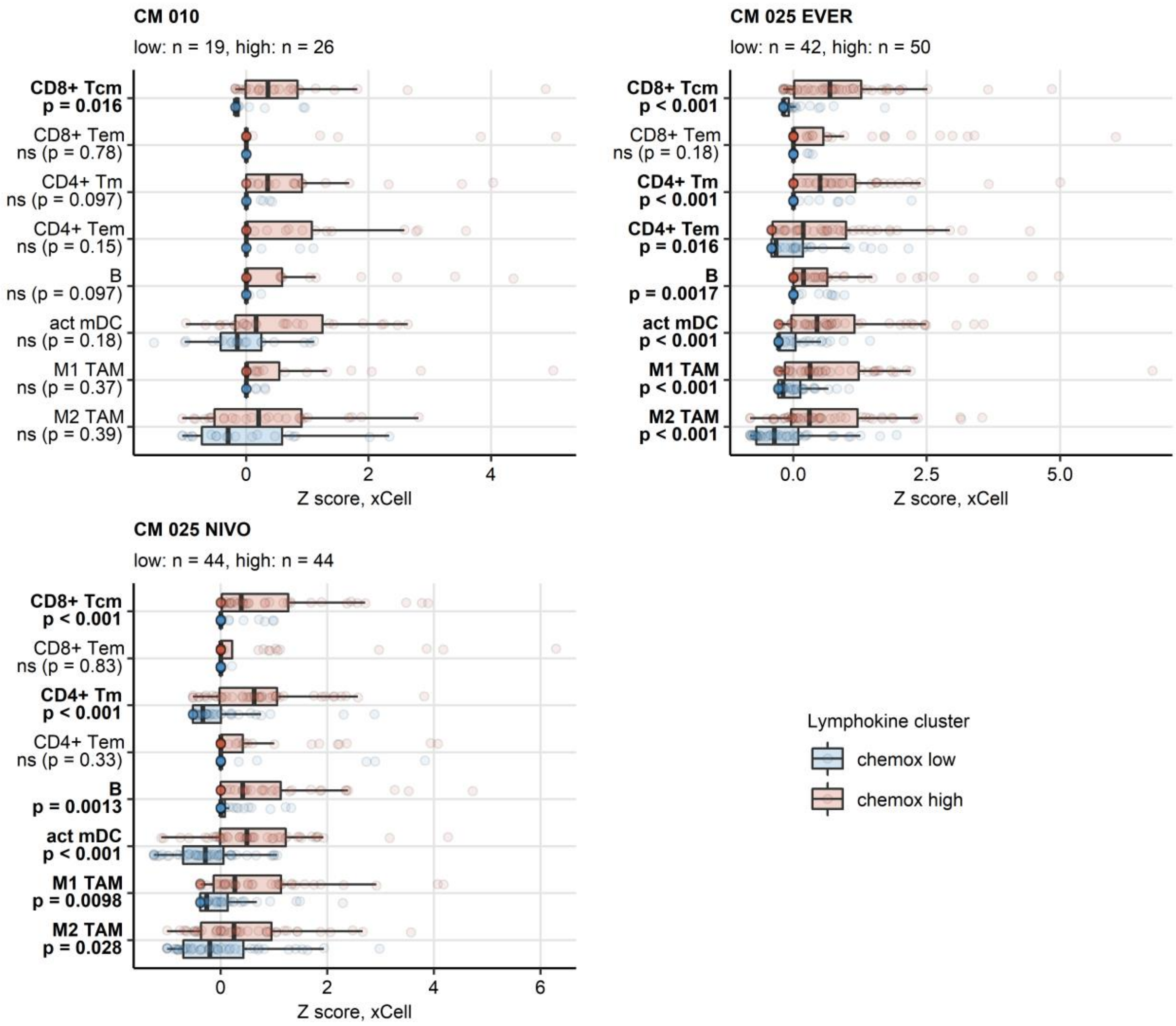

Supplementary Figure S11: Distribution of tumor stages and MSKCC risk strata in the chemokine clusters. Differences in distribution of tumor stages **(A)** and MSKCC risk strata **(B)** between the chemokine (chemox) high and low clusters cancer samples were assessed by false discovery rate (FDR) corrected χ2 test and Cramer V effect size statistic. Effect sizes and p values are displayed in the plot captions, numbers of cancer samples assigned to the chemokine clusters are indicated in the plot x axes. CM 010: CheckMate 010; CM 025 EVER: CheckMate 025 everolimus; CM 025 NIVO: CheckMate 025 nivolumab.

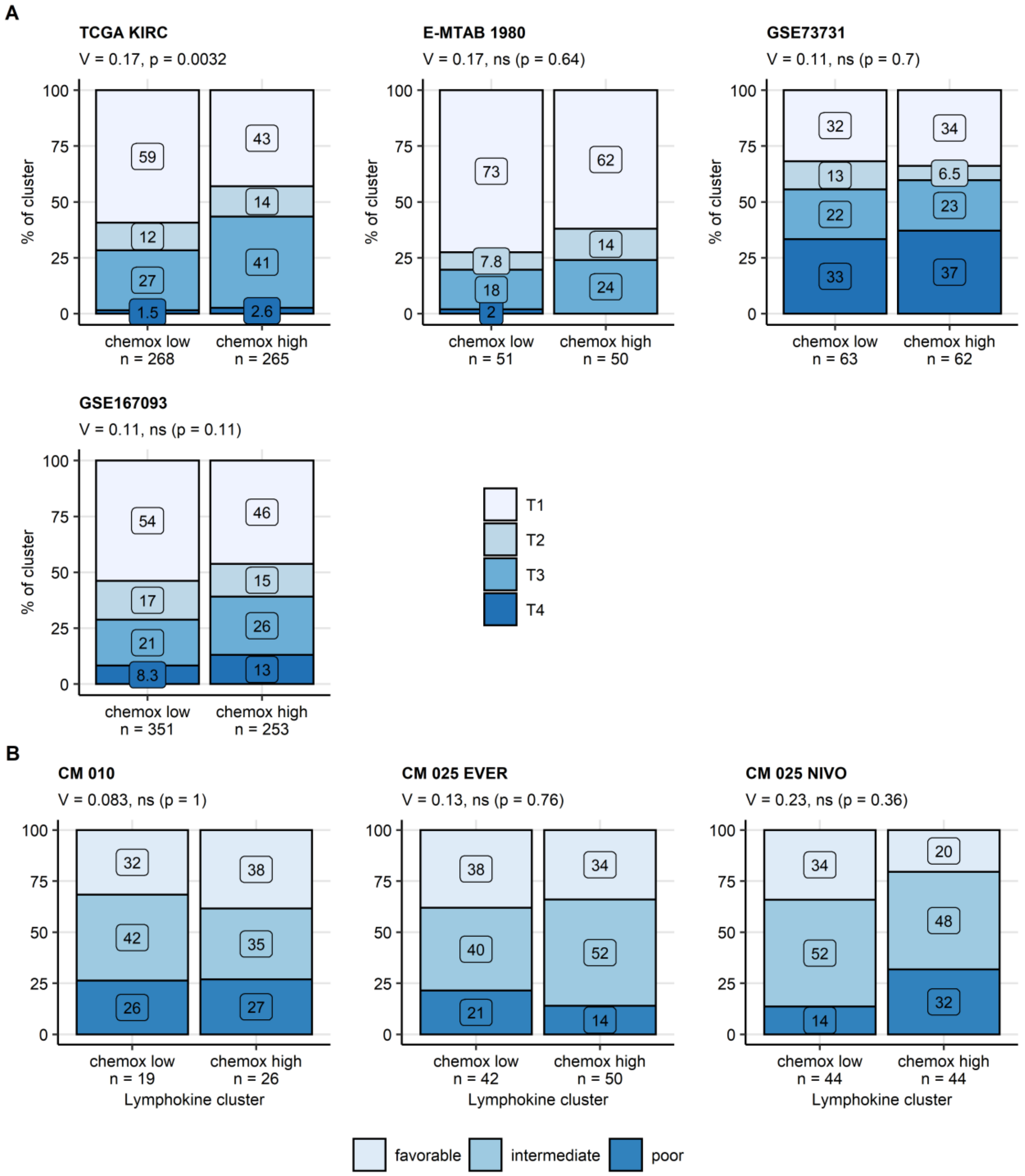

Supplementary Figure S12: Therapy response rates in the chemokine clusters in the CheckMate cohorts. Differences in therapy response rates (complete/partial response [CR/PR] versus stable/progressive disease [SD/PD]) between the chemokine (chemox) high and low clusters cancer samples were assessed by false discovery rate (FDR) corrected χ2 test and Cramer V effect size statistic. Effect sizes and p values are displayed in the plot captions, numbers of cancer samples assigned to the chemokine clusters are indicated in the plot x axes. CM 010: CheckMate 010; CM 025 EVER: CheckMate 025 everolimus; CM 025 NIVO: CheckMate 025 nivolumab.

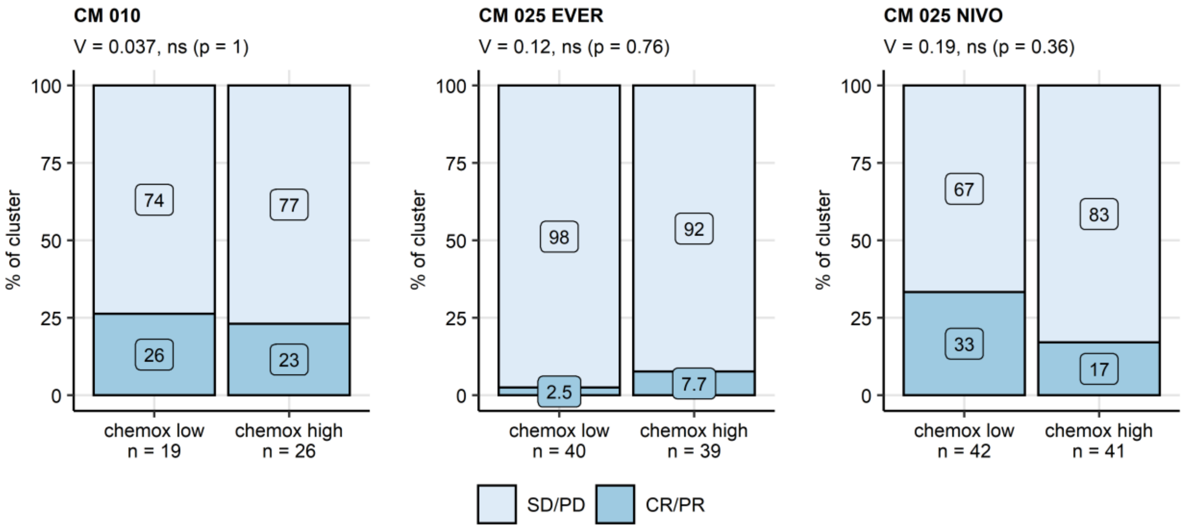

Supplementary Figure S13: Relapse-free survival in the chemokine clusters. Differences in relapse-free survival between the chemokine (chemox) high and low cluster RCC patients were investigated by false discovery rate (FDR) corrected Peto-Peto test. Fractions of relapse-free patients are visualized in Kaplan-Meier plots. Uncorrected and FDRcorrected p values are displayed in the plots. Number of complete observations and relapses (events) are displayed in the plot captions, numbers of patients in the clusters are indicated under the plots. CM 010: CheckMate 010; CM 025 EVER: CheckMate 025 everolimus; CM 025 NIVO: CheckMate 025 nivolumab.

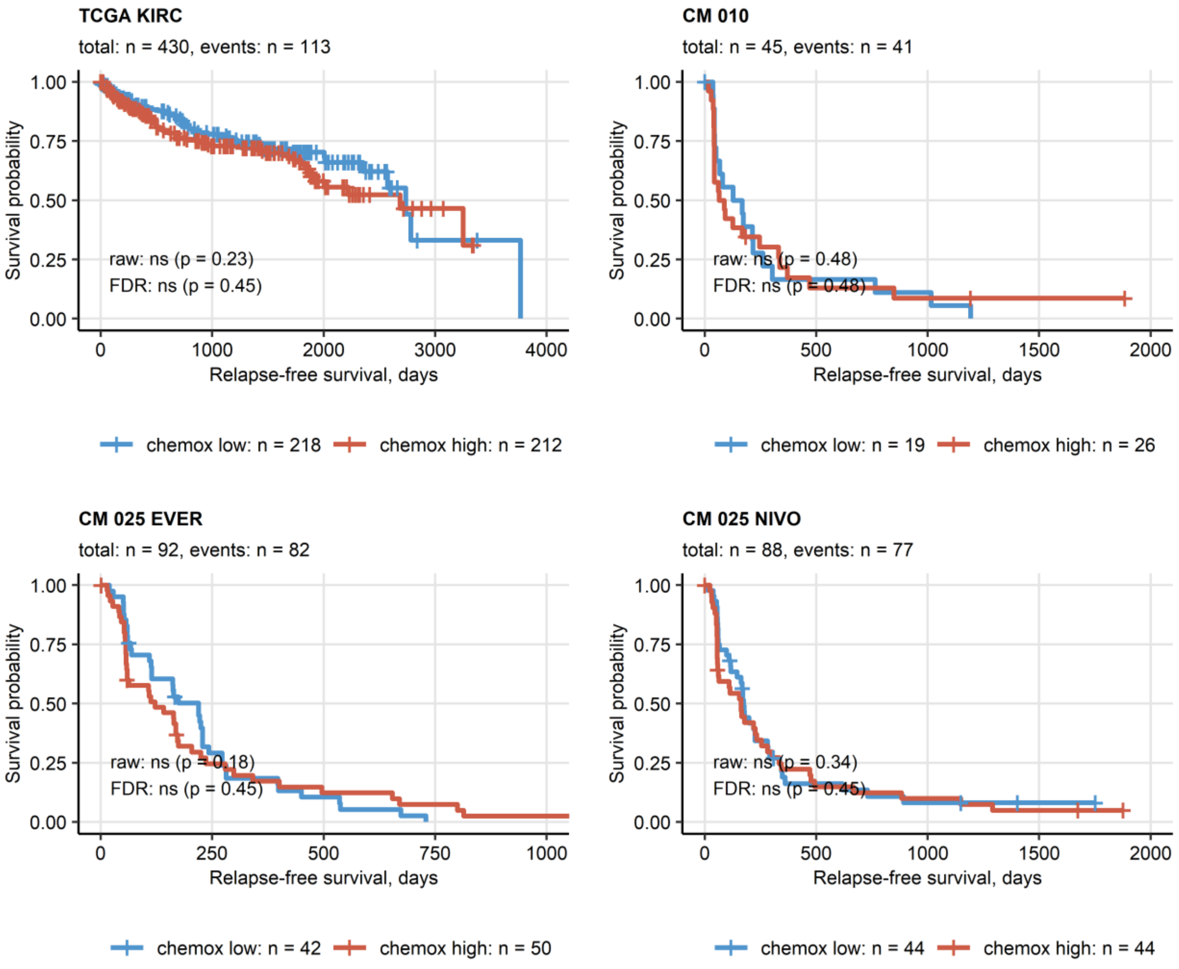

Supplementary Figure S14. Multi-parameter modeling of overall survival as a function of gene expression, demographic and staging data. Association of age, sex, tumor grade, tumor and metastasis stage, and expression of CXCL9/10/11, CXCR3, CXCL13, CXCR5, XCL1, XCR1 with overall survival was assessed by multi-parameter Ridge Cox regression. Two Ridge models were trained in the TCGA KIRC cohort: a model with the demographic, clinical and gene of interest expression data and a model with the demographic and clinical data only. Performance of the models wassubsequently validated in the E-MTAB 1980 and RECA-EU cohort. **(A)** Hazard ratio (HR) estimates of the full demographic/clinical/gene expression data Ridge model in the TCGA KIRC cohort presented in a bubble plot. Point size corresponds to the absolute value of log HR, point color codes for the correlation sign (red: unfavorable, blue: favorable association with survival).

(B) Concordance **(C)** indexes with 95% confidence intervals (CI) for the demographic/clinical and the demographic/clinical/gene expression survival models in the training TCGA KIRC cohort, and the validation RECA-EU and E-MTAB 1980 collectives. The results are presented in a Forest plot. Numbers of patients and deaths are indicated in the Y axis.

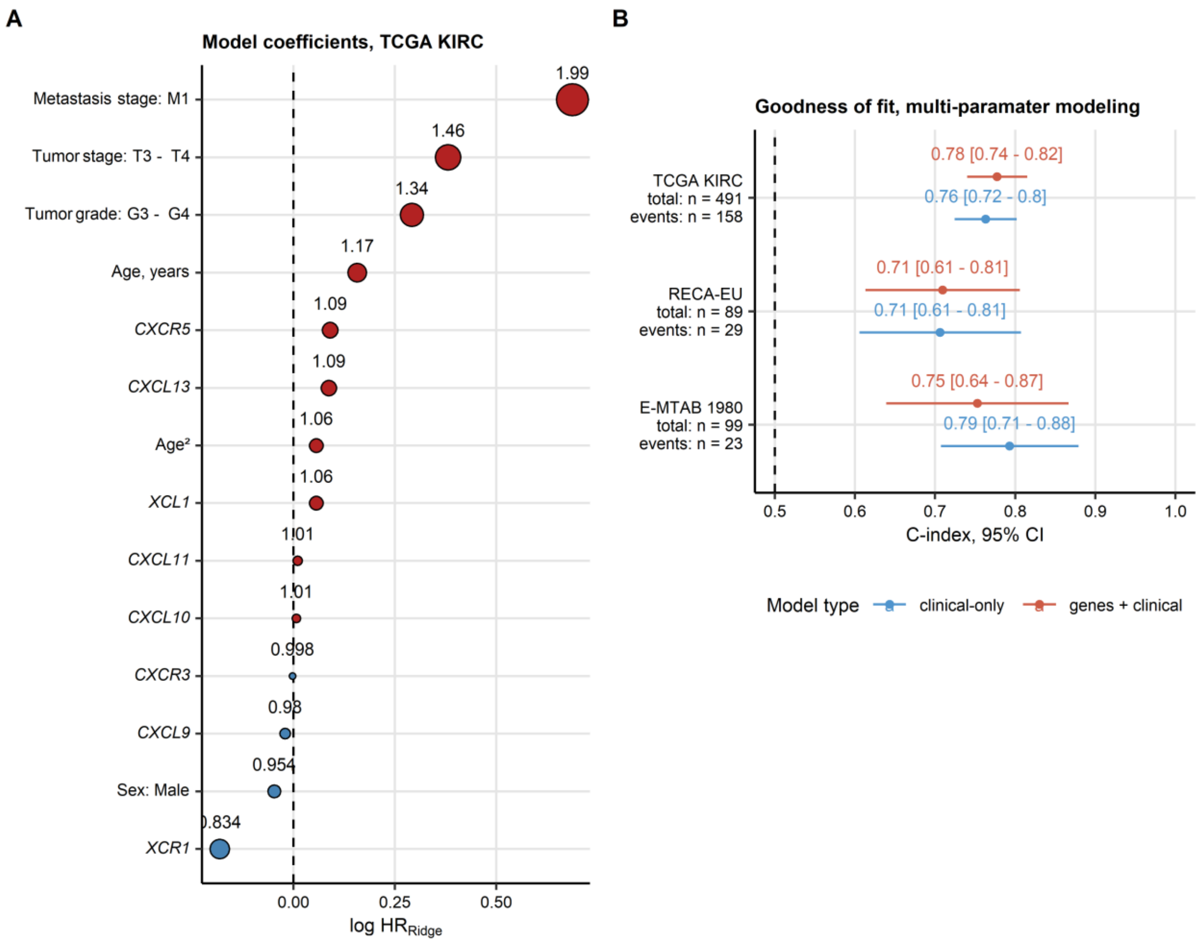

Supplementary Figure S15: Predicted activation of signaling pathways in the chemokine high versus chemokine low cluster. Modulation of KEGG-listed signaling pathways based on differential gene expression in the chemokine (chemox) high and chemokine low cluster cancers was investigated with the SPIA algorithm. Pathways found to be significantly modulated in all study collectives except for the smallest CheckMate 010 cohort (n = 10) were identified. The tA metric activity regulation estimates (chemokine high versus chemokine low cluster) for the significantly regulated pathways are presented in a bubble plot. Point size corresponds to the absolute value of tA, point color codes for the tA value. Points are labeled with their tA values. CM 010: CheckMate 010; CM 025 EVER: CheckMate 025 everolimus; CM 025 NIVO: CheckMate 025 nivolumab.

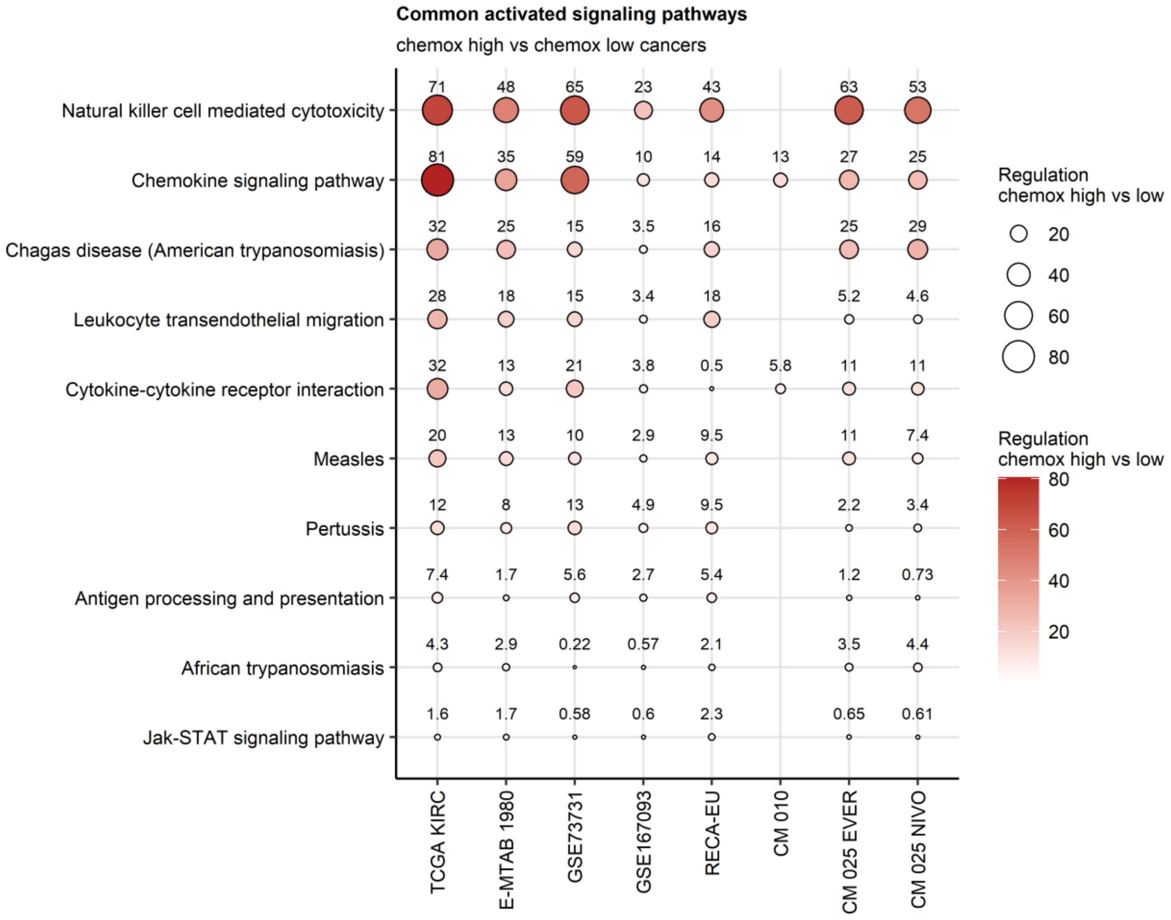

Supplementary Figure S16: Differential expression of interferon gamma signaling associated genes between the chemokine clusters in the TCGA KIRC, E-MTAB 1980, GSE73731 and GSE167093 cohorts. Genes differentially expressed in the chemokine (chemox) high versus chemokine low cancers were identified by false discovery rate (FDR) corrected two-tailed T test and the > 1.25 fold regulation cutoff. Significance and fold-regulation estimates for genes associated with interferon alpha/beta and interferon gamma Reactome pathways are presented in volcano plots. Points represent single genes, point color codes for the regulation sign. The significance and fold regulation cutoffs are visualized as dashed lines. Top significantly regulated genes are labeled with their symbols. Numbers of significantly up- and downregulated genes are displayed in the plot captions.

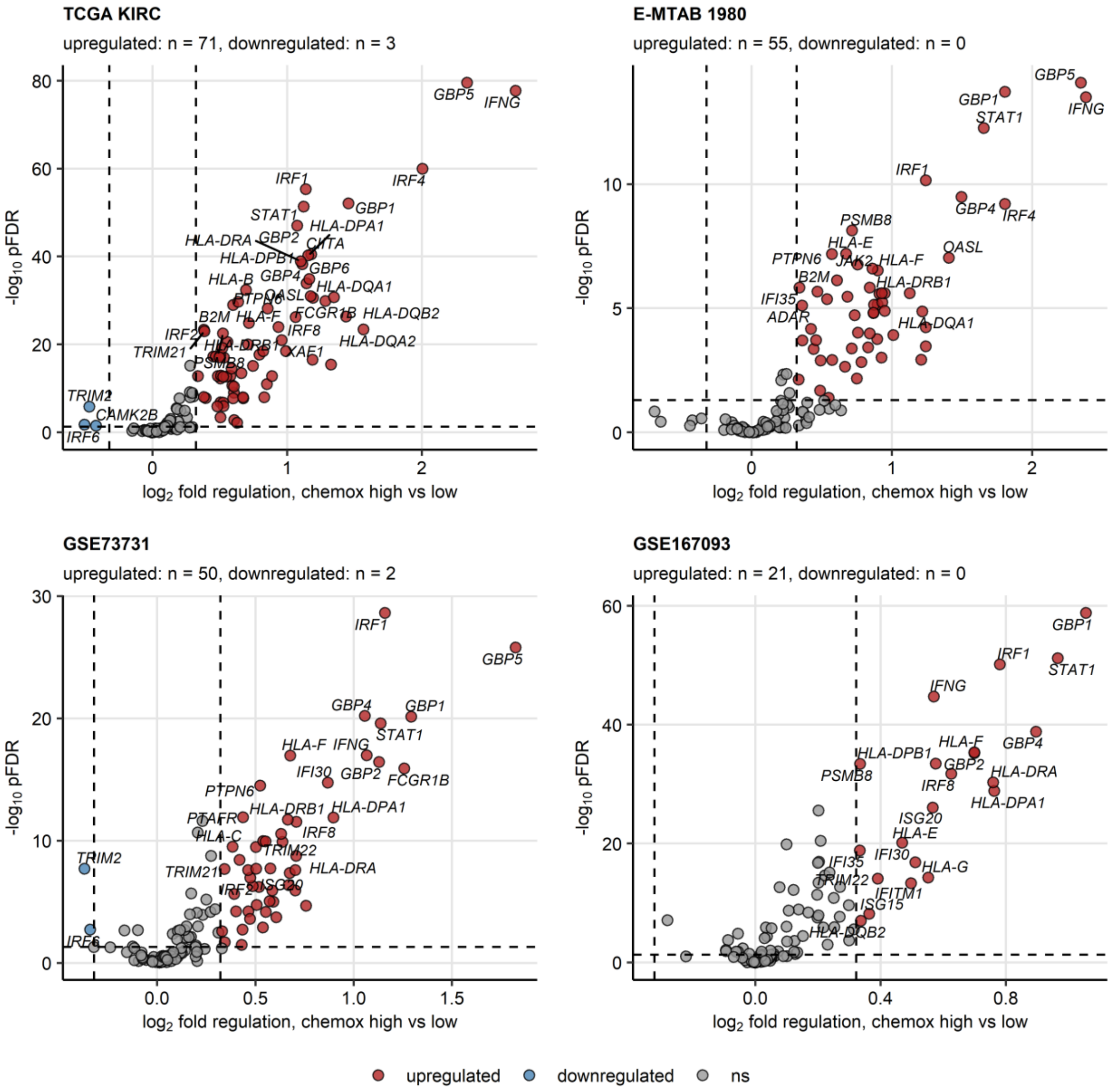

Supplementary Figure S17: Differential expression of interferon gamma signaling associated genes between the chemokine clusters in the CheckMate cohorts. Genes differentially expressed in the chemokine (chemox) high versus chemokine low cancers were identified by false discovery rate (FDR) corrected two-tailed T test and the > 1.25 fold regulation cutoff. Significance and fold-regulation estimates for genes associated with interferon alpha/beta and interferon gamma Reactome pathways are presented in volcano plots. Points represent single genes, point color codes for the regulation sign. The significance and fold regulation cutoffs are visualized as dashed lines. Top significantly regulated genes are labeled with their symbols. Numbers of significantly up- and downregulated genes are displayed in the plot captions. CM 010: CheckMate 010; CM 025 EVER: CheckMate 025 everolimus; CM 025 NIVO: CheckMate 025 nivolumab.

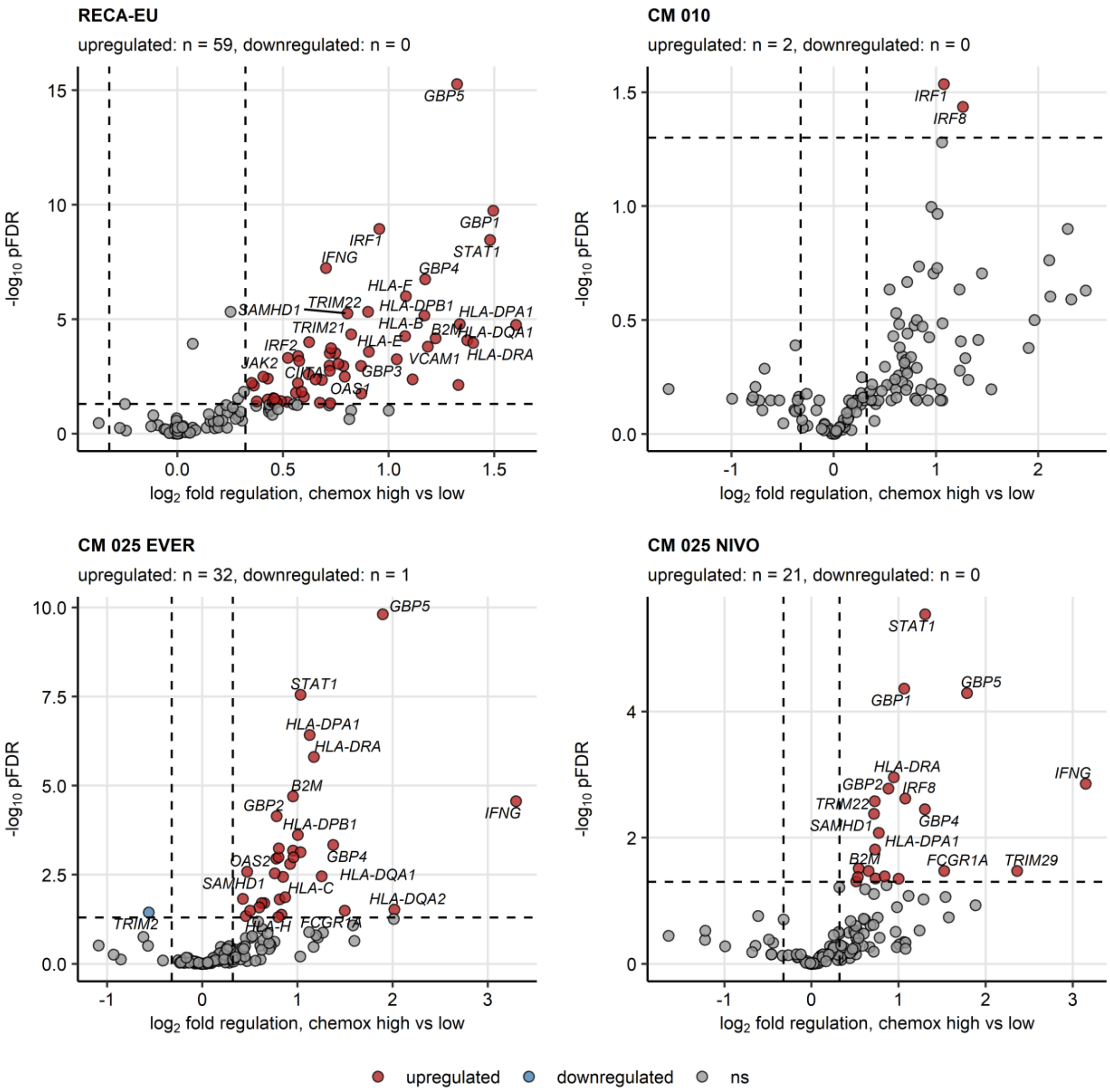

Supplementary Figure S18: Differential expression of T cell exhaustion-associated genes between the chemokine clusters in the TCGA KIRC, E-MTAB 1980, GSE73731 and GSE167093 cohorts. Genes differentially expressed in the chemokine (chemox) high versus chemokine low cancers were identified by false discovery rate (FDR) corrected two-tailed T test and the > 1.25 fold regulation cutoff. Significance and fold-regulation estimates for genes associated with T cell exhaustion are presented in volcano plots. Points represent single genes, point color codes for the regulation sign. The significance and fold regulation cutoffs are visualized as dashed lines. Top significantly regulated genes are labeled with their symbols. Numbers of significantly up- and downregulated genes are displayed in the plot captions.

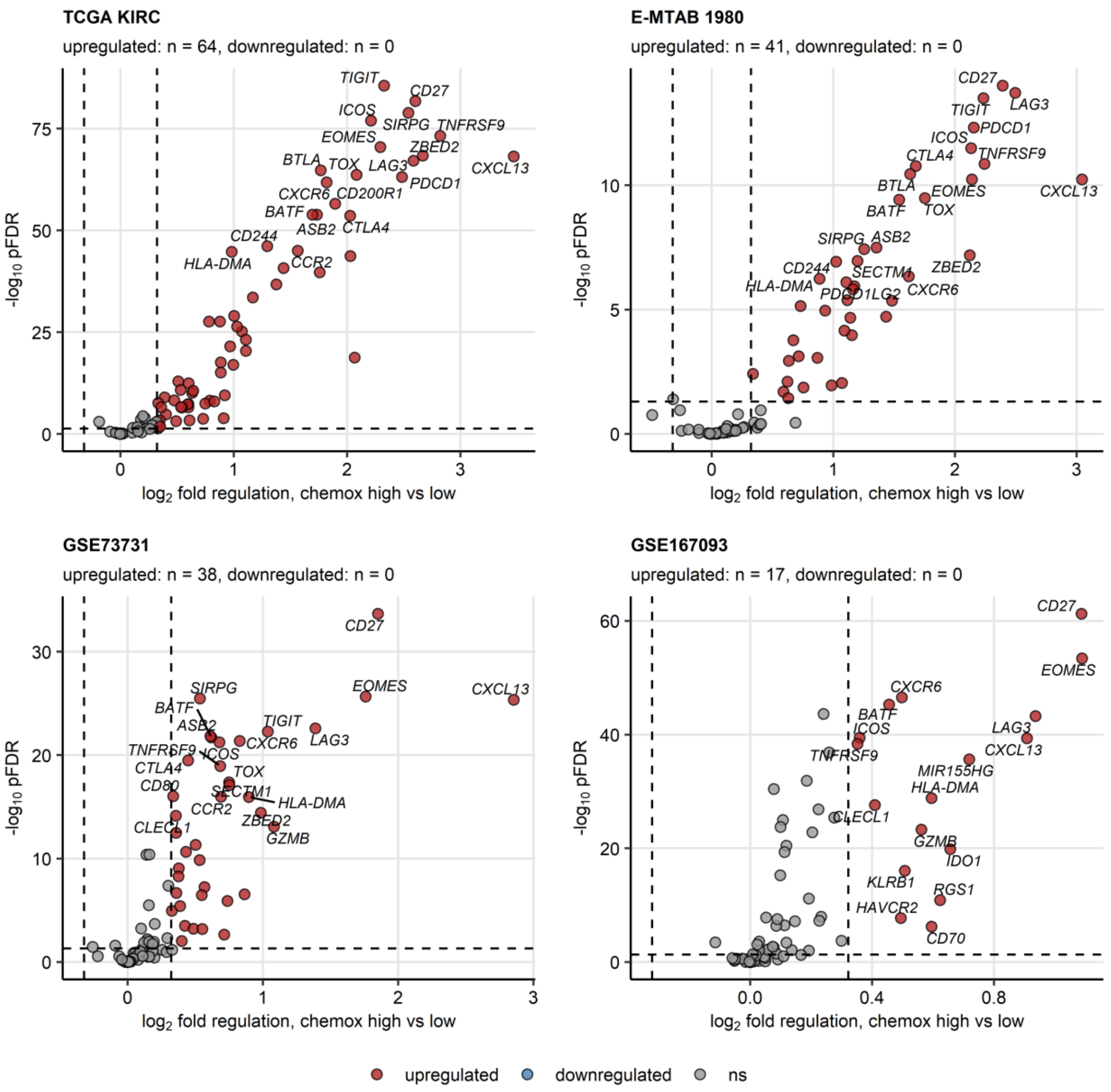

Supplementary Figure S19: Differential expression of T cell exhaustion-associated genes between the chemokine clusters in the CheckMate cohorts. Genes differentially expressed in the chemokine (chemox) high versus chemokine low cancers were identified by false discovery rate (FDR) corrected two-tailed T test and the > 1.25 fold regulation cutoff. Significance and fold-regulation estimates for genes associated with T cell exhaustion are presented in volcano plots. Points represent single genes, point color codes for the regulation sign. The significance and fold regulation cutoffs are visualized as dashed lines. Top significantly regulated genes are labeled with their symbols. Numbers of significantly up- and downregulated genes are displayed in the plot captions. CM 010: CheckMate 010; CM 025 EVER: CheckMate 025 everolimus; CM 025 NIVO: CheckMate 025 nivolumab.

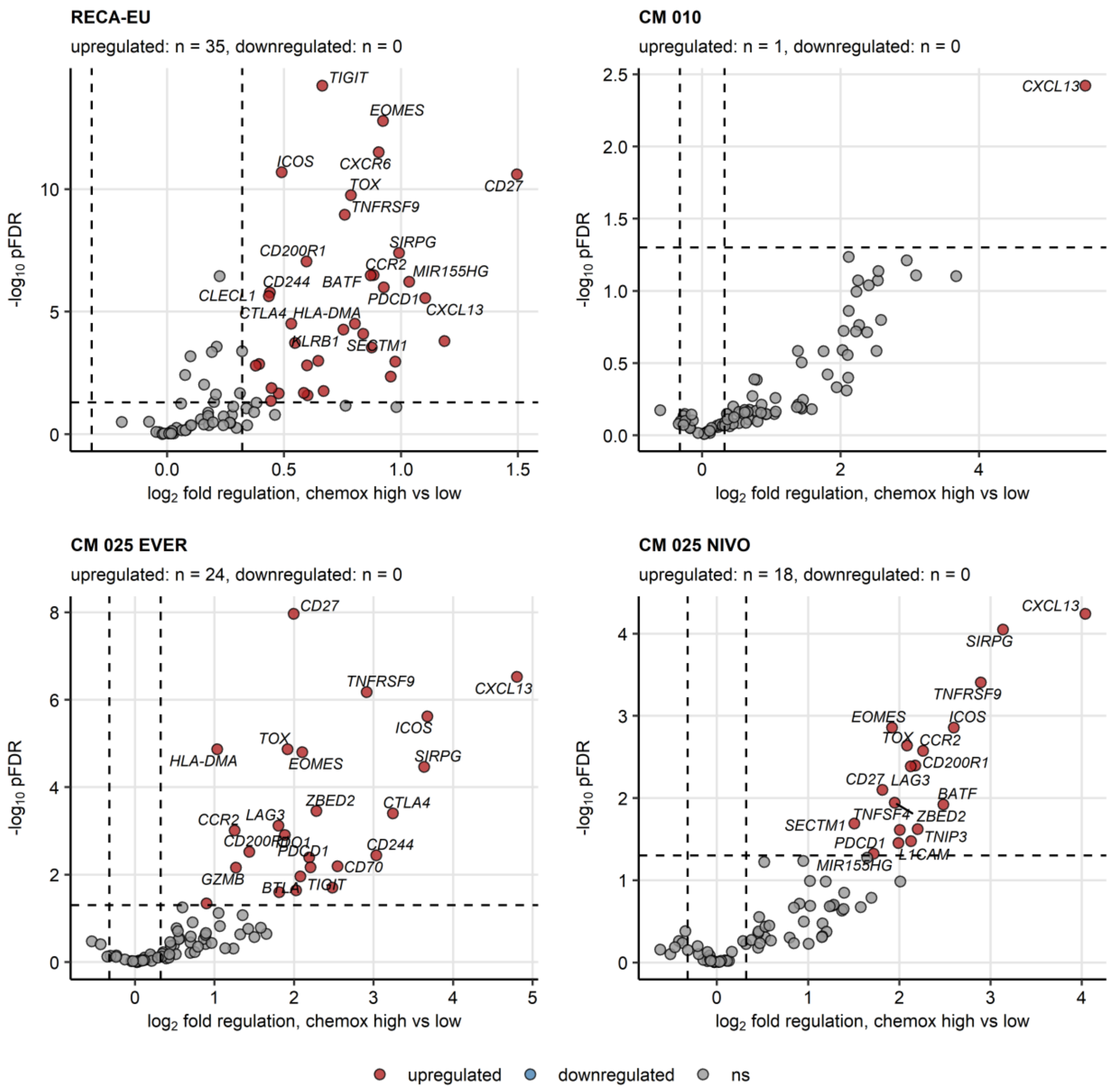

Supplementary Figure S20: Differential expression of genes coding for clinically relevant immune checkpoint proteins in the chemokine clusters in the CheckMate cohorts. Genes differentially expressed in chemokine (chemox) high versus chemokine low cancers were identified by false discovery rate (FDR) corrected two-tailed T test. Results for clinically relevant immune checkpoint genes are presented. Median normalized log_2 expression levels with interquartile ranges (IQR) are presented as boxes, whiskers span over the 150% IQR. Points represent single cancer samples. P values are indicated in the Y axes, significant effects are highlighted with bold. Numbers of cancer samples assigned to the chemokine high and low clusters are displayed in the plot captions. CM 010: CheckMate 010; CM 025 EVER: CheckMate 025 everolimus; CM 025 NIVO: CheckMate 025 nivolumab.

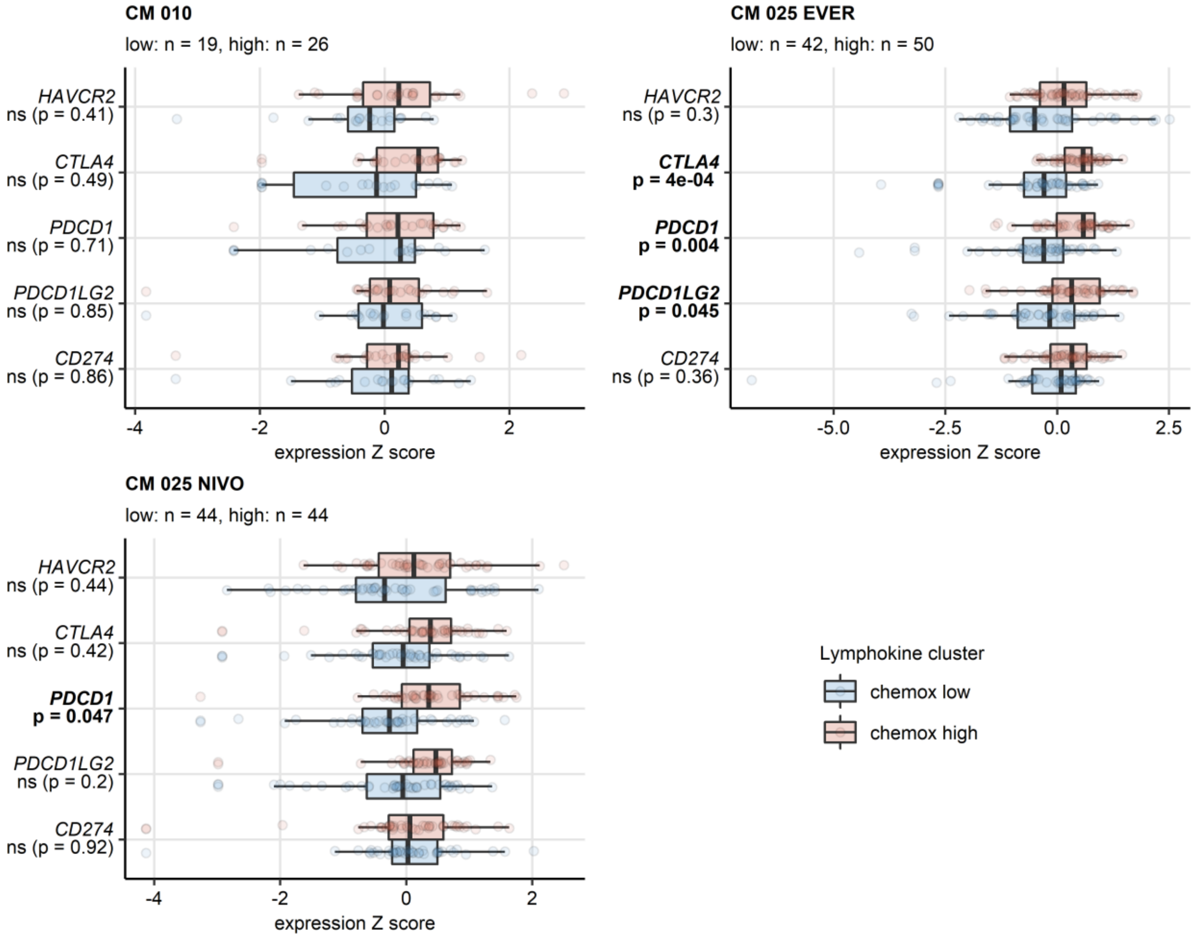

Supplementary Figure S21: Predicted differences in activity of fatty acid oxidation reactions between the chemokine clusters. Regulation of the Recon human metabolism model reactions in chemokine (chemox) high versus chemokine low cluster cancers was predicted based on the differential gene expression with the BiGGR and biggrExtra package tools. Significance of the metabolic reaction regulation was assessed by Monte Carlo simulation and corrected for multiple testing with the false discovery rate (FDR) method. Predicted fold-regulation estimates for fatty acid oxidation reactions (FAO, total reactions: n = 678) in the chemokine high versus chemokine low cluster are presented in bar plots. Bar color codes for significance and the regulation sign. Numbers of significantly activated and inhibited FAO reactions are displayed in the plot captions. CM 010: CheckMate 010; CM 025 EVER: CheckMate 025 everolimus; CM 025 NIVO: CheckMate 025 nivolumab.

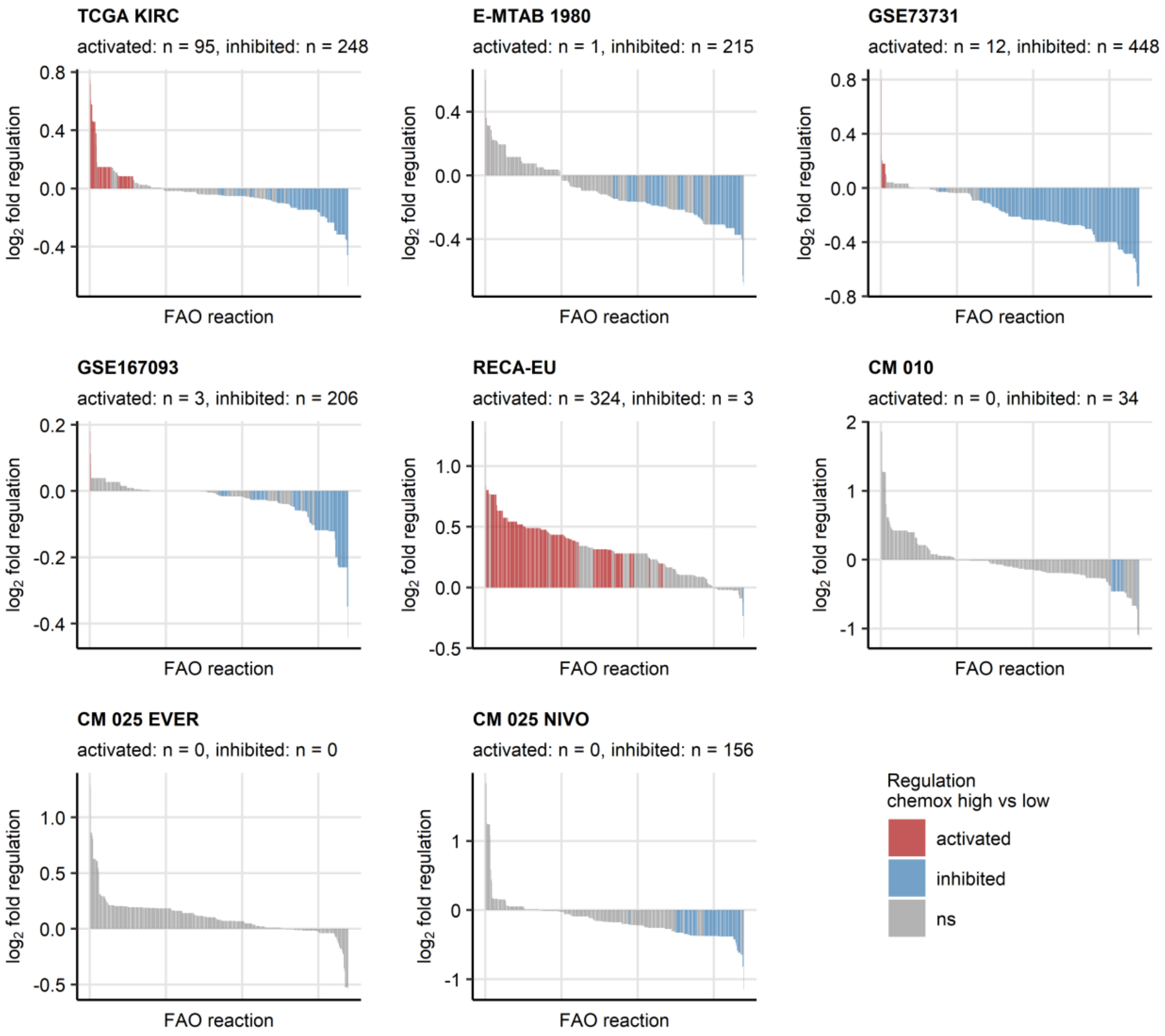

Supplementary Figure S22: Predicted differences in activity of citric acid cycle reactions between the chemokine clusters. Regulation of the Recon human metabolism model reactions in chemokine high versus chemokine low cluster cancers was predicted based on the differential gene expression with the BiGGR and biggrExtra package tools. Significance of the metabolic reaction regulation was assessed by Monte Carlo simulation and corrected for multiple testing with the false discovery rate (FDR) method. Predicted fold-regulation estimates for subsequent citric acid cycle reactions **(A)** in the chemokine high versus chemokine low cluster are presented in bar plots **(B)**. Bar color codes for significance and the regulation sign. ACON: aconitase; ACON mito: mitochondrial aconitase; IDH/NAD: isocitrate dehydrogenase - NAD; IDH/NADP: isocitrate dehydrogenase - NADP; IDH/NADP mito: mitochondrial IDH/NADP; IDH/NADP+: oxalosuccinate:NADP+ oxidoreductase; OGDH: 2-oxoglutarate dehydrogenase; SCS/GTP: succinate -CoA ligase GTP-forming; SCS/ATP: succinate-CoA ligase ATP-forming; SDH:succinate dehydrogenase; FH: fumarase; FH mito: mitochondrial fumarase; MDH: malate dehydrogenase; MDH mito: mitochondrial malate dehydrogenase; CS: citrate synthase, CM 010: CheckMate 010; CM 025 EVER: CheckMate 025 everolimus; CM 025 NIVO: CheckMate 025 nivolumab.

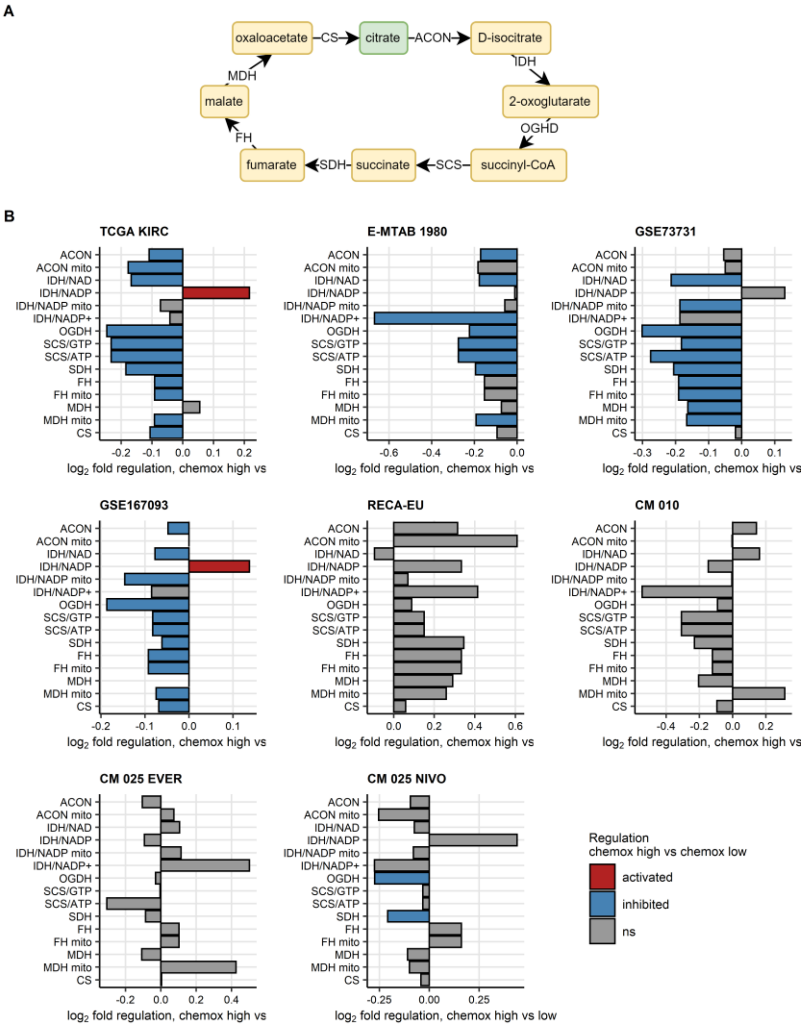

Supplementary Figure S23: Predicted differences in activity of oxidative phosphorylation complexes between the chemokine clusters. Regulation of the Recon human metabolism model reactions in chemokine high versus chemokine low cluster cancers was predicted based on the differential gene expression with the BiGGR and biggrExtra package tools. Significance of the metabolic reaction regulation was assessed by Monte Carlo simulation and corrected for multiple testing with the false discovery rate (FDR) method. Predicted fold-regulation estimates for subsequent oxidative phosphorylation (OxPhos) complexes in the chemokine high versus chemokine low cluster are presented in bar plots. Bar color codes for significance and the regulation sign. CM 010: CheckMate 010; CM 025 EVER: CheckMate 025 everolimus; CM 025 NIVO: CheckMate 025 nivolumab.

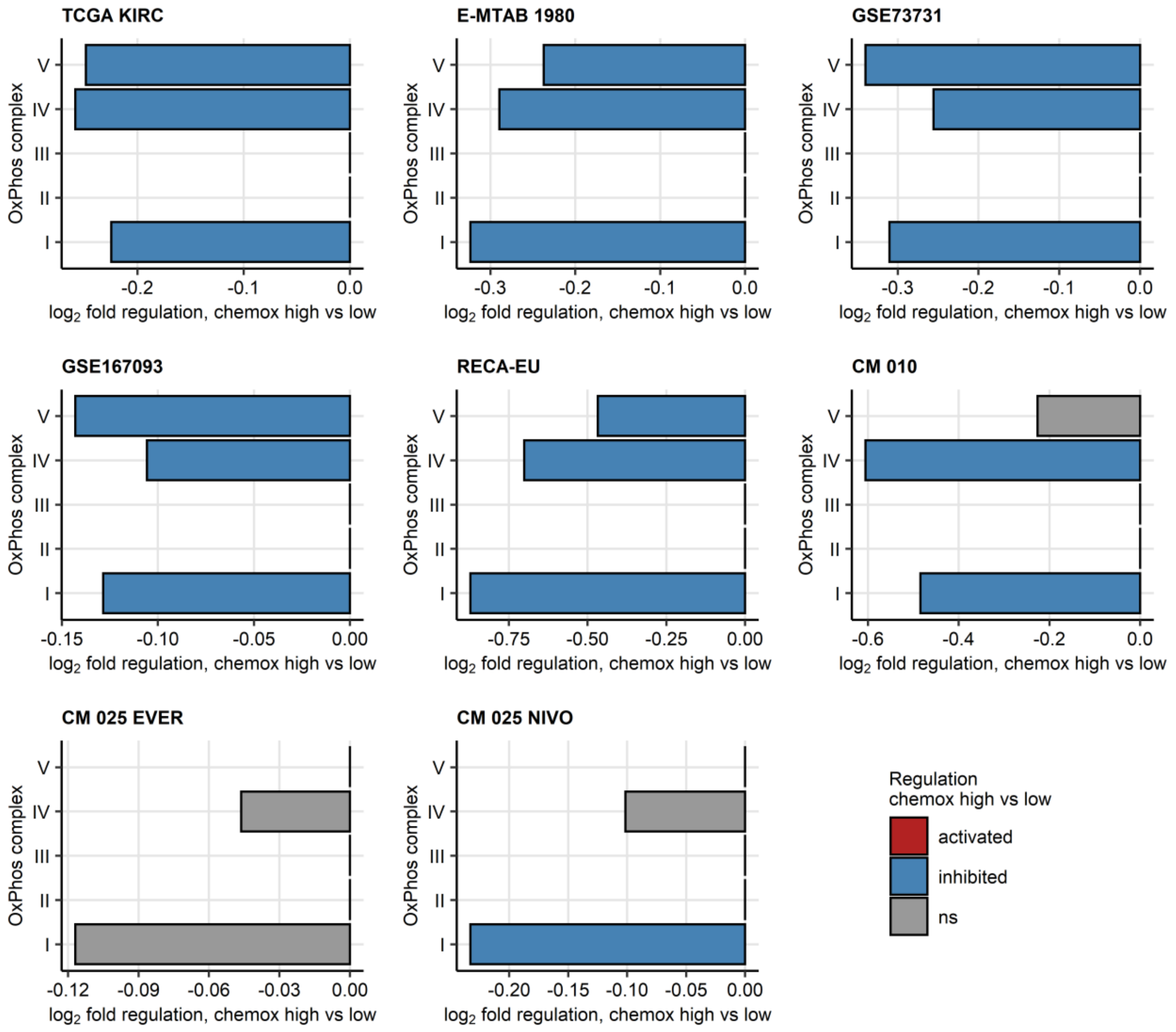

Supplementary Figure S24: Predicted differences in activity of the tryptophan/kynurenine/quinolinate pathway between the chemokine clusters. Regulation of the Recon human metabolism model reactions in chemokine high versus chemokine low cluster cancers was predicted based on the differential gene expression with the BiGGR and biggrExtra package tools. Significance of the metabolic reaction regulation was assessed by Monte Carlo simulation and corrected for multiple testing with the false discovery rate (FDR) method.

(A) Pathway scheme. TRP: tryptophan; TDO: tryptophan 2,3-dioxygenase; KMO: kynurenine 3-monooxygenase; KYNU: kynureninase; KAT: kynurenin aminotransferase; HAAO: 3-hydroxyanthranilate 3,4-dioxygenase.

(B) Predicted fold-regulation estimates for the tryptophan - kynurenine - quinolinate pathway in the chemokine high versus chemokine low cluster presented in bar plots. Bar color codes for significance and the regulation sign. CM 010: CheckMate 010; CM 025 EVER: CheckMate 025everolimus; CM 025 NIVO: CheckMate 025 nivolumab.

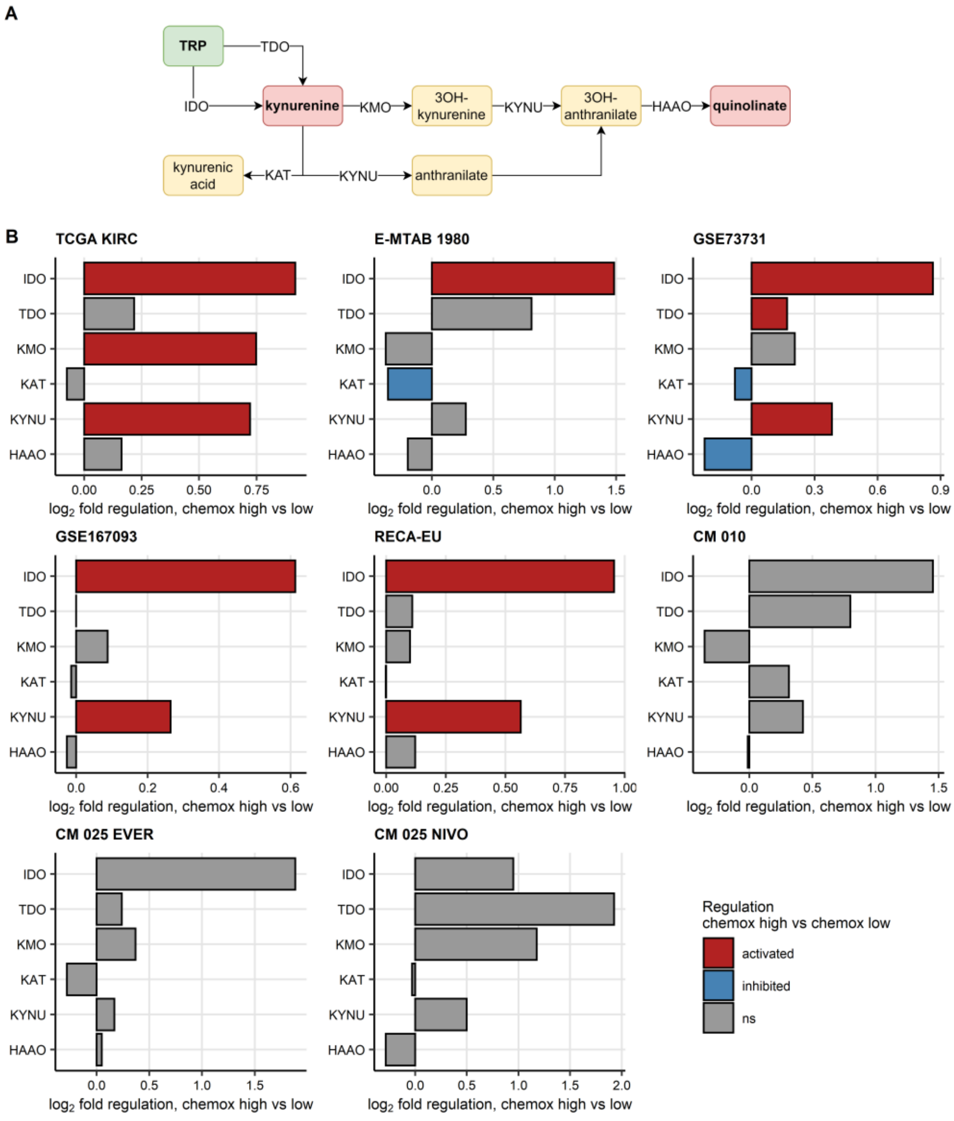

Supplementary Figure S25: Differential expression of genes involved in the tryptophan/kynurenine/quinolinate metabolism in the chemokine clusters in the CheckMate cohorts. Genes differentially expressed in chemokine (chemox) high versus chemokine low cancers were identified by false discovery rate (FDR) corrected two-tailed T test. Results for genes involved in the tryptophan - kynurenin - quinolinate metabolic pathway are presented. Median normalized log2 expression levels with interquartile ranges (IQR) are presented as boxes, whiskers span over the 150% IQR. Points represent single cancer samples. P values are indicated in the Y axes, significant effects are highlighted on bold. Numbers of cancer samples assigned to the chemokine high and low clusters are displayed in the plot captions. IDO: indoleamine 2,3-dioxygenase; TDO: tryptophan 2,3-dioxygenase; KYAT1: kynurenine—oxoglutarate transaminase 1; KYNU: kynureninase; KMO: kynurenine 3-monooxygenase; KAT: kynurenin aminotransferase; HAAO: 3-hydroxyanthranilate 3,4-dioxygenase; CM 010: CheckMate 010; CM 025 EVER: CheckMate 025 everolimus; CM 025 NIVO: CheckMate 025 nivolumab.

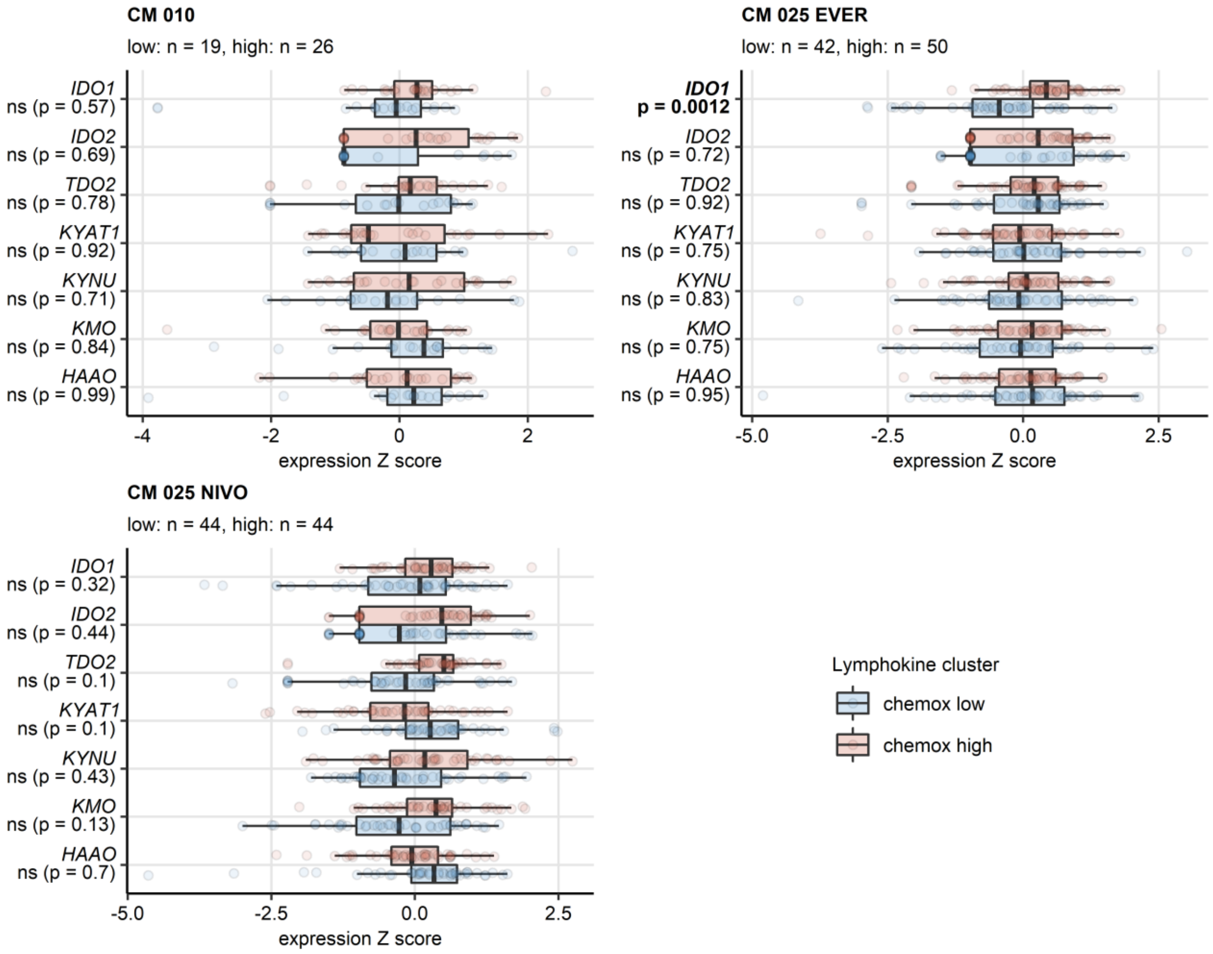

Supplementary Methods

Data import and transformation

Clinical information and normalized RNA-seq expression estimates for the Kidney Clear Cell Carcinoma (KIRC) TCGA project (1) were downloaded from the GDC Data Portal with the help of the TCGA-Assembler script (https://github.com/compgenome365/TCGAAssembler-2/blob/master/TCGA-Assembler/). Clinical and normalized RNAseq expression data for the RECA-EU cohort were fetched from https://dcc.icgc.org/projects/RECA-EU. Clinical information and normalized RNA-seq expression data for the CheckMate 010 (CM 010) and CheckMate 025 (CM 025) cohorts were extracted from the report of Braun et al. (2) (Tables S1 and S4 accompanying the paper). To get more insight at possible effects of therapy with immune checkpoint inhibitors, the CheckMate 025 everolimus (CM 025 EVER) and nivolumab (CM 025 NIVO) treatment arms were analyzed separately in the current report. Clinical information and normalized RNA-seq data for the RECA-EU study were extracted from the ICGC Data Portal (3).
Normalized microarray expression data and clinical information for the GSE73731 (4) and GSE167093 (5) cohorts (platforms: Affymetrix Human Genome U133 Plus 2.0 and Illumina HumanHT-12 V4.0 expression beadchip, respectively) were extracted from the GEO API with the *GEOquery* package (6).
Normalized microarray expression data (Agilent Human Gene Expression 4x44K v2 Microarray) and clinical information for the E-MTAB 1980 (7) cohort were downloaded from the ArrayExpress portal. For the microarray expression studies, integration of multiple probes targeting the same gene was accomplished by geometric mean. The clinical characteristic of the analyzed studies is provided in **Table 1**.
Expression values were treated with the $log_2(transcript count+1)$ (RNA sequencing) of log_2 transformation (microarray) to improve normality. The chemokine genes of interest (*CXCL9/10/11*, *CXCL13* and *XCR1*) were identified to be preferentially expressed in the RCC tissue as compared with the normal kidney and correlate with predicted tumor-infiltrating $CD8^+$ T cell levels as described in more details below. Estimates of non-malignant and immune infiltration in RCC were calculated using the QuanTIseq (8) and xCell (9) algorithms implemented in R by the *immunedeconv* package (10). Gene signatures corresponding to Reactome pathways were extracted from a local copy of the MSig database (version 7.5.1, http://www.gsea-msigdb.org/gsea/index.jsp) and the signatures' single sample gene set enrichment analysis scores (ssGSEA) were calculated with the *GSVA* (11) and the development *gseaTools* package (https://github.com/PiotrTymoszuk/gseaTools). Genes associated with T cell exhaustion were retrieved from four recent papers (12–15).
Genes associated with interferon alpha/beta signaling and interferon gamma signaling were obtained from the Reactome database (pathways: R-HSA-909733 and R-HSA-877300).

Software

The statistical and bioinformatic analysis was done with R 4.2.0. Tabular data were handled with the *tidyverse* package bundle (16) and the development package *trafo* (https://github.com/PiotrTymoszuk/trafo). Text data were transformed with the *stringi* package (17). Prediction of non-malignant cell content was done with the *immunedeconv* package (10) employing the xCell and QuanTIseq (8) algorithms.

Gene signatures were retrieved, stored and their scores calculated with the *gseaTools* development package (https://github.com/PiotrTymoszuk/gseaTools). Parallelization was accomplished with *furrr* (18).
Network analysis of the xCell infiltration data was accomplished with the *igraph* (19) and *network* (20) packages. Semi-supervised clustering was done with the development package *clustTools* (https://github.com/PiotrTymoszuk/clustTools) providing a common interface to multiple dimensionality reduction and clustering algorithms and employing among others algorithms from the packages *cluster* (21), *philentropy* (22), *factoextra* (23), *dbscan* (24) and *umap* (25). Exploratory analysis and statistical hypothesis testing (comparison and correlation) was done with the *rstatix* (26) package and the development package *ExDA*. For survival analysis, the packages *survival* (27), *survminer* (28), *glmnet* (29) and the development package *coxExtensions* (https://github.com/PiotrTymoszuk/coxExtensions) were employed. Differential gene expression analysis and comparison of Reactome ssGSEA score between the clusters was done with the development package *microViz* (https://github.com/PiotrTymoszuk/microViz). Modulation of signaling pathways was investigated with the *SPIA* algorithm (30). Analysis of differences in metabolic reaction activity was done with the *Biggr* (31) package and the development package *biggrExtra* (https://github.com/PiotrTymoszuk/biggrExtra).
Visualization of the analysis results was accomplished with *ggplot2* (32), *cowplot* (33), *ExDA* (violin, box and stack plots), *microViz* (Forest plots, bar plots of regulation estimates and p values, volcano plots). Network plots were created with *ggnetwork* (34). Kaplan-Meier plots were generated with *survminer* (28).
Parts of the manuscript and supplementary material were written in the *rmarkdown* environment (35) and rendered as Word and pdf files with *bookdown* (36) and *knitr* (37). Tables were generated with the *flextable* package (38). Management of figures and tables in the Rmarkdown documents (insertion, referencing) was done with the development package *figur* (https://github.com/PiotrTymoszuk/figur).

Characteristic of the study cohorts

Numeric variables are presented as medians with interquartile ranges. Categorical variables are shown as percentages and counts within the complete observation set. Descriptive statistic for demographic and clinical features of the investigated cohorts was done with *ExDA* (function explore()).

Genes of interest

The chemokine genes of interest were identified within the set of 28 chemokine genes available for all investigated datasets (*CCL2, CCL7, CCL8, CCL11, CCL13, CCL17, CCL20, CCL21, CCL22, CCL24, CCL25, CCL26, CCL28, CXCL1, CXCL2, CXCL3, CXCL5, CXCL6, CXCL9, CXCL10, CXCL11, CXCL12, CXCL13, CXCL14, CXCL16, CXCL17, CX3CL1, XCL1*). The chemokine genes were screened in the TCGA KIRC cohort for differences in expression levels between the RCC and normal kidney tissue (paired normal/tumor samples, two-tailed paired T test, function test_two_groups(), *microViz* package), and for correlation with $CD8^{+}$ T cell content in RCC predicted with the QuanTIseq algorithm (8) (Spearman's correlation, correlate_variables(), package *ExDA*). In these analyses expression of *CXCL9* (Entrez ID: 4283), *CXCL10* (3627), *CXCL11* (6373), *CXCL13* (10563) and *XCL1*

(6375) was found to be highly enriched in the malignant tissue and strongly significantly associated with the predicted $CD8^+$ T cell levels (**Supplementary Figure S1**). These chemokine genes along with their cognate receptors *CXCR3* (Entrez ID: 2833, receptor for CXCL9/10/11), *CXCR5* (643, receptor for CXCL13) and *XCR1* (2829, receptor for XCL1) were investigated further in the current report.

Comparison of gene expression between the normal and tumor samples

log_2-transformed expression levels of the genes of interest (*CXCL9, CXCL10, CXCL11, CXCR3, CXCL13, CXCR5, XCL1, XCR1*) were compared between the donor-matched normal kidney and cancer samples in the TCGA and GSE167093 cohorts with two-tailed paired T test corrected for multiple testing with false discovery rate (FDR) method (39) and Cohen's d effect size statistic (function compare_variables(), package *ExDA*, employing tools from *rstatix*) (26).

Expression of the genes of interest and non-malignant cell infiltration

Association of the log_2-transformed expression levels of the genes of interest with the QuanTIseq and xCell estimated content of non-tumor cells in the cancer sample (8–10) was investigated with Spearman's correlation (function correlate_variables(), package *ExDA*) with FDR adjustment for multiple testing (39).
In addition, min/max scaled Spearman's correlations between the genes of interest and xCell-predicted infiltrating cell types were calculated (function cor(method = 'spearman'), package *stats*). The correlation matrices for the genes of interest and cell
types associated with them with moderate-to-large strength (correlation coefficient ρ >0.4) were visualized as force-directed network plots with edge weighting by the scaled Spearman's correlation coefficient (functions graph_from_adjacency_matrix(weigthed =
TRUE), package *igraph*, and functions provided by the *ggnetwork* package) (19,20,34).

Survival modeling

Correlation of log_2-transformed cancer expression levels of the genes of interest with overall (TCGA KIRC, E-MTAB 1980, RECA-EU and CheckMate cohorts) and relapse-free survival (TCGA KIRC, CheckMate) was investigated by uni-variable Cox proportional hazard modeling (function coxph(), package *survival*) (27). Linear and spline term (pspline, package *survival*, with 4 degrees of freedom [df]) for the gene expression variable were included in the Cox models. Hazard ratio (HR) estimates for the linear terms (Z test) and significance for the spline terms (χ^2 test) were calculated with the summary(type ='inference') method (package *coxExtensions*). No significant associations of the gene expression splines with overall or relapse-free survival could be observed (not shown).
Proportional hazard assumptions of the models (40) and fit statistics (R^2, concordance index [C]) were calculated with the summary(type = 'assumptions') and summary(type ='fit') methods (package *coxExtensions*).
Multi-parameter modeling of overall survival was done in the TCGA KIRC, E-MTAB 1980 and RECA-EU collectives with Ridge regularized Cox regression (package *glmnet*) (29). The explanatory factors included age, squared age, sex, tumor grade (G3/G4 versus G1/G2), tumor stage (T3/T4 versus T1/T2), metastasis stage (M1

versus M0) variables and log_2-transformed expression values of the genes of interest. Two models were trained in the TCGA KIRC cohort: a model with demographic/grade/stage variables only and a model with the complete set of explanatory factors. The values of λ shrinkage metric were found by 200-repeated 10-fold cross validation and corresponded to the optimally regularized models with the lowest deviance (lambda.1se parameter, λ tuning done with cv.glmnet, package *glmnet*) (29). *glmnet* Ridge Cox models were constructed for the optimal λ values.
Subsequently, linear predictor scores for the *glmnet* models were calculated (method predict(), package *glmnet*) for the training TCGA KIRC cohort, and the E-MTAB 1980 and RECA-EU test collectives. Association of the linear predictor scores with overall survival was assessed as described above. To assess the add-on effect of expression of the genes of interest on the survival prediction accuracy as compared with the demographic/grade/stage information, concordance indexes of the full models and the demographic/grade/stage-only models were compared.

Semi-supervised clustering

Two clusters of cancer samples, chemokine low and high, were defined in respect to normalized log_2-transformed expression of the genes of interest in the TCGA KIRC training cohort with PAM (partition around medoids, cosine distance, function kcluster(clust_fun = 'pam'), package *clustTools* employing tools from *philentropy* and *cluster*) (21,22). The clustering algorithm was chosen based on its superior accuracy in 10-fold cross-validation (41) and high fraction of explained clustering variance (ratio of between-cluster sum of squares to total sum of squares) as compared with several other clustering procedures and distance statistics (methods cv() and var(), package *clustTools*,
Supplementary Figure S2A). Choice of the cluster number (k = 2) was motivated by the bend of the within-cluster sum of squares curve and the peak mean silhouette statistic (method plot(type = 'diagnostic'), package *clustTools*, **Supplementary Figure S2B**).
Finally, separation between the clusters was corroborated by a visual analysis of the distance heat map (plot(type ='heat_map'), **Supplementary Figure S2C**). Prediction of the cluster assignment in the test collectives was done with a 15-nearest neighbor (15-NN) classifier with inverse distance weighting (method predict(type = 'propagation')). The prediction yielded clustering structures with comparable fractions of explained variance and comparable cluster sizes in the training TCGA KIRC collective and the test cohorts indicative of high reproducibility of the clustering procedure (**Supplementary Figure 3A**).

Immune cell infiltration, clinical features and survival in the clusters

xCell estimates of non-malignant cell infiltration (9) were compared between the chemokine high and low clusters by Mann-Whitney U test with FDR correction for multiple testing. Differences in distribution of tumor stages (TCGA KIRS, E-MTAB 1980, GSE73731, GSE73731, RECA-EU), MSKCC risk strata (CheckMate) and frequencies of best overall therapy response (complete/partial response versus stable/progressive disease, CheckMate cohorts) between the chemokine clusters were compared by χ_2 test with FDR adjustment for multiple testing. The comparisons were done with the compare_variables() function (*ExDA* package). Differences in

overall and relapse-free survival between the clusters were assessed by Peto-Peto test (functions surv_fit() and surv_pvalue(method = 'S1'), package *survminer*) (28).

Differences in Reactome pathways, gene expression and signaling modulation between the clusters

ssGSEA scores for Reactome pathway gene signatures were calculated for single cancer samples with the calculate() method (package *gseaTools* implementing the original *GSVA* algorithm) (11). Differences in ssGSEA scores between the chemokine high and low clusters were investigated by FDR-adjusted two-tailed T test (test_two_groups(), package *microViz*) (39). The full Reactome pathway signature analysis results are provided in **Supplementary Table S1**.
Genes differentially expressed between the clusters were identified by FDR-corrected twotailed T test (function test_two_groups(), package *microViz*) (39) and > 1.25 foldregulation in the chemokine high cluster as compared with the chemokine low cluster. Numbers of differentially regulated genes are presented in **Supplementary Table S2**. The significantly regulated genes are listed in **Supplementary Table S3**.
Differential modulation of KEGG-listed signaling pathways in chemokine high versus chemokine low cluster cancers based on the regulation estimates for differentially expressed genes was investigated with *SPIA* (30). Significantly activated or inhibited pathways were identified by combined significance for gene set enrichment and net pathway regulation (pG) corrected for multiple testing with FDR method. The tA metric representing the total pathway perturbation was used as an estimate of magnitude of pathway modulation (30,39). Signaling pathways with predicted significant modulation in the chemokine high cluster are listed in **Supplementary Table S4**.

Biochemical reaction modulation in the CXCL9 expression strata

Rules of assignment of genes to biochemical reactions were retrieved from the Recon2 human metabolism model available via the BiGG database (42) and the R package *BiGGR* (31). Estimates of differential expression between chemokine high and chemokine low cluster cancers for all available genes in each cohort were obtained by two-tailed T test as described above. Estimates of biochemical reaction fold-regulation were computed by evaluation of the gene assignment rules following the ‘gene - protein - reaction’ (GPR) principle. The ‘$gene_A$ OR $gene_B$’ operator was interpreted as the arithmetic mean of gene A and gene B estimates of differential expression. The ‘$gene_A$ AND $gene_B$’ operator was interpreted as the minimum of gene A and gene B estimates of differential expression (31).
Standard deviation, 95% confidence intervals and p values for the predicted reaction regulation estimates were obtained by a Monte Carlo simulation with n = 1000 draws from normal distribution of gene expression regulation estimates (mean: differential expression estimate, standard deviation: standard error of the differential expression estimate) (31).
The metabolic models were created with the build_geneSBML() function (package *biggrExtra*). Reaction activity regulation estimates were extracted from the models with the components() method (package *biggrExtra*). Mappings of genes to reactions and reactions to metabolism Recon model subsystems were retrieved with the extract_genes() and extract_subsystems() functions, respectively (package *biggrExtra*).

Reaction names were annotated with the annotate_bigg() function (package *biggrExtra*).
Biochemical reactions with predicted significant modulation in the chemokine high versus chemokine low cluster are listed in **Supplementary Table S5**.

References

1. Creighton CJ, Morgan M, Gunaratne PH, Wheeler DA, Gibbs RA, Robertson G, Chu A, Beroukhim R, Cibulskis K, Signoretti S, et al. Comprehensive molecular characterization of clear cell renal cell carcinoma. *Nature 2013 499:7456* (2013) 499:43–49.
2. Braun DA, Hou Y, Bakouny Z, Ficial M, Sant' Angelo M, Forman J, Ross-Macdonald P, Berger AC, Jegede OA, Elagina L, et al. Interplay of somatic alterations and immune infiltration modulates response to PD-1 blockade in advanced clear cell renal cell carcinoma. *Nature medicine* (2020) 26:909.
3. RECA. RECA-EU, ICGC Data Portal. https://dcc.icgc.org/projects/RECA-EU [Accessed August 16, 2022]
4. Wei X, Choudhury Y, Lim WK, Anema J, Kahnoski RJ, Lane B, Ludlow J, Takahashi M, Kanayama HO, Belldegrun A, et al. Recognizing the Continuous Nature of Expression Heterogeneity and Clinical Outcomes in Clear Cell Renal Cell Carcinoma. *Scientific reports* (2017) 7: doi: 10.1038/S41598-017-07191-Y
5. Laskar RS, Li P, Ecsedi S, Abedi-Ardekani B, Durand G, Robinot N, Hubert JN, Janout V, Zaridze D, Mukeria A, et al. Sexual dimorphism in cancer: insights from transcriptional signatures in kidney tissue and renal cell carcinoma. *Human molecular genetics* (2021) 30:343–355.
6. Sean D, Meltzer PS. GEOquery: a bridge between the Gene Expression Omnibus (GEO) and BioConductor. *Bioinformatics (Oxford, England)* (2007) 23:1846–1847.
7. Sato Y, Yoshizato T, Shiraishi Y, Maekawa S, Okuno Y, Kamura T, Shimamura T, Sato-Otsubo A, Nagae G, Suzuki H, et al. Integrated molecular analysis of clear-cell renal cell carcinoma. *Nature Genetics 2013 45:8* (2013) 45:860–867.
8. Finotello F, Mayer C, Plattner C, Laschober G, Rieder Di, Hackl H, Krogsdam A, Loncova Z, Posch W, Wilflingseder D, et al. Molecular and pharmacological modulators of the tumor immune contexture revealed by deconvolution of RNA-seq data. *Genome Medicine* (2019) 11:34.
9. Aran D, Hu Z, Butte AJ. xCell: Digitally portraying the tissue cellular heterogeneity landscape. *Genome Biology* (2017) 18:220.
10. Sturm G, Finotello F, List M. Immunedeconv: An R Package for Unified Access to Computational Methods for Estimating Immune Cell Fractions from Bulk RNA-Sequencing Data. *Methods in molecular biology (Clifton, NJ)* (2020) 2120:223–232.
11. Hänzelmann S, Castelo R, Guinney J. GSVA: Gene set variation analysis for microarray and RNA-Seq data. *BMC Bioinformatics* (2013) 14:7.
12. Cai MC, Zhao X, Cao M, Ma P, Chen M, Wu J, Jia C, He C, Fu Y, Tan L, et al. T-cell exhaustion interrelates with immune cytolytic activity to shape the inflamed tumor microenvironment. *The Journal of Pathology* (2020) 251:147–159.
13. Woroniecka K, Chongsathidkiet P, Rhodin K, Kemeny H, Dechant C, Harrison Farber S, Elsamadicy AA, Cui X, Koyama S, Jackson C, et al. T-cell exhaustion signatures vary with tumor type and are severe in glioblastoma. *Clinical Cancer Research* (2018) 24:4175–4186.
14. Kocher F, Puccini A, Untergasser G, Martowicz A, Zimmer K, Pircher A, Baca Y, Xiu J, Haybaeck J, Tymoszuk P, et al. Multi-omic Characterization of Pancreatic Ductal Adenocarcinoma Relates CXCR4 mRNA Expression Levels to Potential Clinical Targets. *Clinical Cancer Research* (2022) 28:4957–4967.
15. Zhang Z, Chen L, Chen H, Zhao J, Li K, Sun J, Zhou M. Pan-cancer landscape of T-cell exhaustion heterogeneity within the tumor microenvironment revealed a progressive roadmap of hierarchical dysfunction associated with prognosis and therapeutic efficacy. *eBioMedicine* (2022) 83:104207.

16. Wickham H, Averick M, Bryan J, Chang W, McGowan L, François R, Grolemund G, Hayes A, Henry L, Hester J, et al. Welcome to the Tidyverse. *Journal of Open Source Software* (2019) 4:1686.
17. Gagolewski M, Tartanus B. Package 'stringi'. (2021) https://cran.r-project.org/web/packages/stringi/index.html http://cran.ism.ac.jp/web/packages/stringi/stringi.pdf
18. Vaughan D, Dancho M, RStudio. furrr: Apply Mapping Functions in Parallel using Futures. (2022) https://cran.r-project.org/package=furrr
19. Csardi G, Nepusz T. The igraph software package for complex network research. *InterJournal* (2006) Complex Sy:1695. https://igraph.org
20. Butts CT, Hunter D, Handcock M, Bender-deMoll S, Horner J, Wang L, Krivitsky PN, Knapp B, Bojanowski M, Klumb C. network: Classes for Relational Data. (2021) https://cran.r-project.org/package=network
21. Schubert E, Rousseeuw PJ. Faster k-Medoids Clustering: Improving the PAM, CLARA, and CLARANS Algorithms. *Lecture notes in computer science (including subseries lecture notes in artificial intelligence and lecture notes in bioinformatics)*. Springer (2019). p. 171–187 doi: 10.1007/978-3-030-32047-8_16
22. Drost H-G. Philentropy: Information Theory and Distance Quantification with R. *Journal of Open Source Software* (2018) 3:765. doi: 10.21105/joss.00765
23. Kassambara A, Mundt F. factoextra: Extract and Visualize the Results of Multivariate Data Analyses. (2020) https://cran.r-project.org/web/packages/factoextra/index.html
24. Hahsler M, Piekenbrock M, Doran D. Dbscan: Fast density-based clustering with R. *Journal of Statistical Software* (2019) 91:1–30.
25. Konopka T. umap: Uniform Manifold Approximation and Projection. (2022) https://cran.r-project.org/web/packages/umap/index.html
26. Kassambara A. rstatix: Pipe-Friendly Framework for Basic Statistical Tests. (2021) https://cran.r-project.org/package=rstatix
27. Therneau TM, Grambsch PM. *Modeling Survival Data: Extending the Cox Model*. 1st ed. New York: Springer Verlag (2000).
28. Kassambara A, Kosinski M, Biecek P. survminer: Drawing Survival Curves using 'ggplot2'. (2016) https://cran.r-project.org/package=survminer
29. Friedman J, Hastie T, Tibshirani R. Regularization paths for generalized linear models via coordinate descent. *Journal of Statistical Software* (2010) 33:1–22.
30. Tarca AL, Draghici S, Khatri P, Hassan SS, Mittal P, Kim JS, Kim CJ, Kusanovic JP, Romero R. A novel signaling pathway impact analysis. *Bioinformatics* (2009) 25:75–82.
31. Gavai AK, Supandi F, Hettling H, Murrell P, Leunissen JAM, Van Beek JHGM. Using Bioconductor Package BiGGR for Metabolic Flux Estimation Based on Gene Expression Changes in Brain. *PLOS ONE* (2015) 10:e0119016.
32. Wickham Hadley. *ggplot2: Elegant Graphics for Data Analysis*. 1st ed. New York: Springer-Verlag (2016). https://ggplot2.tidyverse.org
33. Wilke CO. *Fundamentals of Data Visualization: A Primer on Making Informative and Compelling Figures*. 1st ed. Sebastopol: O'Reilly Media (2019).
34. Briatte F, Bojanowski M, Canouil M, Charlop-Powers Z, Fisher JC, Johnson K, Rinker T. ggnetwork: Geometries to Plot Networks with 'ggplot2'. (2021) https://cran.rproject.org/package=ggnetwork
35. Allaire J, Xie Y, McPherson J, Luraschi J, Ushey K, Atkins A, Wickham H, Cheng J. rmarkdown: Dynamic Documents for R. (2022) https://cran.r-project.org/web/packages/rmarkdown/index.html
36. Xie Y. *Bookdown: Authoring books and technical documents with R Markdown*. (2016). doi: 10.1201/9781315204963

37. Xie Y. knitr: A General-Purpose Package for Dynamic Report Generation in R. (2022) https://cran.r-project.org/web/packages/knitr/index.html
38. Gohel D. flextable: Functions for Tabular Reporting. (2022) https://cran.rproject.org/web/packages/flextable/index.html
39. Benjamini Y, Hochberg Y. Controlling the False Discovery Rate: A Practical and Powerful Approach to Multiple Testing. *Journal of the Royal Statistical Society: Series B (Methodological)* (1995) 57:289–300.
40. Grambsch PM, Therneau TM. Proportional Hazards Tests and Diagnostics Based on Weighted Residuals. *Biometrika* (1994) 81:515.
41. Lange T, Roth V, Braun ML, Buhmann JM. Stability-based validation of clustering solutions. *Neural Computation* (2004) 16:1299–1323
42. King ZA, Lu J, Dräger A, Miller P, Federowicz S, Lerman JA, Ebrahim A, Palsson BO, Lewis NE. BiGG Models: A platform for integrating, standardizing and sharing genome-scale models. *Nucleic Acids Research* (2016) 44:D515–D522.
